# Merging Halogen-Atom Transfer (XAT) and Copper Catalysis for the Modular Suzuki–Miyaura-Type Cross-Coupling of Alkyl Iodides and Organoborons

**DOI:** 10.1021/jacs.1c12649

**Published:** 2022-01-21

**Authors:** Zhenhua Zhang, Bartosz Górski, Daniele Leonori

**Affiliations:** Department of Chemistry, University of Manchester, Oxford Road, Manchester M13 9PL, U.K.

## Abstract

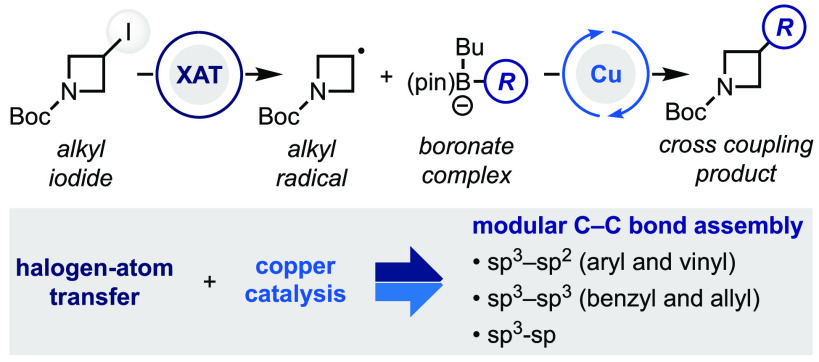

We report here a mechanistically distinct approach to achieve Suzuki–Miyaura-type cross-couplings between alkyl iodides and aryl organoborons. This process requires a copper catalyst but, in contrast with previous approaches based on palladium and nickel systems, does not utilizes the metal for the activation of the alkyl electrophile. Instead, this strategy exploits the halogen-atom-transfer ability of α-aminoalkyl radicals to convert secondary alkyl iodides into the corresponding alkyl radicals that then are coupled with aryl, vinyl, alkynyl, benzyl, and allyl boronate species. These novel coupling reactions feature a simple setup and conditions (1 h at room temperature) and facilitate access to privileged motifs targeted by the pharmaceutical sector.

## Introduction

Among all cross-coupling approaches, the Nobel-Prize-winning Suzuki–Miyaura reaction has changed the way organic molecules are assembled.^1^ This process is widely used in both industrial and academic settings mostly due to its mild conditions and the commercial availability of both organic halides and organoboron building blocks.^2^ However, while aryl halides are a benchmarked class of coupling partners, the utilization of alkyl halides is less straightforward. Under palladium catalysis, the slower rate of oxidative addition and the increased chances of β-hydride elimination often render these reactions difficult to implement.^3^

**Scheme 1:**
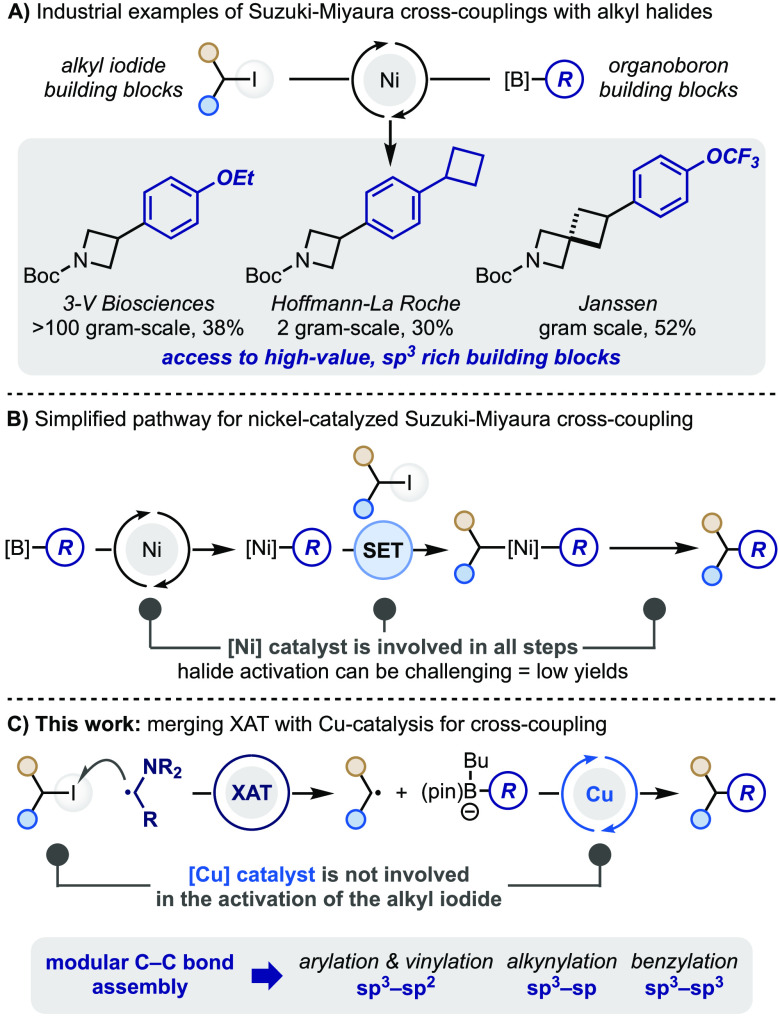
(A) The Transition-Metal-Catalyzed (Mostly Nickel) Cross-Coupling between Alkyl Halide and Organoboron Building Blocks Is Often Used to Access sp^3^-Rich Materials, (B) Current Methods Require the Nickel Catalyst to Be Involved in the Halide Activation Step That Can Be Challenging, and (C) This Work Exploits α-Aminoalkyl Radical-Mediated XAT to Activate the Halide and Uses a Copper Catalyst

Nickel catalysis has provided a workable solution to this challenge through the use of catalysts supported by either phenanthroline or aminoalcohol ligands.^4^ Other base metals (mostly Fe and Co) have been successfully applied in Suzuki-type cross-coupling, but their reactivity is less general.^5^ Overall, despite significant success in the area, coupling with secondary and unactivated alkyl electrophiles is still a relevant challenge, as these processes are usually low yielding, especially when polar functionalities are present.^6^ Notwithstanding these challenges and often suboptimal yields, the synthetic value provided by these coupling reactions makes them widely applied by the pharmaceutical and agrochemical sectors, where sp^3^-rich fragments have increased chances of biological activity (Scheme 1A).^7^ The development of novel strategies increasing our synthetic capacity for the assembly of these motifs has the potential to affect the discovery and manufacture of materials that ultimately can improve the quality of our lives.^8^

The cross-coupling strategies discussed above revolve around the direct reaction of the nickel catalyst with an alkyl halide, which generally leads to the formation of transient radical species (Scheme 1B).^9^ While this might help in the case of difficult oxidative additions, it also means that the catalyst properties need to be carefully balanced to enhance halide activation without compromising the following elementary steps such as transmetalation and reductive elimination.^6a,10^

We recently speculated that a conceptually different strategy, where the metal catalyst is required to orchestrate the C–C bond formation but not to activate the alkyl halide, might provide synthetic advantages toward the assembly of challenging small-molecule building blocks. In this paper we report the realization of this goal and present a novel and general approach for the Suzuki–Miyaura-type cross-coupling between secondary alkyl iodides and a broad range of boronates. This method integrates α-aminoalkyl radical mediated halogen-atom transfer (XAT) with copper catalysis^11^ and provides a general entry into the modular assembly of challenging C(sp^3^)–C(sp^2^) as well as C(sp^3^)–C(sp^3^) and C(sp^3^)–C(sp) bonds.

## Results and Discussion

We and the Doyle group have recently reported that alkyl and aryl iodides can be converted into the corresponding radicals via α-aminoalkyl radical mediated XAT.^12^ This reactivity benefits from a polarized transition state where the α-aminoalkyl unit stabilizes charge transfer, which kinetically accelerates the halide abstraction. This blueprint for radical generation can be exploited in different settings, which include the coupling of alkyl iodides with N-nucleophiles to assemble S_N_2-elusive C(sp^3^)–N bonds.^13^ This reactivity paradigm is possible by merging XAT with copper catalysis so that an alkyl radical is generated and then captured by a copper-bound N fragment.

In order to achieve C–C bond formation, we looked at the Chan–Lam cross-coupling that has pioneered the ability of aryl organoborons to undergo transmetalation with [Cu(II)] species.^14^ We therefore envisaged that the merger of XAT with [Cu] catalysis might enable a hybrid type of cross-coupling, which would be of the Suzuki type in terms of retrosynthetic disconnection but more Chan–Lam based in terms of mechanism (Scheme 1C).

**Scheme 2:**
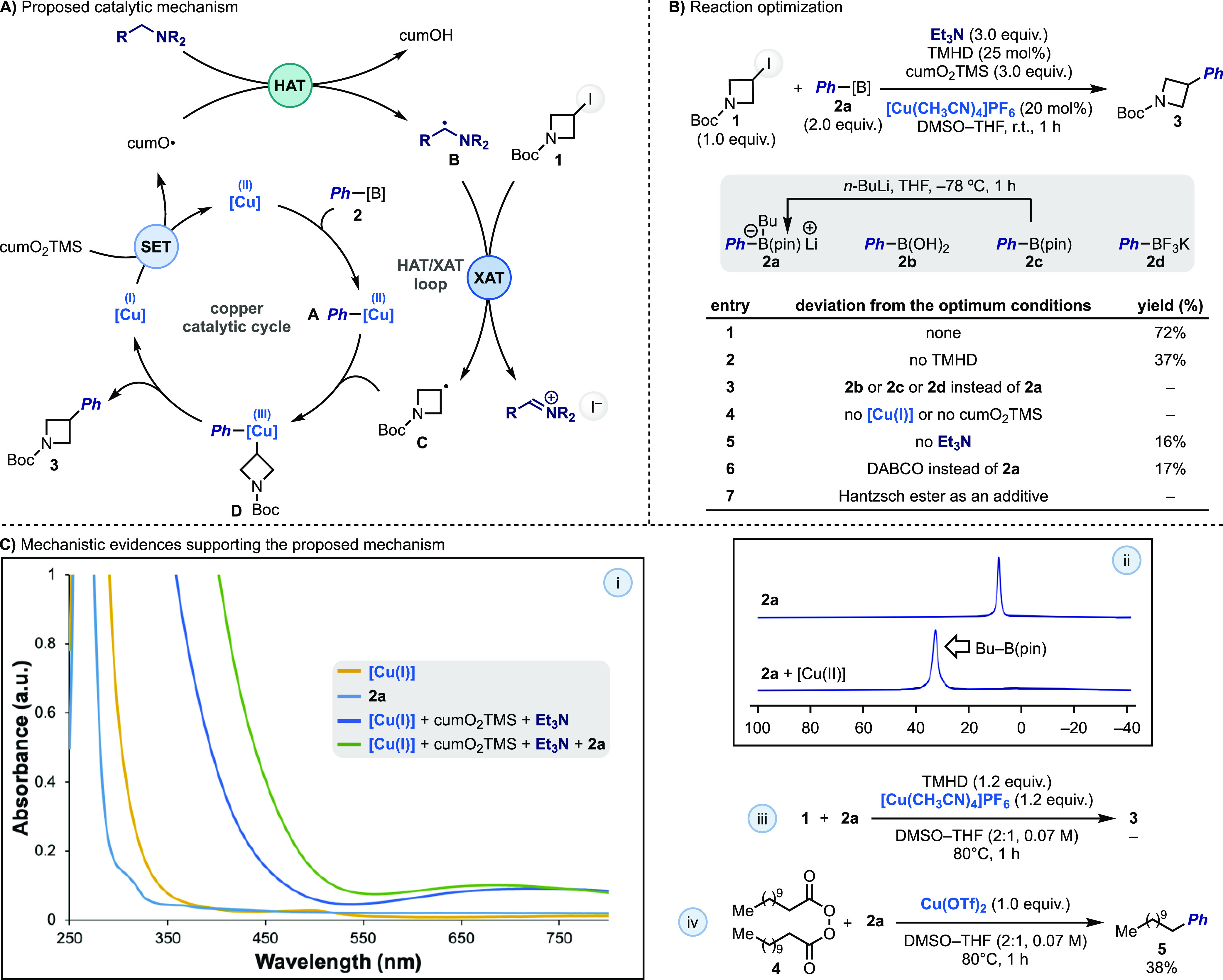
(A) Proposed Mechanism for the XAT-Mediated and Cu-Catalyzed Cross-Coupling of Alkyl Iodides and Aryl Organoborons, (B) Reaction Optimization and Key Control Experiments, and (C) Selected Mechanistic Experiments

Our proposed pathway for such XAT-mediated and Cu-catalyzed arylation is illustrated in Scheme 2A using 3-iodo-*N*-Boc-azetidine **1** and the generic Ph-organoboron derivative **2** as the coupling partners. Starting from a [Cu(I)] catalyst, ground-state SET with a stoichiometric oxidant such as cumO_2_TMS would deliver a Cu(II)] species that would transmetalate with **2** and give the Ph–[Cu(II)] complex **A**. The cumO^•^ generated in the SET event would react with a stoichiometric amine reagent to give, upon polarity-matched H atom transfer (HAT),^15^ the key nucleophilic α-aminoalkyl radical **B**. Polarity-accelerated XAT between **B** and **1** would then be used to access the alkyl radical **C**, which could enter the [Cu(I/II/III)]-catalytic cycle and trap **A**.^16^ This step should deliver the alkyl,aryl–[Cu(III)] intermediate **D**, from which reductive elimination is facile^17^ and should therefore provide the cross-coupling product **3** while reinitiating the catalytic cycle.

As was mentioned before, the key mechanistic difference between this approach and other metal-mediated cross-couplings is that the alkyl radical generation occurs in a HAT/XAT loop that is dissected by the copper-based catalytic cycle. This means that the alkyl halide does not need to engage in direct SET with the metal, which can be difficult due to its low reduction potential (for alkyl–I: *E*^red^ < −2 V vs SCE), or enter its coordination sphere for subsequent abstraction (or oxidative addition). This fundamental difference obviates the need of a highly reducing catalyst, which we hoped might translate into a broader substrate scope.

This proposal for the radical arylation of **1** was eventually implemented using boronate **2a** as the coupling partner (prepared by addition of *n*-BuLi to **2c** at −78 °C), [Cu(CH_3_CN)_4_]PF_6_ as the catalyst, Et_3_N as the XAT promoter, cumO_2_TMS as the oxidant, and 2,2,6,6-tetramethyl-3,5-heptanedione (TMHD) as the ligand in a DMSO–THF solvent mixture at room temperature (Scheme 2B, entry 1).^18^ Under these mild conditions, **3** was obtained in 72% yield in just 1 h. The TMHD ligand was important to improve the yield in this specific example (Scheme 2B, entry 2), but it was not necessary for all the cross-couplings presented below. Less activated organoborons (e.g., **2b**–**d**) were evaluated, but they did not lead to product formation (Scheme 2B, entry 3), which we propose might result from their lower ability to transmetalate with [Cu(II)] species.^18^

In the absence of [Cu(I)] catalyst or the oxidant, no reactivity took place (Scheme 2B, entry 4) while the exclusion of Et_3_N provided **3** in 17% yield (Scheme 2B, entry 4). We were initially surprised by the success of this reaction, as the activation of **1** should not occur. An analysis of the crude reaction mixture revealed the trace formation of Ph–I and acetophenone. We propose that under these amine-free conditions other productive pathways based on SET oxidation of **2a** to the corresponding Ph^•^ and/or fragmentation of **C** to Me^•^ might be operating. These reactivities would generate XAT-active species that can homolytically activate **1** and lead to product formation.^18^

To obtain more details on the process, we ran some mechanistic studies. UV/vis absorption spectroscopy studies demonstrated that [Cu(CH_3_CN)_4_]PF_6_ is oxidized upon treatment with cumO_2_TMS (new absorption band at λ ≈ 650 nm)^19^ and that transmetalation with **2a** might be occurring (Scheme 2C-i). ^11^B NMR spectroscopy studies were used to further support the transmetalation of **2a** with [Cu(II)], as evidenced by the formation of BuB(pin) (Scheme 2C-ii). The requirement of **B** to achieve XAT activation of **1** was revealed by the fact that replacing Et_3_N for DABCO, an amine that can be oxidized but cannot lead to the formation of an α-aminoalkyl radical,^20^ led to **3** in 17% yield, which is identical with the outcome observed under amine-free conditions (Scheme 2B, entry 6). Furthermore, the fact that a stoichiometric reaction of **1**, **2a**, and [Cu(I)] did not lead to any product formation demonstrates that a putative Ph–[Cu(I)] intermediate is not able to activate the alkyl halide by either SET or oxidative addition (Scheme 2C-iii). This clearly underscores the relevance of XAT as the alkyl iodide activation pathway. Finally, the thermal reaction of lauroyl peroxide **4** with **2a** using a stoichiometric [Cu(II)] species gave **5** in 38% yield (Scheme 2C-iv). This experiment supports the generation of a primary alkyl radical (O–O homolysis then decarboxylation) that can intercept **A** and therefore enable the coupling process.^18^

**Scheme 3:**
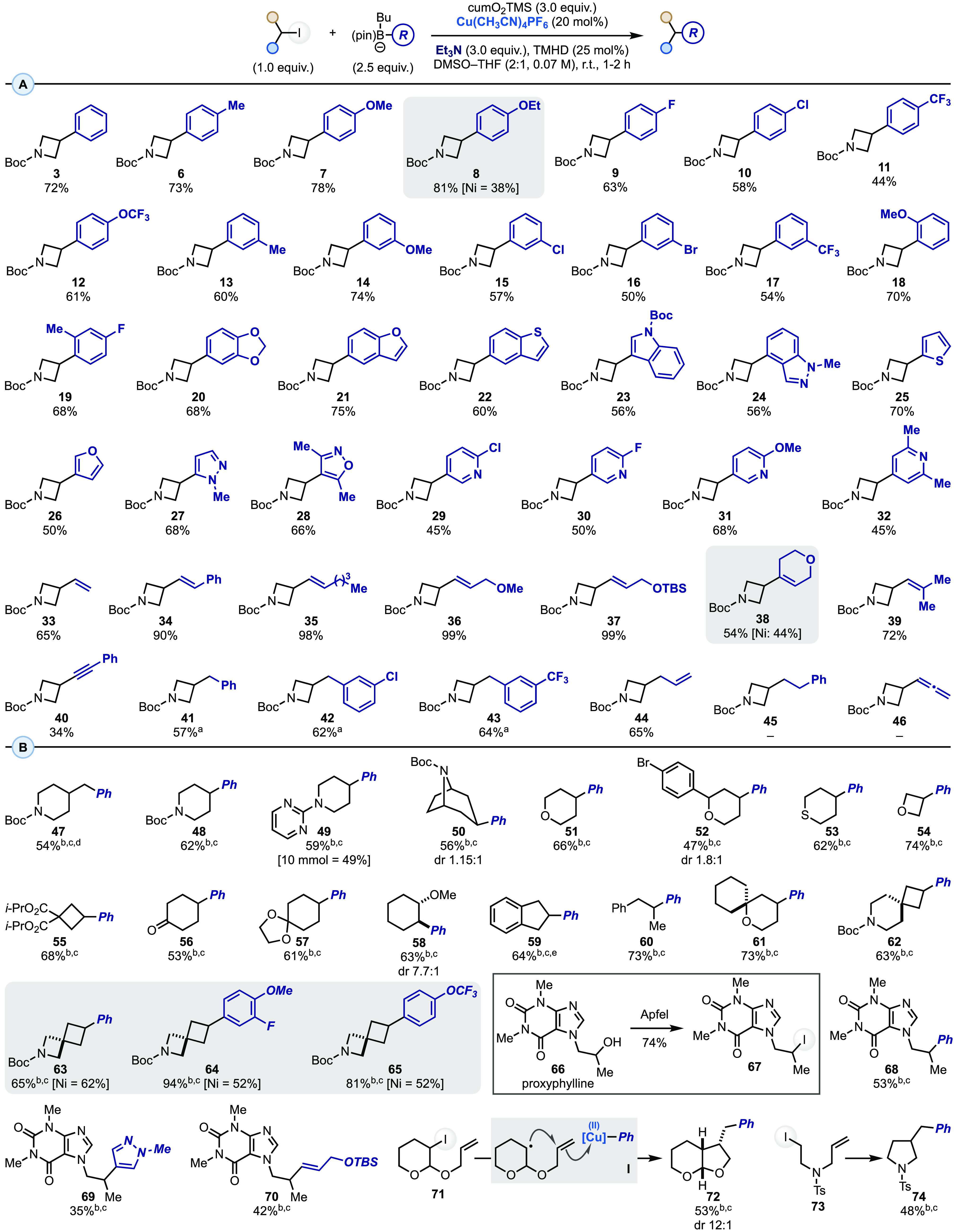
XAT-Mediated and Cu-Catalyzed Cross-Coupling of Alkyl Iodides and Aryl Organoborons: (A) Organoboron Scope and Limitations and (B) Alkyl Iodide Scope and Limitations cumO_2_Si*t*-BuPh was used in place of cumO_2_TMS. cumO_2_TES was used in place of cumO_2_TMS. Reaction run with no TMHD. 10 mol % of Cu(CH_3_CN)_4_PF_6_. 30 mol % of Cu(CH_3_CN)_4_PF_6_.

With the optimized reaction conditions in hand, we evaluated the scope of the transformation using **1** as the model alkyl iodide (Scheme 3A). We started by evaluating *para*-substituted aryl boronates and found that a variety of substituents were tolerated, delivering the desired products in good yields. These included substrates with electron-rich Me (**6**), OMe (**7**) and OEt (**8**) groups as well as electron-withdrawing Cl (**9**), F (**10**), CF_3_ (**11**), and OCF_3_ (**12**) functionalities. A similar trend was observed for the utilization of *meta*- and *ortho*-substituted derivatives (**13**–**17** and **18**, respectively), which also included aryl chloride and bromide functionalities that can be engaged in further modular diversifications.

Polysubstituted aryl boronates were screened next, and they provided the desired products in good yields (**19** and **20**). Electron-rich heteroaryl boronates were competent coupling partners, and they enabled the introduction of medicinally relevant benzofuran (**21**), bezothiophene (**22**), indole (**23**), and indazole (**24**) units, as well as thiophene (**25**), furan (**26**), pyrazole (**27**), and isoxazole (**28**). Pyridines are some of the most prevalent motifs in bioactive leads,^21^ and pleasingly, our copper-catalyzed approach successfully engaged both C-3 (**29**–**31**) and C-4 (**32**) borylated derivatives.

Having benchmarked this reactivity on a diverse set of aromatic coupling partners, we evaluated its feasibility with respect to vinyl derivatives. Pleasingly, the utilization of several commercial boronic esters enabled, upon boronate formation, the introduction of vinyl (**33**) and styrenenyl (**34**) as well as other mono- and disubstituted olefin (**35**–**37** and **38**, **39** respectively) units in high yields. The initial results demonstrated that alkynyl boronates are viable partners (**40**) to achieve C(sp^3^)–C(sp) bond formation, albeit in lower yields.

The formation of C(sp^3^)–C(sp^3^) linkages via cross-coupling strategies is still a challenging task. We were pleased to find that activated benzylic^22^ and allylic boronates performed well under the reaction conditions, delivering **41**–**43** and **44** in good yields. In terms of limitations, allenyl and unactivated alkyl boronates failed to to provide the desired products (**45** and **46**).

Of the substrates presented in Scheme 3A, **8** and **38** have been recently prepared by the pharmaceutical sector (**8**, 3 V-Biosciences;^23^ and **38**, GSK^24^) by Ni-catalyzed Suzuki–Miyaura cross-coupling on **1**. The approach reported here utilized the same iodide and provided the desired products in higher yields. We hope this might highlight the complementarity that this strategy can provide to mainstream approaches in the case of challenging arylations.

Evaluation of the alkyl iodide scope was performed using the Ph–B(pin)-based boronate **2a** as the coupling partner, which revealed that a wide range of unactivated alkyl iodides can be engaged (Scheme 3B). This was showcased by the arylation of several piperidine derivatives, either *N*-Boc protected (**47** and **48**) or part of a 2-aminopyrimidine unit (**49**, also on a 10 mmol scale). The chemistry was then applied to the preparation of **50**, which is an analogue of the alkaloid nortropine. Other commercial small-molecule building blocks were successfully engaged, as demonstrated by the arylation of 4-iodo(thio)pyrans (**51**–**53**), 3-iodooxetane (**54**), and a cyclobutene derivative (**55**) recently disclosed by Merck for the preparation of trifluoromethylated cyclobutanes.^25^

The coupling reactivity was compatible with ketone, acetal, and ether functionalities (**56**–**59**), while the formation of **59** and **60** demonstrated that HAT-labile benzylic positions are tolerated. This chemoselectivity is noteworthy, considering the ability of cumO^•^ to promote HAT reactions on activated benzylic C(sp^3^)–H positions.^26^

Spirocycles are interesting chemotypes in drug development campaigns due to their high C(sp^3^) content.^27^ Pleasingly, radical cross-couplings with several commercially available iodides were high-yielding (**61**–**65**). Of these substrates, **63**–**65** have been recently prepared by Janssen using Ni catalysis on the corresponding iodides, albeit in lower yields and, in the case of **65**, as a mixture with the demethoxylated product,^28^ which was not observed under our conditions.

Complex alkyl iodides can be easily generated by Appel reactions on secondary alcohols, which are a large class of commercial materials. We therefore converted the cardiac stimulant proxyphylline (**66**) into linear alkyl iodide **67** and demonstrated that this derivative can undergo divergent arylation and alkenylation reactivity (**68**–**70**).

Finally, all the examples presented so far have dealt with the direct arylation of a secondary alkyl iodide. An interesting avenue for molecular construction offered by the utilization of carbon radicals is that these species can undergo other types of reactivities before engaging in the final cross-coupling event. This might provide a synthetic opportunity with respect to standard Suzuki–Miyaura reactivity, as simplified building blocks can be used to access more complex products. A preliminary illustration of this was demonstrated by the use of **71** and **73** that upon XAT activation underwent 5*-exo-trig* cyclization onto the tethered olefin followed by Cu-catalyzed arylation (i.e., **I**) to give **72** and **74** in useful yields.

## Conclusions

In conclusion, the results reported here demonstrate that α-aminoalkyl radical mediated halogen-atom transfer can be integrated with copper catalysis to enable the modular assembly of C–C bonds. This reactivity provides a mechanistically distinct tactic to engage alkyl iodides in general cross-coupling reactions with aryl, vinyl, alkynyl, benzyl, and allyl organborons. Future developments will be aimed at engaging unactivated alkyl organometallic partners as well as translating the chemistry into asymmetric settings.

## Supporting Information Available

Experimental procedures, optimization and mechanistic studies, and characterization data (PDF)
