# Supplementary material for: Merging Halogen-Atom Transfer (XAT) and Copper Catalysis for the Modular Suzuki–Miyaura-Type Cross-Coupling of Alkyl Iodides and Organoborons
Source: J Am Chem Soc. 2022 Jan 21;144(4):1986–92. doi: 10.1021/jacs.1c12649 (PMC9098170; doi:10.1021/jacs.1c12649)
Supplement: Supplementary file 1 — ja1c12649_si_001.pdf [file ja1c12649_si_001.pdf]

Merging Halogen-Atom Transfer (XAT) with Copper Catalysis for the Modular Suzuki-Miyaura-Type Cross-Coupling of Alkyl Iodides and Organoborons

Zhenhua Zhang,[1†] Bartosz Górski,[1†] and Daniele Leonori[1]*

[1] *Department of Chemistry, University of Manchester, Oxford Road, Manchester M13 9PL, UK.*

* daniele.leonori@manchester.ac.uk

† These authors contributed equally to this work.

Table of Contents

1 General Experimental Details 3
2 Starting Material Synthesis 4
3 Preparation of the Oxidants 10
4 Preparation of Boronate Complexes 12
5 Reaction Optimization 13
6 Control Experiments 17
7 Substrate Scope 19
8 Mechanistic Studies 40
8.1 Proposed Mechanism 40
8.1.1 Probing the Oxidation of [Cu(I)] 40
8.1.2 Probing the Transmetalation of 2a with [Cu(II)] 41
8.1.3 SET Oxidation of 2a 42
8.1.4 Probing α-Aminoalkyl Radical Generation by HAT 43
8.1.5 Probing α-Aminoalkyl Radical-Mediated XAT 44
8.1.6 Ruling Out Direct SET Reduction of the Alkyl Iodide 45
8.1.7 Probing Radical Capture by [Cu(II)]–Ar Species as C(sp^3)–C(sp^2) Bond Forming Step 45
8.2 Alternative Mechanism 47
8.2.1 Coordination of 2a to [Cu(I)] to Give [Cu(I)]–Ph 47

8.2.2 Oxidation of [Cu(I)]–Ph by cumO_2TMS to [Cu(II)]–Ph 48
9 Cyclic Voltammetry Studies 49
10 NMR Spectra 50
11 Reference 84

1 General Experimental Details

All required fine chemicals were used directly without purification unless stated otherwise. All air and moisture sensitive reactions were carried out under nitrogen atmosphere using standard Schlenk manifold technique. All solvents were bought from Acros as 99.8% purity. 1H and 13C Nuclear Magnetic Resonance (NMR) spectra were acquired at various field strengths as indicated and were referenced to $CHCl_3$ (7.26 and 77.0 ppm for ^1H and ^{13}C respectively). 1H NMR coupling constants are reported in Hertz and refer to apparent multiplicities and not true coupling constants. Data are reported as follows: chemical shift, integration, multiplicity (s = singlet, br s = broad singlet, d = doublet, t = triplet, q = quartet, p = pentet, h = hextet, hept = heptet, m = multiplet, dd = doublet of doublets, etc.), proton assignment (determined by ^2D NMR experiments: COSY, HSQC and HMBC) where possible. High-resolution mass spectra were obtained using a JEOL JMS-700 spectrometer or a Fissions VG Trio 2000 quadrupole mass spectrometer. Spectra were obtained using electron impact ionization (EI) and chemical ionization (CI) techniques, or positive electrospray (ES). Analytical TLC: aluminum backed plates pre-coated (0.25 mm) with Merck Silica Gel 60 F254. Compounds were visualized by exposure to UV-light or by dipping the plates in permanganate (KMnO4) stain followed by heating. Flash column chromatography was performed using Merck Silica Gel 60 (40–63 μm). All mixed solvent eluents are reported as v/v solutions. UV-visible absorption spectra were obtained using a Horiba Duetta spectrometer and 1 mm High Precision Cell made of quartz from Hellma Analytics (*c* = 5 mM). All the reactions were conducted in CEM 10 mL glass microwave tubes.

Some abbreviations used:

cumOOTMS: trimethylsilyl(cumyl) peroxide

cumOOTES: triethylsilyl(cumyl) peroxide

cumOOSiPh$_2$*t*Bu: *t*-butyldiphenyl(cumyl) peroxide

TMHD: 2,2,6,6-tetramethyl-3,5-heptanedione

RuPhos: 2-dicyclohexylphosphino-2′,6′-diisopropoxybiphenyl

BINOL: 1,1′-bi(2-naphthol)

TMEDA: *N,N,N′,N′*-tetramethyl ethylenediamine

Xantophos: 4,5-bis(diphenylphosphino)-9,9-dimethylxanthene

dtbbpy: 4,4′-di-*t*-butyl-2,2′-dipyridyl

CuTc: copper(I) thiophene-2-carboxylate

CPME: cyclopentyl methyl ether

2 Starting Material Synthesis

General Procedure of the Appel Reaction – GP1

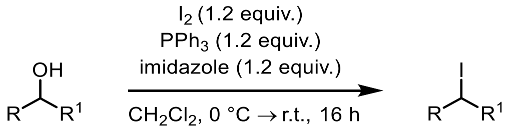

A round-bottom flask equipped with a stirring bar was charged with the corresponding alcohol (1.0 equiv.), Ph_3P (1.2 equiv.) and imidazole (1.2 equiv.). The flask was evacuated and refilled with N_2. CH_2Cl_2 (0.1 M) was added, and the reaction was cooled to 0 °C with an ice-water bath. I_2 (1.2 equiv.) was added portion-wise and then the cooling bath was removed. The reaction was stirred 16 hours at room temperature and then diluted with H_2O (30 mL). The layers were separated and the aqueous layer was extracted with CH_2Cl_2 (3 x 30 mL). The combined organic layers were washed with $Na_2S_2O_3$ (30 mL, saturated), brine (30 mL), dried ($MgSO_4$), filtered and evaporated. Purification by flash column chromatography on silica gel gave the products.

Diisopropyl 3-Iodocyclobutane-1,1-dicarboxylate (S1)

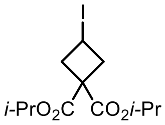

Following **GP1**, diisopropyl 3-hydroxycyclobutane-1,1-dicarboxylate (1.22 g, 5.0 mmol) gave **S1** (1.45 mg, 82%) as an oil. ^{1}H NMR (400 MHz, $CDCl_3$) δ 5.07 (1H, hept, *J* = 6.3 Hz), 5.06 (1H, hept, *J* = 6.3 Hz), 4.52 (1H, p, *J* = 8.4 Hz), 3.24–3.11 (2H, m), 3.08–2.95 (2H, m), 1.24 (6H, d, *J* = 2.1 Hz), 1.23 (6H, d, *J* = 2.1 Hz); ^{13}C NMR (101 MHz, $CDCl_3$) δ 170.4, 169.0, 69.5, 69.3, 54.2, 42.1, 21.5, 6.7; HRMS (ASAP): Found MH^+ 355.0398, $C_{12}H_{20}O_4I$ requires 355.0398.

(2-Iodopropyl)benzene (S2)

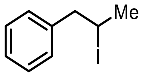

Following **GP1**, 1-phenylpropan-2-ol (1.36 g, 10.0 mmol) gave **S2** as an oil (1.19 g, 48%). ^{1}H NMR (400 MHz, $CDCl_3$) δ 7.32-7.24 (3H, m), 7.17 (2H, d, *J* = 6.5 Hz), 4.33 (1H, h, *J* = 6.8 Hz), 3.31–3.26 (1H, m), 3.08–3.03 (1H, m), 1.89 (3H, d, *J* = 6.5 Hz). Data in accordance with the literature.[1]

8-Iodo-1,4-dioxaspiro[4.5]decane (S3)

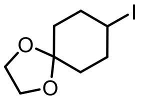

Following **GP1** 1,4-dioxaspiro[4.5]decan-8-ol (0.79 g, 5.0 mmol) gave **S3** as an oil (0.99 g, 74%). ^{1}H NMR (400 MHz, $CDCl_3$) δ 4.49–4.32 (1H, m), 4.01–3.83 (4H, m), 2.15–2.02 (4H, m), 1.80 (2H, ddd, *J* = 12.4, 7.7, 4.9 Hz), 1.60 (2H, ddd, *J* = 13.1, 8.3, 4.6 Hz). Data in accordance with the literature.[2]

4-Iodotetrahydro-2*H*-thiopyran (S4)

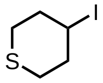

Following **GP1**, tetrahydrothiopyran-4-ol (0.96 g, 8.2 mmol) gave **S4** as an oil (1.41 g, 75%). ^{1}H NMR (400 MHz, $CDCl_3$) δ 4.55–4.40 (1H, m), 2.90–2.74 (2H, m), 2.65–2.45 (2H, m), 2.40–2.15 (4H, m). Data in accordance with the literature.[3]

***tert*-Butyl 6-Iodo-2-azaspiro[3.3]heptane-2-carboxylate (S5)**

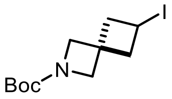

Following **GP1**, *tert*-butyl 6-hydroxy-2-azaspiro[3.3]heptane-2-carboxylate (0.85 g, 4.0 mmol) gave **S5** as a solid (0.97 g, 75%). ^{1}H NMR (400 MHz, $CDCl_3$) δ 4.29 (1H, p, *J* = 7.8 Hz), 3.94 (4H, d, *J* = 12.3 Hz), 2.96–2.87 (2H, m), 2.74–2.66 (2H, m), 1.42 (9H, s). Data in accordance with the literature.[4]

***tert*-Butyl 2-Iodo-7-azaspiro[3.5]nonane-7-carboxylate (S6)**

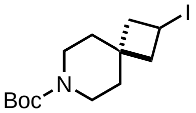

Following **GP1**, but using toluene as a solvent, reflux, 16 h, *tert*-butyl 2-hydroxy-7-azaspiro[3.5]nonane-7-carboxylate (0.96 g, 4.0 mmol) gave **S6** as a solid (0.96 g, 68%). ^{1}H NMR (400 MHz, $CDCl_3$) δ 4.49 (1H, p, *J* = 8.3 Hz), 3.35–3.29 (2H, m), 3.29–3.25 (2H, m), 2.70–2.61 (2H, m), 2.46–2.38 (2H, m), 1.72–1.63 (2H, m), 1.59–1.52 (2H, m), 1.44 (9H, s). Data in accordance with the literature.[4]

tert-Butyl 3-exo-Iodo-8-azabicyclo[3.2.1]octane-8-carboxylate (S7)

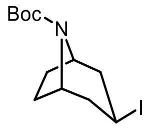

Following **GP1** *tert*-butyl *endo*-3-hydroxy-8-azabicyclo[3.2.1]octane-8-carboxylate (1.56 g, 6.93 mmol) gave **S7** as a solid (1.69 g, 73%). ^{1}H NMR (400 MHz, $CDCl_3$) δ 4.51 (1H, tt, *J* = 11.9, 5.7 H), 4.14–4.04 (1H, m), 4.04–3.92 (1H, m), 2.47–2.23(2H, m), 2.22–2.14 (2H, m), 1.98–1.83 (2H, m), 1.71–1.57 (2H, m), 1.47 (9H, s); ^{13}C NMR (101 MHz, $CDCl_3$, rotamers) δ 150.2, 81.2, 56.2, 55.6, 45.3, 44.5, 28.5, 27.7, 27.1, 18.5. Data in accordance to the literature.[5]

2-Iodo-2,3-dihydro-1*H*-indene (S8)

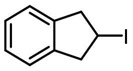

Following **GP1** 2,3-dihydro-1*H*-inden-2-ol (1.34 g, 10.0 mmol) gave **S8** as a solid (2.19 g, 90%). ^{1}H NMR (500 MHz, $CDCl_3$) δ 7.38–7.10 (4H, m), 4.76 (1H, tt, *J* = 6.2, 5.0 Hz), 3.51 (2H, dd, *J* = 16.8, 6.5 Hz), 3.34 (2H, dd, *J* = 16.8, 5.0 Hz). Data in accordance with the literature.[6]

4-Iodocyclohexan-1-one (S9)

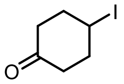

Following **GP1** 4-hydroxycyclohexan-1-one (570 mg, 5.0 mmol) gave **S9** as a solid (871 mg, 78%). ^{1}H NMR (400 MHz, $CDCl_3$) δ 4.73–4.68 (1H, m), 2.68–2.63 (2H, m), 2.40–2.25 (4H, m), 2.20–2.15(2H, m). Data in accordance with the literature.[7]

2-(4-Iodopiperidin-1-yl)pyrimidine (S10)

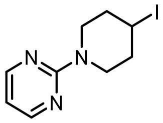

Following **GP1** 1-(pyrimidin-2-yl)piperidin-4-ol (3.56 g, 20.0 mmol) gave **S10** as a solid (5.19 g, 90%). ^{1}H NMR (400 MHz, $CDCl_3$) δ 8.30 (2H, d, *J* = 4.7 Hz), 6.48 (1H, t, *J* = 4.7 Hz), 4.55 (1H, tt, J= 6.9, 5.1 Hz), 4.13–4.04 (2H, m), 3.63 (2H, ddd, J= 13.7, 6.9, 4.7 Hz), 2.17–2.07 (4H, m). Data in accordance with the literature.[2]

***N*-Allyl-*N*-(2-iodoethyl)-4-methylbenzenesulfonamide (S11)**

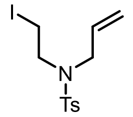

Following **GP1** *N*-allyl-*N*-(2-hydroxyethyl)-4-methylbenzenesulfonamide (2.55 g, 10.0 mmol) gave **S11** as a solid (3.00 g, 82%). ^{1}H NMR (400 MHz, $CDCl_3$) δ 7.69 (2H, d, *J* = 8.0 Hz), 7.31 (2H, d, *J* = 8.0 Hz), 5.70–5.63 (1H, m), 5.18 (2H, t, *J* = 8.8 Hz), 3.78 (2H, d, *J* = 6.4 Hz), 3.41 (2H, t, *J* = 7.7 Hz), 3.22 (2H, t, *J* = 8.5 Hz), 2.43 (3H, s). Data in accordance with the literature.[8]

7-(2-Iodopropyl)-1,3-dimethyl-3,7-dihydro-1*H*-purine-2,6-dione (S12)

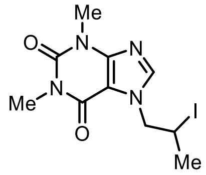

Following **GP1**, 7-(2-hydroxypropyl)-1,3-dimethyl-3,7-dihydro-1H-purine-2,6-dione (1.00 g, 4.2 mmol) gave **S12** as a solid (1.08 g, 74%). ^{1}H NMR (500 MHz, CDCl3) δ 7.65 (1H, s), 4.61–4.54 (1H, m), 4.50 (1H, dd, *J* = 14.1, 5.0 Hz), 4.37 (1H, dd, *J* = 14.1, 9.0 Hz), 3.60 (3H, s), 3.40 (3H, s), 1.96 (3H, d, *J* = 6.9 Hz); ^{13}C NMR (126 MHz, CDCl3) δ 155.4, 151.7, 149.4, 141.6, 106.7, 56.8, 30.0, 28.2, 25.4, 25.0. Data in accordance with the literature.[9]

***trans*-1-Ethoxy-2-iodocyclohexane (S13)**

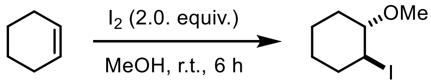

A round-bottom flask equipped with a stirring bar was charged with cyclohexene (1.23 g, 15.0 mmol, 1.0 equiv.) and MeOH (45 mL, 0.3 M). I_2 (7.61 g, 30.0 mmol, 2.0 equiv.) was slowly added to the vigorously stirred solution. After 6 h, the reaction mixture was diluted with Et_2O (50 mL) and $Na_2S_2O_3$ (30 mL, sat.). The layers were separated and the organic layer was washed with H_2O (2 x 30 mL), brine (30 mL) and dried ($MgSO_4$). The residue was then evaporated and purified by distillation to afford **S13** as an oil (2.85 g, 79%). ^{1}H NMR (500 MHz, $CDCl_3$) δ 4.05 (1H, ddd, *J* = 10.7, 8.7, 4.2 Hz), 3.39 (3H, s), 3.22 (1H, td, *J* = 9.0, 4.1 Hz), 2.41–2.33 (1H, m), 2.21–2.14 (1H, m), 2.02–1.89 (1H, m), 1.80 (1H, dtt, *J* = 11.6, 4.5, 1.8 Hz), 1.52 (1H, ddtt, *J* = 12.0, 5.1, 3.4, 1.8 Hz), 1.41–1.16 (3H, m). Data in accordance with the literature.[10]

trans-2-(Allyloxy)-3-iodotetrahydro-2_H_-pyran (S14)

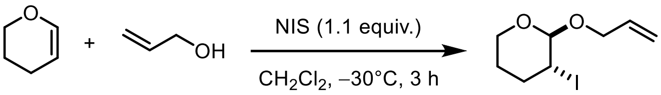

A round-bottom flask equipped with a stirring bar was charged with 3,4-dihydro-2*H*-pyran (315 mg, 3.75 mmol, 1.25 equiv.), allyl alcohol (174 mg, 3.0 mmol, 1.0 equiv.) and CH_2Cl_2 (6 mL, 0.5 M). The reaction was cooled to –30 °C and then N-iodosuccinimide (742 g, 3.3 mmol, 1.1 equiv.) was slowly added to the vigorously stirred solution. After 3 h, the mixture was diluted with Et_2O (50 mL) and $Na_2S_2O_3$ (30 mL, sat.). The layers were separated and the organic layer was washed with H_2O (2 x 30 mL), brine (30 mL), dried ($MgSO_4$) and evaporated. Purification by flash column chromatography on silica gel eluting hexane–EtOAc, gave **S14** as an oil (435 mg, 54%). ^{1}H NMR (400 MHz, $CDCl_3$) δ 6.00–5.86 (1H, m), 5.32 (1H, dq, *J* = 17.3, 1.7 Hz), 5.32 (1H, dq, *J* = 17.3, 1.7 Hz), 5.20 (1H, dq, *J* = 10.4, 1.4 Hz), 4.67 (1H, d, *J* = 5.3 Hz), 4.25 (1H, ddt, *J* = 12.9, 5.2, 1.5 Hz), 4.16–3.90 (3H, m), 3.58 (1H, ddd, *J* = 11.2, 7.4, 3.5 Hz), 2.46–2.28 (1H, m,), 2.10–1.95 (1H, m), 1.85–1.71 (1H, m), 1.66–1.50 (1H, m). Data in accordance with the literature.[11]

4-Iodo-1-oxaspiro[5.5]undecane (S15)

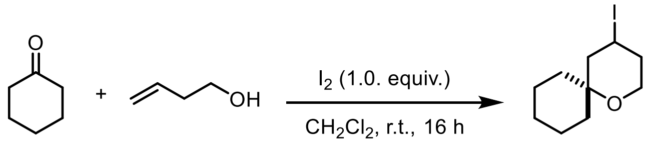

A round-bottom flask equipped with a stirring bar was charged with cyclohexanone (981 mg, 10.0 mmol, 1.0 equiv.), but-3-en-1-ol (1.44 g, 20.0 mmol, 2.0 equiv.) and CH_2Cl_2 (50 mL, 0.2 M). I_2 (2.53 g, 10.0 mmol, 1.0 equiv.) was slowly added to the vigorously stirred solution. After 6 h, the mixture was diluted with Et_2O (50 mL) and $Na_2S_2O_3$ (30 mL, sat.). The layers were separated and the organic layer was washed with H_2O (2 x 30 mL), brine (30 mL), dried ($MgSO_4$) and evaporated. Purification by flash column chromatography on silica gel eluting hexane–EtOAc, gave **S15** as an oil (1.17 g, 42%). ^{1}H NMR (500 MHz, $CDCl_3$) δ 4.48 (1H, tt, *J* = 12.5, 4.4 Hz), 3.67–3.54 (2H, m), 2.34–2.15 (2H, m), 2.06–1.92 (2H, m), 1.69–1.19 (10H, m). Data in accordance with the literature.[12]

2-(4-Bromophenyl)-4-iodotetrahydro-2*H*-pyran (S16)

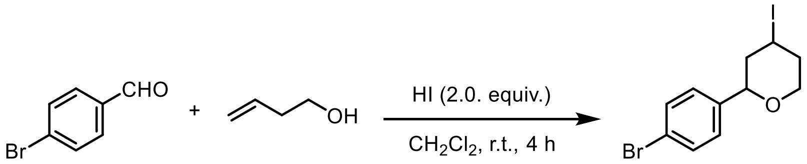

A round bottomed flask equipped with a stirring bar was charged with 4-bromobenzaldehyde (0.3 g, 1.6 mmol, 1.0 equiv.), CH_2Cl_2 (16 mL), but-3-en-1-ol (0.28 mL, 3.2 mmol, 2.0 equiv.) and HI (0.4 mL, 55 wt% solution in water, 3.2 mmol, 2.0 equiv.). The mixture was stirred at room temperature for 4 h when it was judged complete (TLC analysis). The mixture was diluted with H_2O (30 mL), the layers were separated, and the aqueous layer was extracted with CH_2Cl_2 (3 x 30 mL). The combined organic layers were washed with $Na_2S_2O_3$ (30 mL, 10% solution), brine (30 mL), dried ($MgSO_4$), filtered, and evaporated. Purification by flash column chromatography on silica gel eluting hexane–EtOAc, gave **S16** (0.29 g, 49%) as a solid. ^{1}H NMR (400 MHz, $CDCl_3$) δ 7.46 (2H, d, *J* = 8.5 Hz), 7.22 (2H, d, *J* = 8.5 Hz), 4.91 (1H, t, *J* = 3.3 Hz), 4.80 (1H, dd, *J* = 10.6, 2.1 Hz), 4.05 (2H, t, *J* = 5.6 Hz), 2.18 (1H, dt, *J* = 14.7, 2.7 Hz), 1.96 (2H, dt, *J* = 5.3, 2.7 Hz), 1.81 (1H, ddd, *J* = 14.5, 10.6, 3.5 Hz). Data in accordance with the literature.[13]

3 Preparation of the Oxidants

Preparation of cumOOTMS

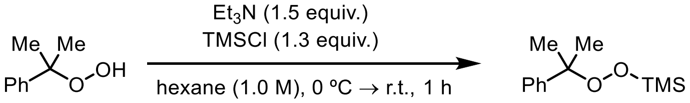

A round bottom flask equipped with a stirring bar was charged with Et_3N (10.5 mL, 75.0 mmol) and hexane (100 mL). Cumene hydroperoxide (9.3 mL, 80% technical solution, 50.0 mmol) was slowly added. The reaction media was then cooled in an ice-water bath and TMSCl (8.25 mL, 65.0 mmol) was slowly added. When the addition was completed, the ice-water bath was removed and the reaction was let stirring at room temperature. After 1 hour, H_2O (100 mL) was added. The layers were separated and the organic phase was washed with H_2O (100 mL x 4) and brine (100 mL), dried ($MgSO_4$), filtered and evaporated to give cumOOTMS as an oil (10.97 g, 98%) with approx density of 0.94 g/mL. ^1H NMR (400 MHz, $CDCl_3$) δ 7.45 (2H, d, J = 8.4 Hz), 7.33 (2H, t, J = 7.7 Hz), 7.24 (1H, t, J = 7.9 Hz), 1.58 (6H, s), 0.18 (9H, s); ^{13}C NMR (101 MHz, $CDCl_3$) δ 145.4, 127.9, 127.0, 125.6, 83.4, 26.5, –1.1. Data in accordance with the literature.[5]

Preparation of cumOOTES

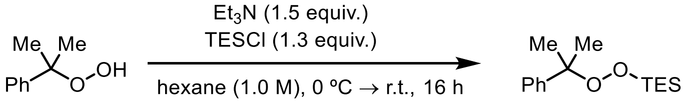

A round bottom flask equipped with a stirring bar was charged with Et_3N (10.5 mL, 75.0 mmol) and hexane (100 mL). Cumene hydroperoxide (9.3 mL, 80% technical solution, 50.0 mmol) was slowly added. The reaction media was then cooled in an ice-water bath and TESCl (10.9 mL, 65.0 mmol) was slowly added. When the addition was completed, the ice-water bath was removed and the reaction was let stirring at room temperature. After 16 hours, H_2O (100 mL) was added. The layers were separated and the organic phase was washed with H_2O (100 mL x 4) and brine (100 mL), dried ($MgSO_4$), filtered through a short pad of SiO_2 and evaporated to give cumOOTES as an oil (10.42 g, 78%) with approx density of 0.94 g/mL. ^1H NMR (400 MHz, $CDCl_3$) δ 7.48–7.42 (2H, m), 7.37–7.30 (2H, m), 7.27–7.21 (1H, m), 1.57 (6H, s), 0.98 (9H, t, J = 7.9 Hz), 0.69 (6H, q, J = 8.0 Hz); ^{13}C NMR (101 MHz, $CDCl_3$) δ 145.8, 127.9, 126.8, 125.5, 83.3, 26.5, 6.8, 3.9. Data in accordance with the literature.[14]

Preparation of cumOOSiPh$_2$*t*-Bu (S17)

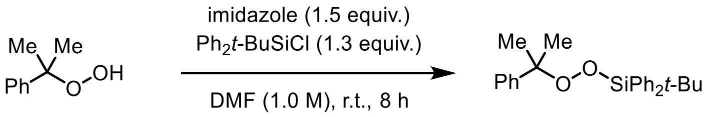

A round bottom flask equipped with a stirring bar was charged with imidazole (1.0 g, 15 mmol, 1.5 equiv.), cumene hydroperoxide (1.85 mL, 80% technical solution, 10.0 mmol, 1.00 equiv.) and DMF (10 mL). Ph$_2$*t*-BuSiCl (3.3 mL, 13 mmol, 1.3 equiv.) was slowly added at room temperature. After 8 hours, H_2O (80 mL) and Et_2O (120 mL) were added. The layers were separated and the organic phase was washed with H_2O (80 mL x 4) and brine (100 mL), dried ($MgSO_4$). Solvent was removed under reduced pressure and the residue was purified by flash column chromatography on silica gel eluting hexane–EtOAc to give cumOOSiPh$_2$*t*-Bu **S17** as an oil (1.6 g, 40%). ^{1}H NMR (400 MHz, $CDCl_3$) δ 8.01–7.91 (4H, m), 7.59 (8H, m), 7.51–7.38 (3H, m), 1.77 (6H, s), 1.35 (9H, s); ^{13}C NMR (101 MHz, $CDCl_3$) δ 145.5, 136.1, 133.3, 129.9, 128.0, 127.6, 127.0, 125.8, 84.5, 27.5, 26.6, 19.7; HRMS (ESI) found: MNa^+ 413.1896, $C_{25}H_{30}O_2SiNa$ requires 413.1907.

Stability studies of cumOOTMS

CumOOTMS does not have explosion propagating property and can be used on scale as we have discussed previously.[5]

4 Preparation of Boronate Complexes

General Procedure for the Boronate Preparation – GP2

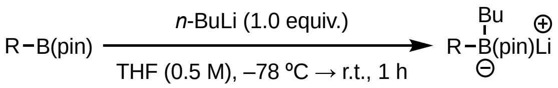

An oven-dry tube equipped with a stirring bar was charged with the boronic ester (1.1 equiv.). The tube was capped with a Supelco aluminium crimp seal with septum (PTFE/butyl), evacuated and refilled with N_2 (x 3). Dry and degassed THF (0.5 M) was added and the tube was cooled to –78 ºC. *n*-BuLi (1.0 equiv., 2.5 M in hexanes) was added dropwise and after 1 minute the cooling bath was removed and the stirring was continued for 1 h. The THF solution of the boronate was directly used in the coupling reactions.

The structures of all boronates prepared are shown in **Scheme S1.** All boronic acid pinacol esters used for the preparation of boronates complexes are commercially available.

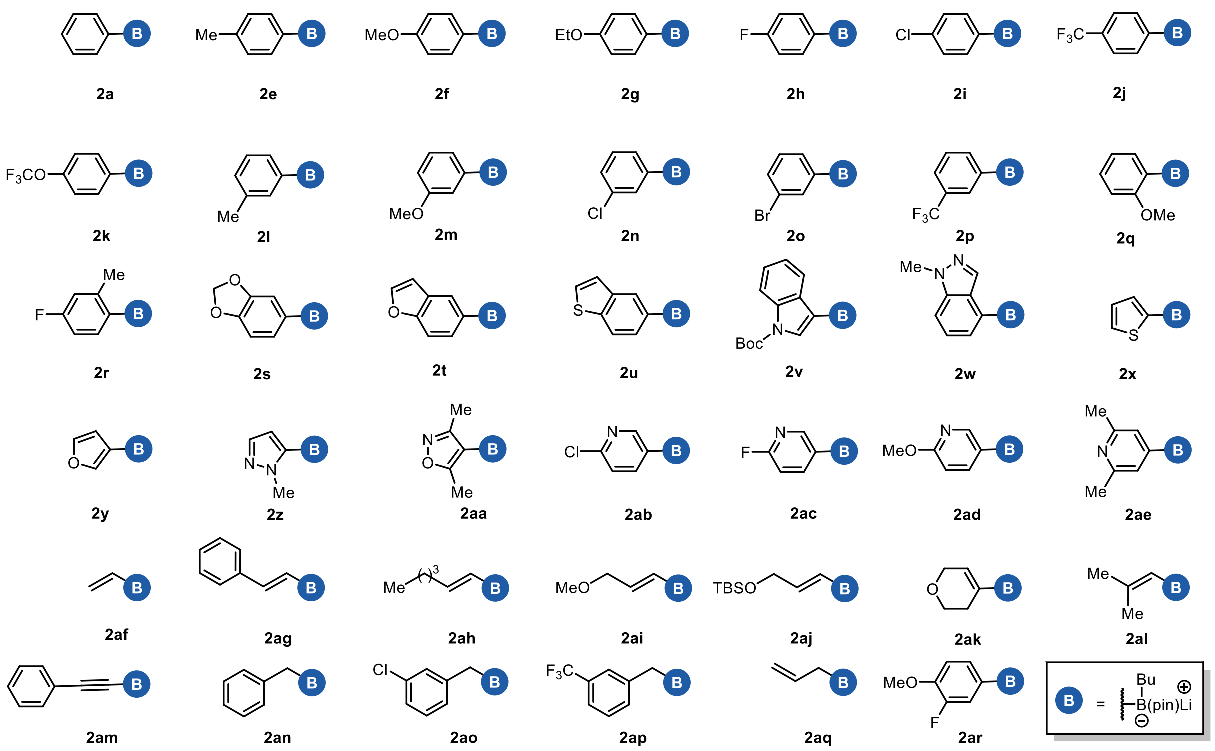

Scheme S1.

5 Reaction Optimization

General Procedure for the Reaction Optimization – GP3

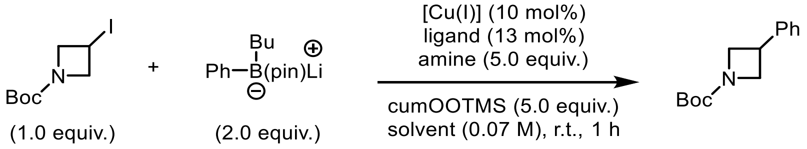

An oven-dry tube equipped with a stirring bar was charged with the [Cu(I)] catalyst (0.010 mmol, 10 mol%) and the ligand (0.013 mmol, 13 mol%). The tube was capped with a Supelco aluminium crimp seal with septum (PTFE/butyl), evacuated and refilled with N_2 (x 3). Dry and degassed solvents (1.0 mL), *tert*-butyl 3-iodoazetidine-1-carboxylate (28 mg, 0.10 mmol, 1.0 equiv.), the amine (0.50 mmol, 5.0 equiv.), boronate **2a** (0.50 mL, 0.5 M in THF, 0.25 mmol, 2.5 equiv.) were sequentially addled. Finally, the oxidant (0.50 mmol, 5.0 equiv.) was added dropwise keeping the reaction mixture under vigorous stirring. After 1 h, NH_4Cl sat. (1 mL) was added and the mixture was stirred for 3 min. EtOAc (20 mL) and a solution of 1,3-dinitrobenzene (internal standard) in EtOAc (1 mL, 0.05 M, 0.5 equiv.) were added. The mixture was washed with water (20 mL), brine (20 mL), dried ($MgSO_4$), filtered, and evaporated. The yield was determined by ^{1}H NMR using 1,3-dinitrobenzene as the internal standard.

Table S1.

Entry	[Cu(I)]	Ligand	Amine	Solvent	Yield (%)
**1**	$[Cu(CH_3CN)_4]\ PF_6$	TMHD	Et_3N	DMSO	55
**2**		RuPhos			25
**3**		TMEDA			21
**4**		Xantphos			40
**5**		1,10-phenanthroline			20
**6**		BINOL			29
**7**		2-pyridinecarboxylic acid			41
**8**		terpyridine			50
**10**		1,3-diphenyl-1,3-propanedione			26
**11**		biquinoline			30
**12**		dtbbpy			14
**13**	CuI	TMHD			39
**14**	CuTC				44
**15**	$(CuOTf)_2PhCH_3$				33
**16**	CuCN				33
**17**	CuOAc				12

**Entry**	**[Cu]**	**Ligand**	**Amine**	**Solvent**	**Yield (%)**
**18**	$[Cu(CH_3CN)_4]\ PF_6$	TMHD	*n*-Bu_3N	DMSO	31
**19**			*i*-Pr_3N		47
**20**			PMP		42
**21**			*i*-Pr_2NMe		38
**22**			Et_3N	NMP	13
**23**				DMA	20
**24**				DMPU	5
**25**				CH_3CN	17
**26**				EtOAc	0
**27**				THF	0
**28**				dioxane	0
**29**				glyme	0
**30**				CPME	0

Since no significant further improvements were obtained we focused on evaluating the ratio of reagents used in Table S1 entry 1 (Table S2).

Table S2.

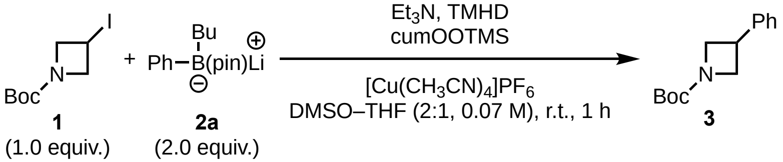

Entry	2a (equiv.)	Et_3N (equiv.)	cumOOTMS (equiv.)	TMHD (mol%)	$[Cu(CH_3CN)_4]PF_6$ (mol%)	Yield (%)
**1**	2.0	5	5	13	10	55
**2**	2.0	5	4	13	10	54
**3**	2.0	5	3	13	10	58
**4**	2.0	5	2	13	10	59
**5**	2.0	5	1.5	13	10	40
**6**	2.0	4	2	13	10	60
**7**	2.0	3	2	13	10	56
**8**	2.0	2	2	13	10	57
**9**	2.0	2	3	13	10	62
**10**	2.0	2	4	13	10	55
**11**	2.0	2	5	13	10	50
**12**	2.0	3	3	13	10	64
**13**	2.5	3	3	25	20	72

6 Control Experiments

Control experiments were set up based on the optimised reaction conditions (**Scheme S2**).

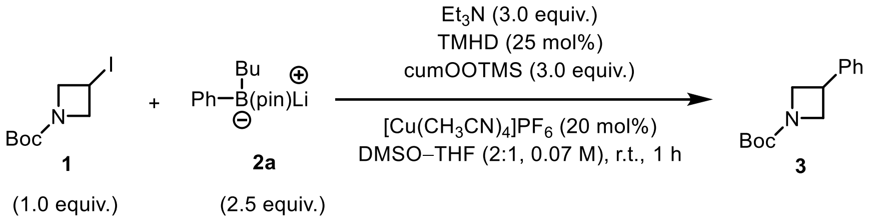

Scheme S2.

Table S3.

Entry	Deviation from the reaction conditions	Yield (%)	1 (%)
**1**	none	72	–
**2**	no Et_3N	16	80
**3**	no $[Cu(CH_3CN)_4]PF_6$	–	100
**4**	no TMHD	37	–
**5**	no cumOOTMS	–	100

Reaction in the absence of Et_3N provided **3**, but in lower yield (**Table S3**, entry 3). Upon analysis of the reaction crude by GC-MS we identified the formation of Ph–I and acetophenone (**Scheme S3**). We believe that **2a** might eventually be oxidised by cumOOTMS to form either a Ph• or a Bu•, both of which can activate **1** by XAT and thus lead to product formation. However, Bu–I was not detected in the reaction mixture.

An alternative mechanistic pathway that could lead to product formation in the presence of the amine, would involve the fragmentation of the cumO• to form Me• that has been demonstrated as a XAT mediator. Furthermore, we have been able to detect benzophenone in the crude mixture.

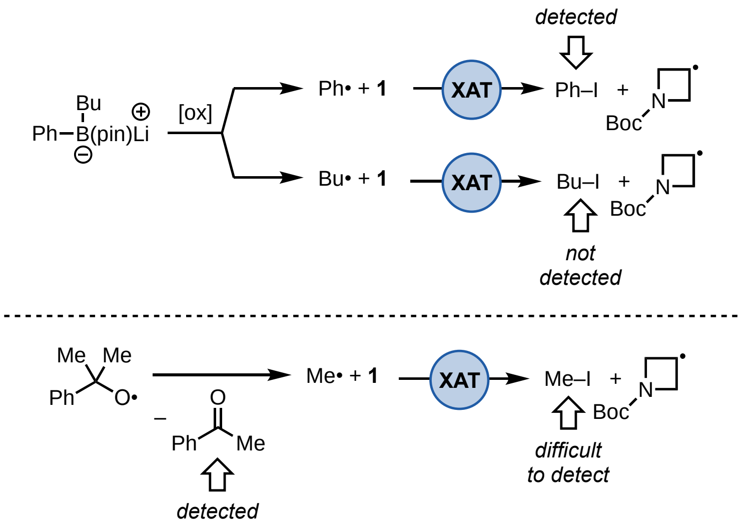
detected
Bu
Ph–B(pin)Li
[ox]
Ph• + 1
XAT
Ph–I +
N
Boc
Bu• + 1
XAT
Bu–I +
N
Boc
not
detected
Me Me
Ph
O•
Me• + 1
XAT
Me–I +
N
Boc
O
Ph
Me
detected
difficult
to detect

Scheme S3.

7 Substrate Scope

General Procedure for the C-C Coupling Reaction – GP4

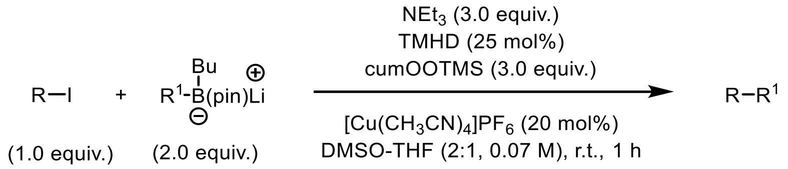

An oven-dry tube equipped with a stirring bar was charged with the alkyl iodide (0.10 mmol, 1.0 equiv) and $[Cu(MeCN)_4]PF_6$ (7.5 mg, 0.020 mmol, 20 mol%). The tube was capped with a Supelco aluminium crimp seal with septum (PTFE/butyl), evacuated and refilled with N_2 (x 3). Dry and degassed DMSO (1.0 mL), TMHD (5.2 µL, 0.025 mmol, 25 mol%), NEt_3 (42 µL, 0.30 mmol, 3.0 equiv.) and the boronate (0.5 mL, 0.25 mmol, 2.5 equiv., 0.5M in THF) were sequentially added. cumOOTMS (67 mg, 72 µL, 0.30 mmol, 3.0 equiv.) was added dropwise to the vigorously stirred solution. Stirring was continued for 1 h, then NH_4Cl sat. (20 mL), EtOAc (20 mL) were added. The organic layer was separated and the aqueous layer was extracted with EtOAc (15 mL x 2). The combined organic phases were washed with water (20 mL), brine (20 mL), dried ($MgSO_4$), filtered, and evaporated. The crude mixture was purified by flash column chromatography on silica gel.

***tert*-Butyl 3-Phenylazetidine-1-carboxylate (3)**

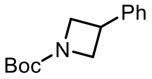

Following **GP4**, 1-Boc-3-iodoazetidine (19 µL, 0.10 mmol) and boronate **2a** (0.50 mL, 0.50 M in THF, 0.25 mmol) gave **3** (17 mg, 72%) as an oil. ^{1}H NMR (400 MHz, $CDCl_3$) δ 7.36–7.19 (5H, m), 4.31 (2H, t, *J* = 8.7 Hz), 3.97 (2H, dd, *J* = 8.6, 6.1 Hz), 3.71 (1H, m), 1.46 (9H, s). Data in accordance with the literature.[15]

***tert*-Butyl 3-(*p*-Tolyl)azetidine-1-carboxylate (6)**

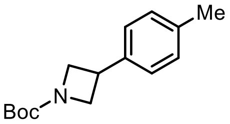

Following **GP4**, 1-Boc-3-iodoazetidine (19 µL, 0.10 mmol), boronate **2e** (0.50 mL, 0.50 M in THF, 0.25 mmol) gave **6** (18 mg, 73%) as an oil. ^{1}H NMR (400 MHz, CDCl3) δ 7.22–7.14 (4H, m), 4.31 (2H, t, *J* = 8.7 Hz), 3.98–3.94 (2H, m), 3.73–3.67 (1H, m), 2.34 (3H, s), 1.47 (9H, s). Data in accordance with the literature.[16]

***tert*-Butyl 3-(4-Methoxyphenyl)azetidine-1-carboxylate (7)**

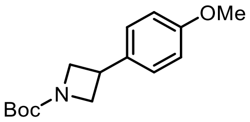

Following **GP4**, 1-Boc-3-iodoazetidine (19 µL, 0.10 mmol), boronate **2f** (0.50 mL, 0.50 M in THF, 0.25 mmol) gave **7** (21 mg, 78%) as an oil. ^{1}H NMR (400 MHz, $CDCl_3$) δ 7.24 (2H, dd, *J* = 8.5, 2.1 Hz), 6.88 (2H, d, *J* = 8.6 Hz), 4.30 (2H, t, *J* = 8.6 Hz), 3.93 (2H, dd, *J* = 8.6, 6.0 Hz), 3.80 (3H, s), 3.72–3.62 (1H, m), 1.46 (9H, s). Data in accordance with the literature.[17]

***tert*-Butyl 3-(4-Ethoxyphenyl)azetidine-1-carboxylate (8)**

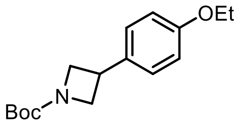

Following **GP4**, 1-Boc-3-iodoazetidine (19 µL, 0.10 mmol), boronate **2g** (0.50 mL, 0.50 M in THF, 0.25 mmol) gave **8** (22 mg, 81%) as an oil. ^{1}H NMR (400 MHz, $CDCl_3$) δ 7.24–7.18 (2H, m), 6.90–6.85 (2H, m), 4.30 (2H, t, *J* = 8.6 Hz), 4.03 (2H, q, *J* = 7.0 Hz), 3.93 (2H, dd, *J* = 8.5, 6.1 Hz), 3.67 (1H, tt, *J* = 8.7, 6.1 Hz), 1.46 (9H, s), 1.41 (3H, t, *J* = 7.0 Hz); ^{13}C NMR (101 MHz, $CDCl_3$) δ 158.1, 156.6, 134.3, 127.9, 114.8, 79.6, 63.6, 56.7, 33.0, 28.6, 15.0; HRMS (ESI) found: MNa^+ 300.1570, $C_{16}H_{23}O_3NNa$ requires 300.1570.

***tert*-Butyl 3-(4-Fluorophenyl)azetidine-1-carboxylate (9)**

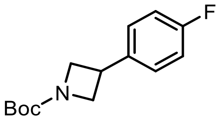

Following **GP4**, 1-Boc-3-iodoazetidine (19 µL, 0.10 mmol), boronate **2h** (0.50 mL, 0.50 M in THF, 0.25 mmol) gave **9** (16 mg, 63%) as an oil. ^{1}H NMR (400 MHz, $CDCl_3$) δ 7.32–7.26 (2H, m), 7.07–7.01 (2H, m), 4.32 (2H, t, *J* = 8.7 Hz), 3.96–3.90 (2H, m), 3.74–3.63 (1H, m), 1.47 (9H, s). Data in accordance with the literature.[16]

***tert*-Butyl 3-(4-Chlorophenyl)azetidine-1-carboxylate (10)**

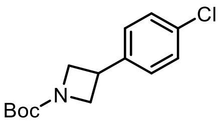

Following **GP4**, 1-Boc-3-iodoazetidine (19 µL, 0.10 mmol), boronate **2i** (0.50 mL, 0.50 M in THF, 0.25 mmol) gave **10** (16 mg, 58%) as an oil. ^{1}H NMR (400 MHz, $CDCl_3$) 7.30 (2H, d, *J*

= 8.5 Hz), 7.23 (2H, d, *J* = 8.5 Hz), 4.31 (2H, t, *J* = 8.7 Hz), 3.92 (2H, dd, *J* = 8.7, 6.0 Hz), 3.73–3.65 (1H, m), 1.45 (9H, s). Data in accordance with the literature.[18]

***tert*-Butyl 3-(4-(Trifluoromethyl)phenyl)azetidine-1-carboxylate (11)**

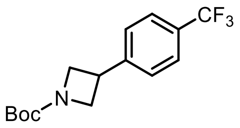

Following **GP4**, 1-Boc-3-iodoazetidine (19 µL, 0.10 mmol), boronate **2j** (0.50 mL, 0.50 M in THF, 0.25 mmol) gave **11** (13 mg, 44%) as an oil. ^{1}H NMR (500 MHz, $CDCl_3$) *δ* ppm: 7.60 (2H, d, *J* = 8.0 Hz,), 7.43 (2H, d, *J* = 8.1 Hz), 4.36 (2 H, t, *J* = 8.7 Hz), 3.97 (2H, m), 3.82–3.76 (1H, m), 1.47 (9H, s). Data in accordance with the literature.[16]

***tert*-Butyl 3-(4-(Trifluoromethoxy)phenyl)azetidine-1-carboxylate (12)**

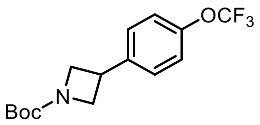

Following **GP4**, 1-Boc-3-iodoazetidine (19 µL, 0.10 mmol), boronate **2k** (0.50 mL, 0.50 M in THF, 0.25 mmol) gave **12** (19 mg, 61%) as an oil. ^{1}H NMR (400 MHz, $CDCl_3$) δ 7.34–7.32 (2H, m), 7.21–7.18 (2H, m), 4.34 (2H, t, *J* = 8.7 Hz), 3.96–3.90 (2H, m), 3.77–3.68 (1H, m), 1.47 (9H, s). Data in accordance with the literature.[18]

***tert*-Butyl 3-(*m*-Tolyl)azetidine-1-carboxylate (13)**

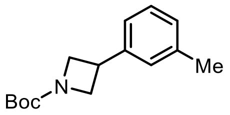

Following **GP4**, 1-Boc-3-iodoazetidine (19 µL, 0.10 mmol), boronate **2l** (0.50 mL, 0.50 M in THF, 0.25 mmol) gave **13** (15 mg, 60%) as an oil. ^{1}H NMR (400 MHz, $CDCl_3$) *δ* ppm: 7.25–7.21 (1H, m), 7.14–7.05 (3H, m), 4.31 (2H, t, *J* = 8.6 Hz), 4.02–3.93 (2H, m), 3.74–3.68 (1H, m), 2.36 (3H, s), 1.47 (9H, s). Data in accordance with the literature.[16]

***tert*-Butyl 3-(3-Methoxyphenyl)azetidine-1-carboxylate (14)**

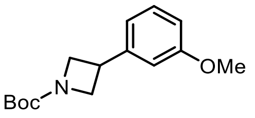

Following **GP4**, 1-Boc-3-iodoazetidine (19 µL, 0.10 mmol), boronate **2m** (0.50 mL, 0.50 M in THF, 0.25 mmol) gave **14** (19 mg, 74%) as an oil. ^{1}H NMR (400 MHz, $CDCl_3$) *δ* ppm: 7.28–

7.23 (1H, m), 6.91–6.77 (3H, m), 4.31 (2H, t, *J* = 8.6 Hz), 4.00–3.94 (2H, m), 3.81 (3H, s), 3.75–3.66 (1H, m), 1.47 (9H, s). Data in accordance with the literature.[16]

***tert*-Butyl 3-(3-Chlorophenyl)azetidine-1-carboxylate (15)**

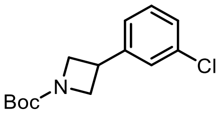

Following **GP4**, 1-Boc-3-iodoazetidine (19 µL, 0.10 mmol), boronate **2n** (0.50 mL, 0.50 M in THF, 0.25 mmol) gave **15** (15 mg, 57%) as an oil. ^{1}H NMR (400 MHz, $CDCl_3$) *δ* ppm: 7.33–7.14 (4H, m), 4.32 (2H, t, *J* = 8.7 Hz), 3.99–3.89 (2H, m), 3.74–3.65 (1H, m), 1.47 (9H, s). Data in accordance with the literature.[16]

***tert*-Butyl 3-(3-Bromophenyl)azetidine-1-carboxylate (16)**

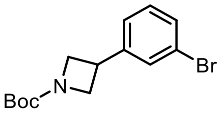

Following **GP4**, 1-Boc-3-iodoazetidine (19 µL, 0.10 mmol), boronate **2o** (0.50 mL, 0.50 M in THF, 0.25 mmol) gave **16** (17 mg, 56%) as an oil. ^{1}H NMR (500 MHz, $CDCl_3$) δ 7.46 (1H, t, *J* = 1.7 Hz), 7.39 (1H, dt, *J* = 7.1, 1.9 Hz), 7.26–7.20 (2H, m), 4.32 (2H, t, *J* = 8.7 Hz), 3.94 (2H, dd, *J* = 8.6, 5.9 Hz), 3.74–3.62 (1H, m), 1.47 (9H, s). ^{13}C NMR (126 MHz, $CDCl_3$) δ 156.5, 144.8, 130.4, 130.3, 130.1, 125.6, 123.0, 79.9, 56.5, 33.3, 28.6; HRMS (ESI) found: MNa^+ 334.0405, $C_{14}H_{18}O_2NBrNa$ requires 334.0400.

***tert*-Butyl 3-(3-(Trifluoromethyl)phenyl)azetidine-1-carboxylate (17)**

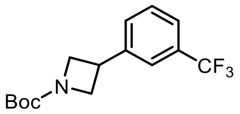

Following **GP4**, 1-Boc-3-iodoazetidine (19 µL, 0.10 mmol), boronate **2p** (0.50 mL, 0.50 M in THF, 0.25 mmol) gave **17** (16 mg, 54%) as an oil. ^{1}H NMR (400 MHz, $CDCl_3$) *δ* ppm: 7.55–7.45 (4H, m), 4.36 (2H, t, *J* = 8.8 Hz), 3.99–3.93 (2H, m), 3.81–3.74 (1H, m), 1.47 (9H, s). Data in accordance with the literature.[18]

tert-Butyl 3-(2-Methoxyphenyl)azetidine-1-carboxylate (18)

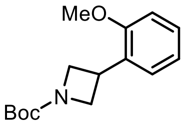

Following **GP4**, 1-Boc-3-iodoazetidine (19 µL, 0.10 mmol), boronate **2q** (0.50 mL, 0.50 M in THF, 0.25 mmol) gave **18** (18 mg, 70%) as an oil. ^{1}H NMR (400 MHz, $CDCl_3$) δ 7.26–7.20 (2H, m), 7.00–6.90 (1H, m), 6.88–6.83 (1H, m), 4.26 (2H, t, *J* = 8.3 Hz), 4.12–3.90 (3H, m), 3.81 (3H, s), 1.46 (9H, s). Data in accordance with the literature.[19]

tert-Butyl 3-(4-Fluoro-2-methylphenyl)azetidine-1-carboxylate (19)

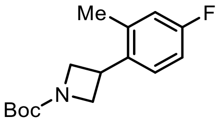

Following **GP4**, 1-Boc-3-iodoazetidine (19 µL, 0.10 mmol), boronate **2r** (0.50 mL, 0.50 M in THF, 0.25 mmol) gave **19** (18 mg, 68%) as an oil. ^{1}H NMR (500 MHz, $CDCl_3$) δ 7.29 (1H, dd, *J* = 8.6, 5.7 Hz), 6.92 (1H, td, *J* = 8.4, 2.8 Hz), 6.87 (1H, dd, *J* = 9.6, 2.8 Hz), 4.30 (2H, t, *J* = 8.4 Hz), 3.98 (2H, t, *J* = 7.5 Hz), 3.93–3.83 (1H, m), 2.20 (3H, s), 1.46 (9H, s); ^{13}C NMR (126 MHz, $CDCl_3$) δ 161.6 (d, *J* = 244.8 Hz), 156.6, 138.3 (d, *J* = 7.7 Hz), 135.3 (d, *J* = 3.0 Hz), 127.0 (d, *J* = 8.3 Hz), 117.2 (d, *J* = 21.0 Hz), 112.95 (d, *J* = 21.0 Hz), 79.7, 55.1, 30.3, 28.5, 19.7 (d, *J* = 1.7 Hz); ^{19}F NMR (376 MHz, $CDCl_3$) δ –116.8; HRMS (ESI) found: MNa^+ 288.1362, $C_{15}H_{20}O_2NFNa$ requires 288.1370.

tert-Butyl 3-(Benzo[d][1,3]dioxol-5-yl)azetidine-1-carboxylate (20)

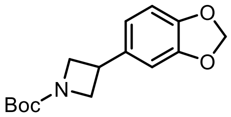

Following **GP4**, 1-Boc-3-iodoazetidine (19 µL, 0.10 mmol), boronate **2s** (0.50 mL, 0.50 M in THF, 0.25 mmol) gave **20** (19 mg, 68%) as an oil. ^{1}H NMR (500 MHz, $CDCl_3$) δ 6.84 (1H, d, *J* = 1.5 Hz), 6.79–6.70 (2H, m), 5.95 (2H, s), 4.29 (2H, t, *J* = 8.7 Hz), 3.92–3.88 (2H, m), 3.68–3.59 (1H, m), 1.46 (9H, s). Data in accordance with the literature.[20]

tert-Butyl 3-(Benzofuran-5-yl)azetidine-1-carboxylate (21)

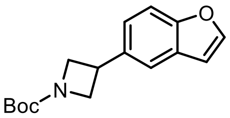

Following **GP4**, 1-Boc-3-iodoazetidine (19 µL, 0.10 mmol), boronate **2t** (0.50 mL, 0.50 M in THF, 0.25 mmol) gave **21** (20 mg, 75%) as an oil. ^{1}H NMR (400 MHz, $CDCl_3$) δ 7.63 (1H, d, *J* = 2.1 Hz), 7.56–7.52 (1H, m), 7.48 (1H, d, *J* = 8.5 Hz), 7.25 (1H, d, *J* = 7.6 Hz), 6.77–6.72 (1H, m), 4.37 (2H, t, *J* = 8.6 Hz), 4.01 (2H, dd, *J* = 8.5, 6.1 Hz), 3.82 (1H, tt, *J* = 8.7, 6.1 Hz), 1.48 (9H, s); ^{13}C NMR (101 MHz, $CDCl_3$) δ 156.6, 154.2, 145.7, 137.0, 127.9, 123.3, 119.3, 111.7, 106.6, 79.7, 57.3, 33.7, 28.6; HRMS (ESI) found: MNa^+ 296.1243, $C_{16}H_{19}NO_3Na$ requires 296.1263.

tert-Butyl 3-(Benzo[b]thiophen-5-yl)azetidine-1-carboxylat (22)

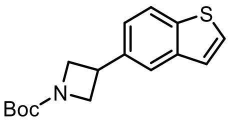

Following **GP4**, 1-Boc-3-iodoazetidine (19 µL, 0.10 mmol), boronate **2u** (0.50 mL, 0.50 M in THF, 0.25 mmol) gave **22** (17 mg, 60%) as an oil. ^{1}H NMR (400 MHz, $CDCl_3$) δ 7.86 (1H, d, *J* = 8.4 Hz), 7.76 (1H, d, *J* = 1.8 Hz), 7.46 (1H, d, *J* = 5.4 Hz), 7.35–7.29 (2H, m), 4.38 (2H, t, *J* = 8.7 Hz), 4.03 (2H, dd, *J* = 8.6, 6.0 Hz), 3.85 (1H, br. s), 1.48 (9H, s). Data in accordance with the literature.[18]

tert-Butyl 3-(1-(_tert_-Butoxycarbonyl)azetidin-3-yl)-1H-indole-1-carboxylate (23)

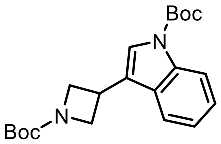

Following **GP4**, 1-Boc-3-iodoazetidine (19 µL, 0.10 mmol), boronate **2v** (0.50 mL, 0.50 M in THF, 0.25 mmol) gave **23** (21 mg, 56%) as an oil. ^{1}H NMR (500 MHz, $CDCl_3$) δ 8.15 (1H, d, *J* = 8.2 Hz), 7.53–7.45 (2H, m), 7.34 (1H, ddd, *J* = 8.4, 7.2, 1.2 Hz), 7.26–7.22 (1H, m), 4.37 (2H, t, *J* = 8.6 Hz), 4.11 (2H, dd, *J* = 8.5, 6.1 Hz), 3.95–3.87 (1H, m), 1.67 (9H, s), 1.47 (9H, s); ^{13}C NMR (126 MHz, $CDCl_3$) δ 156.6, 149.8, 136.0, 129.3, 124.9, 122.8, 122.4, 121.4, 119.1, 115.7, 83.9, 79.8, 55.1, 28.6, 28.4, 25.6; HRMS (ESI) found: MNa^+ 395.1931, $C_{21}H_{28}O_4N_2Na$ requires 395.1941.

***tert*-Butyl 3-(1-Methyl-1H-indazol-4-yl)azetidine-1-carboxylate (24)**

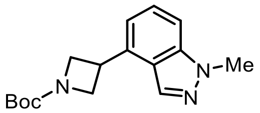

Following **GP4**, 1-Boc-3-iodoazetidine (19 µL, 0.10 mmol), boronate **2w** (0.50 mL, 0.50 M in THF, 0.25 mmol) gave **24** (16 mg, 56%) as an oil. ^{1}H NMR (500 MHz, $CDCl_3$) δ 8.02 (1H, s), 7.36 (1H, dd, *J* = 8.5, 6.8 Hz), 7.31 (1H, d, *J* = 8.4 Hz), 7.05 (1H, dt, *J* = 6.9, 0.9 Hz), 4.43 (2H, t, *J* = 8.7 Hz), 4.21 (2H, dd, *J* = 8.4, 6.0 Hz), 4.11-4.04 (1H, m), 4.09 (3H, s), 1.48 (9H, s); ^{13}C NMR (126 MHz, $CDCl_3$) δ 156.6, 140.4, 135.5, 131.0, 126.5, 122.5, 118.3, 108.1, 79.8, 55.6, 35.9, 32.3, 28.6; HRMS (ESI) found: MH^+ 288.1703, $C_{16}H_{22}O_2N_3$ requires 288.1707.

***tert*-Butyl 3-(Thiophen-2-yl)azetidine-1-carboxylate (25)**

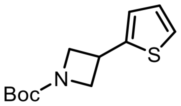

Following **GP4**, 1-Boc-3-iodoazetidine (19 µL, 0.10 mmol), boronate **2x** (0.50 mL, 0.50 M in THF, 0.25 mmol) gave **25** (17 mg, 70%) as an oil. ^{1}H NMR (400 MHz, $CDCl_3$) *δ* ppm: 7.22–7.18 (1H, m), 6.97–6.92 (2H, m), 4.40–4.28 (2H, m), 4.03–3.95 (3H, m), 1.46 (9H, s). Data in accordance with the literature.[21]

***tert*-Butyl 3-(Furan-3-yl)azetidine-1-carboxylate (26)**

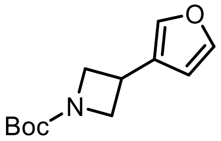

Following **GP4**, 1-Boc-3-iodoazetidine (19 µL, 0.10 mmol), boronate **2y** (0.50 mL, 0.50 M in THF, 0.25 mmol) gave **26** (11 mg, 50%) as an oil. ^{1}H NMR (400 MHz, $CDCl_3$) δ 7.40 (1H, m), 7.31 (1H, m), 6.44–6.39 (1H, m), 4.24 (2H, t, *J* = 8.5 Hz), 3.86 (2H, dd, *J* = 8.4, 6.0 Hz), 3.66–3.62 (1H, m), 1.45 (9H, s). Data in accordance with the literature.[22]

***tert*-Butyl 3-(1-Methyl-1H-pyrazol-4-yl)azetidine-1-carboxylate (27)**

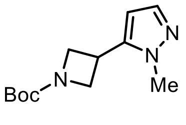

Following **GP4**, 1-Boc-3-iodoazetidine (19 µL, 0.10 mmol), boronate **2z** (0.50 mL, 0.50 M in THF, 0.25 mmol) gave **27** (16 mg, 68%) as an oil. ^{1}H NMR (400 MHz, $CDCl_3$) δ 7.41 (1H, s), 7.28 (1H, s), 4.26 (2H, t, *J* = 8.5 Hz), 3.88 (3H, s), 3.86–3.80 (2H, m), 3.71–3.57 (1H, m), 1.45

(9H, s); ^{13}C NMR (101 MHz, $CDCl_3$) δ 156.4, 137.6, 127.7, 123.2, 79.5, 57.0, 38.9, 28.4, 24.3; HRMS (ESI): Found MNa^+ 260.1362, $C_{12}H_{19}O_2N_3Na$ requires 260.1369.

***tert*-Butyl 3-(3,5-Dimethylisoxazol-4-yl)azetidine-1-carboxylate (28)**

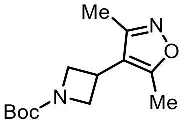

Following **GP4**, 1-Boc-3-iodoazetidine (19 µL, 0.10 mmol), boronate **2aa** (0.50 mL, 0.50 M in THF, 0.25 mmol) gave **28** (17 mg, 66%) as an oil. ^{1}H NMR (500 MHz, $CDCl_3$) δ 4.27 (2H, t, *J* = 8.9 Hz), 3.96 (2H, dd, *J* = 8.7, 6.2 Hz), 3.55 (1H, tt, *J* = 9.0, 6.2 Hz), 2.37 (3H, s), 2.30 (3H, s), 1.46 (9H, s); ^{13}C NMR (126 MHz, $CDCl_3$) δ 165.3, 158.9, 156.3, 113.7, 80.0, 54.9, 28.5, 23.1, 11.5, 10.8; HRMS (ESI) found: MNa^+ 275.1364, $C_{13}H_{20}O_3N_2Na$ requires 275.1366.

***tert*-Butyl 3-(6-Chloropyridin-3-yl)azetidine-1-carboxylate (29)**

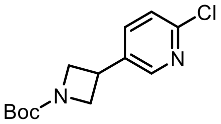

Following **GP4**, 1-Boc-3-iodoazetidine (19 µL, 0.10 mmol), boronate **2ab** (0.50 mL, 0.50 M in THF, 0.25 mmol) gave **29** (12 mg, 45%) as an oil. ^{1}H NMR (400 MHz, $CDCl_3$) δ 8.28 (1H, d, *J* = 2.5 Hz), 7.70 (1H, dd, *J* = 8.3, 2.6 Hz), 7.34 (1H, d, *J* = 8.3 Hz), 4.36 (2H, t, *J* = 8.7 Hz), 3.95–3.88 (2H, m), 3.72 (1H, m), 1.46 (9H, s). Data in accordance with the literature.[22]

***tert*-Butyl 3-(6-Fluoropyridin-3-yl)azetidine-1-carboxylate (30)**

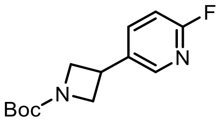

Following **GP4**, 1-Boc-3-iodoazetidine (19 µL, 0.10 mmol), boronate **2ac** (0.50 mL, 0.50 M in THF, 0.25 mmol) gave **30** (13 mg, 50%) as an oil. ^{1}H NMR (400 MHz, $CDCl_3$) δ 8.11 (1H, br s), 7.84–7.81 (1H, m), 6.99–6.93 (1H, m), 4.37 (2H, t, *J* = 8.6 Hz), 3.95–3.88 (2H, m), 3.77–3.73 (1H, m), 1.46 (9H, s). Data in accordance with the literature.[22]

tert-Butyl 3-(6-Methoxypyridin-3-yl)azetidine-1-carboxylate (31)

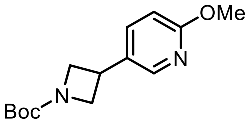

Following **GP4**, 1-Boc-3-iodoazetidine (19 µL, 0.10 mmol), boronate **2ad** (0.50 mL, 0.50 M in THF, 0.25 mmol) gave **31** (18 mg, 68%). ^{1}H NMR (400 MHz, CD_2Cl_2) δ 8.02 (1H, d, *J* = 2.5 Hz), 7.63 (1H, dd, *J* = 8.6, 2.6 Hz), 6.75 (1H, d, *J* = 8.6 Hz), 4.28 (2H, t, *J* = 8.6 Hz), 3.94–3.82 (5H, m), 3.68 (1H, tt, *J* = 8.7, 6.0 Hz), 1.43 (9H, s). Data in accordance with the literature.[22]

tert-Butyl 3-(2,6-Dimethylpyridin-4-yl)azetidine-1-carboxylate (32)

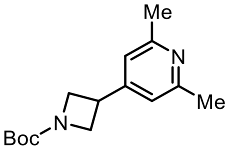

Following **GP4**, 1-Boc-3-iodoazetidine (19 µL, 0.10 mmol), boronate **2ae** (0.50 mL, 0.50 M in THF, 0.25 mmol) gave **32** (12 mg, 45%). ^{1}H NMR (500 MHz, $CDCl_3$) δ 6.94 (2H, s), 4.32 (2H, t, *J* = 8.7 Hz), 3.94 (2H, dd, *J* = 8.6, 5.8 Hz), 3.63 (1H, tt, *J* = 8.7, 5.8 Hz), 2.56 (6H, s), 1.47 (9H, s); ^{13}C NMR (126 MHz, $CDCl_3$) ^{13}C NMR (126 MHz, $CDCl_3$) δ 157.8, 156.3, 118.9, 79.9, 55.3, 32.7, 28.4, 24.0; HRMS (APCI) found: MH^+ 263.1750, $C_{15}H_{23}O_2N_2$ requires 263.1754.

tert-Butyl 3-Vinylazetidine-1-carboxylate (33)

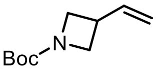

Following **GP4**, 1-Boc-3-iodoazetidine (19 µL, 0.10 mmol), boronate **2af** (0.50 mL, 0.50 M in THF, 0.25 mmol) gave **33** (12 mg, 65%) as an oil. ^{1}H NMR (500 MHz, $CDCl_3$) δ 5.99 (1H, ddd, *J* = 16.9, 10.4, 7.8 Hz), 5.14–5.02 (2H, m), 4.08 (2H, t, *J* = 8.5 Hz), 3.73 (2H, dd, *J* = 8.5, 5.9 Hz), 3.24–3.12 (1H, m), 1.43 (9H, s); ^{13}C NMR (126 MHz, $CDCl_3$) δ 156.5, 138.9, 115.8, 79.5, 54.5, 32.3, 28.6; HRMS (ESI) found: MNa^+ 206.1149, $C_{10}H_{17}O_2NNa$ requires 206.1152. Data in accordance with the literature.[23]

tert-Butyl (_E_)-3-Styrylazetidine-1-carboxylate (34)

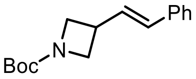

Following **GP4**, 1-Boc-3-iodoazetidine (19 µL, 0.10 mmol), boronate **2ag** (0.50 mL, 0.50 M in THF, 0.25 mmol) gave **34** (23 mg, 90%) as an oil. ^{1}H NMR (400 MHz, $CDCl_3$) δ: 7.38–7.20

(5H, m), 6.49–6.31 (2H, m), 4.16 (2H, t, *J* = 8.5 Hz), 3.83 (2H, dd, *J* = 8.6, 5.9 Hz), 3.41–3.29 (1H, m), 1.46 (9H, s). Data in accordance with the literature.[24]

***tert*-Butyl (*E*)-3-(Hex-1-en-1-yl)azetidine-1-carboxylate (35)**

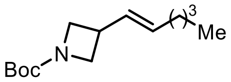

Following **GP4**, 1-Boc-3-iodoazetidine (19 µL, 0.10 mmol), boronate **2ah** (0.50 mL, 0.50 M in THF, 0.25 mmol) gave **35** (23 mg, 98%) as an oil. ^{1}H NMR (400 MHz, $CDCl_3$) δ 5.61–5.40 (2H, m), 4.04 (2H, t, *J* = 8.4 Hz), 3.67 (2H, dd, *J* = 8.5, 6.0 Hz), 3.16–3.07 (1H, m), 2.05–1.95 (2H, m), 1.41 (9H, s), 1.37-1.10 (4H, m), 0.88 (3H, t, *J* = 6.3 Hz). Data in accordance with the literature.[25]

***tert*-Butyl (*E*)-3-(3-Methoxyprop-1-en-1-yl)azetidine-1-carboxylate (36)**

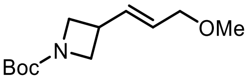

Following **GP4**, 1-Boc-3-iodoazetidine (19 µL, 0.10 mmol), boronate **2ai** (0.50 mL, 0.50 M in THF, 0.25 mmol) gave **36** (22 mg, 99%) as an oil. ^{1}H NMR (400 MHz, $CDCl_3$) δ 5.88 (1H, ddt, *J* = 15.5, 8.0, 1.4 Hz), 5.63 (1H, dt, *J* = 15.5, 5.9 Hz), 4.07 (2H, t, *J* = 8.5 Hz), 3.89 (2H, d, *J* = Hz), 3.73 (2H, dd, *J* = 8.5, 6.0 Hz), 3.33 (3H, s), 3.26–3.10 (1H, m), 1.43 (9H, s); ^{13}C NMR (101 MHz, $CDCl_3$) δ 156.5, 134.0, 127.9, 79.5, 72.7, 58.2, 54.7, 31.2, 28.5; HRMS (ESI) found: MNa^+ 250.1407, $C_{12}H_{21}O_3NNa$ requires 250.1414.

***tert*-Butyl (*E*)-3-(3-((*tert*-Butyldimethylsilyl)oxy)prop-1-en-1-yl)azetidine-1-carboxylate (37)**

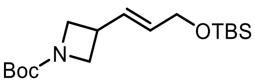

Following **GP4**, 1-Boc-3-iodoazetidine (19 µL, 0.10 mmol), boronate **2aj** (0.50 mL, 0.50 M in THF, 0.25 mmol) gave **37** (32 mg, 99%) as an oil. ^{1}H NMR (500 MHz, $CDCl_3$) δ 5.83 (1H, dd, *J* = 15.3, 8.1 Hz), 5.61 (1H, dtd, *J* = 15.2, 4.9, 1.1 Hz), 4.15 (2H, ddd, *J* = 5.0, 1.7, 0.8 Hz), 4.07 (2H, t, *J* = 8.5 Hz), 3.72 (2H, dd, *J* = 8.5, 6.0 Hz), 3.25–3.14 (1H, m), 1.44 (9H, s), 0.91 (9H, s), 0.07 (6H, s); ^{13}C NMR (126 MHz, $CDCl_3$) δ 156.5, 131.0, 130.8, 79.5, 63.5, 54.8, 31.1, 28.6, 26.1, 18.6, –5.0; HRMS (ESI) found: $2MH^+$ 655.4527, $C_{34}H_{66}O_6N_2Si_2H$ requires 655.4532.

***tert*-Butyl 3-(3,6-Dihydro-2*H*-pyran-4-yl)azetidine-1-carboxylate (38)**

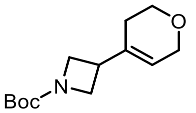

Following **GP4**, 1-Boc-3-iodoazetidine (19 µL, 0.10 mmol), boronate **2ak** (0.50 mL, 0.50 M in THF, 0.25 mmol) gave **38** (13 mg, 99%) as an oil. ^{1}H NMR (500 MHz, $CDCl_3$) δ 5.56–5.53 (1H, m), 4.17–4.14 (2H, m), 4.02 (2H, t, *J* = 8.6 Hz), 3.86–3.78 (4H, m), 3.19–3.10 (1H, m), 2.09 (2H, td, *J* = 5.3, 2.6 Hz), 1.44 (9H, s); ^{13}C NMR (126 MHz, $CDCl_3$) δ 156.5, 134.6, 121.0, 79.6, 65.5, 64.2, 52.6, 34.3, 28.6, 25.8; HRMS (ESI) found: MNa^+ 262.1409, $C_{13}H_{21}NO_3Na$ requires 262.1414. Data in accordance with the literature.[26]

***tert*-Butyl 3-(2-Methylprop-1-en-1-yl)azetidine-1-carboxylate (39)**

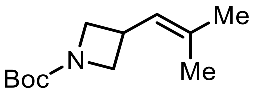

Following **GP4**, 1-Boc-3-iodoazetidine (19 µL, 0.10 mmol), boronate **2al** (0.50 mL, 0.50 M in THF, 0.25 mmol) gave **39** (15 mg, 72%) as an oil. ^{1}H NMR (400 MHz, $CDCl_3$) δ 5.35 (1H, dq, *J* = 9.0, 1.4 Hz), 4.09 (2H, t, *J* = 8.4 Hz), 3.63 (2H, dd, *J* = 8.3, 6.1 Hz), 3.44–3.27 (1H, m), 1.70 (3H, s), 1.57 (3H, s), 1.43 (9H, s); ^{13}C NMR (101 MHz, $CDCl_3$) δ 156.5, 134.1, 126.3, 79.3, 55.9, 28.6, 27.5, 25.7, 18.2; HRMS (ESI) found: MNa^+ 234.1454, $C_{12}H_{21}O_2NNa$ requires 234.1465.

***tert*-Butyl 3-(Phenylethynyl)azetidine-1-carboxylate (40)**

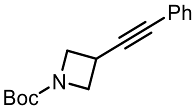

Following **GP4**, 1-Boc-3-iodoazetidine (19 µL, 0.10 mmol), boronate **2am** (0.50 mL, 0.50 M in THF, 0.25 mmol) gave **40** (9 mg, 34%) as an oil. ^{1}H NMR (400 MHz, $CDCl_3$) δ 7.43–7.38 (2H, m), 7.33–7.28 (3H, m), 4.21 (2H, t, *J* = 8.4 Hz), 4.02 (2H, dd, *J* = 8.1, 6.4 Hz), 3.54 (1H, tt, *J* = 8.6, 6.4 Hz), 1.45 (9H, s). Data in accordance with the literature.[24]

***tert*-Butyl 3-Benzylazetidine-1-carboxylate (41)**

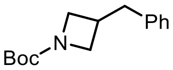

Following **GP4** but using cumOOSi*t*-BuPh$_2$ (117 mg, 0.300 mmol) as oxidant, 1-Boc-3-iodoazetidine (19 µL, 0.10 mmol), boronate **2an** (0.50 mL, 0.50 M in THF, 0.25 mmol) gave

41 (14 mg, 57%) as an oil. ^{1}H NMR (500 MHz, $CDCl_3$) δ 7.33–7.27 (2H, m), 7.23–7.19 (1H, m) 7.18–7.11 (2H, m), 3.99 (2H, t, *J* = 8.3 Hz), 3.65 (2H, dd, *J* = 8.7, 5.4 Hz), 2.90 (2H, d, *J* = 8.0 Hz,), 2.81 (1H, pt, *J* = 8.1, 5.4 Hz), 1.44 (9H, s,). ^{13}C NMR (126 MHz, $CDCl_3$) δ 156.6, 139.5, 128.7, 128.5, 126.5, 79.4, 54.4, 40.4, 30.0, 28.6. Data in accordance with the literature.[27]

tert-Butyl 3-(3-Chlorobenzyl)azetidine-1-carboxylate (42)

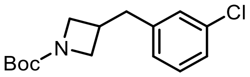

Following **GP4** but using cumOOSi*t*-BuPh$_2$ (117 mg, 0.300 mmol) as oxidant, 1-Boc-3-iodoazetidine (19 µL, 0.10 mmol), boronate **2ao** (0.50 mL, 0.50 M in THF, 0.25 mmol) gave **42** (17 mg, 62%) as a solid. ^{1}H NMR (500 MHz, $CDCl_3$) δ 7.24–7.17 (2H, m), 7.14–7.12 (1H, m), 7.02 (1H, dt, *J* = 7.1, 1.7 Hz), 3.99 (2H, t, *J* = 8.3 Hz), 3.63 (2H, dd, *J* = 8.6, 5.3 Hz), 2.88 (2H, d, *J* = 8.0 Hz), 2.84–2.73 (1H, m), 1.44 (9H, s). ^{13}C NMR (126 MHz, $CDCl_3$) δ 156.6, 141.5, 134.5, 130.0, 128.7, 126.8, 126.7, 79.5, 54.2, 40.0, 29.8, 28.6. HRMS (ESI) found: MNa^+ 304.1067, $C_{15}H_{20}O_2NClNa$ requires 304.1075.

tert-Butyl 3-(3-(Trifluoromethyl)benzyl)azetidine-1-carboxylate (43)

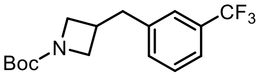

Following **GP4** but using cumOOSi*t*-BuPh$_2$ (117 mg, 0.300 mmol) as oxidant, 1-Boc-3-iodoazetidine (19 µL, 0.10 mmol), boronate **2ap** (0.50 mL, 0.50 M in THF, 0.25 mmol) gave **43** (20 mg, 64%) as a solid. ^{1}H NMR (500 MHz, $CDCl_3$) δ 7.48 (1H, d, *J* = 7.8 Hz), 7.44–7.38 (2H, m), 7.33 (1H, d, *J* = 7.6 Hz), 4.00 (2H, t, *J* = 8.3 Hz), 3.64 (2H, dd, *J* = 8.6, 5.3 Hz), 2.97 (2H, d, *J* = 8.0 Hz), 2.88–2.77 (1H, m), 1.44 (9H, s); ^{13}C NMR (126 MHz, $CDCl_3$) δ 156.6, 140.4, 131.9, 131.1 (q, *J* = 31.9 Hz), 129.2, 125.2 (q, *J* = 3.8 Hz), 124.2 (q, *J* = 271.9 Hz), 123.5 (q, *J* = 3.7 Hz), 79.6, 53.9, 40.1, 29.8, 28.6; ^{19}F NMR (376 MHz, $CDCl_3$) δ –62.6; HRMS (ESI) found: MNa^+ 338.1328, $C_{16}H_{20}O_2NF_3Na$ requires 338.1338.

tert-Butyl 3-Allylazetidine-1-carboxylate (44)

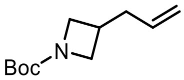

Following **GP4**, 1-Boc-3-iodoazetidine (19 µL, 0.10 mmol), boronate **2aq** (0.50 mL, 0.50 M in THF, 0.25 mmol) gave **44** (13 mg, 65%) as an oil. ^{1}H NMR (400 MHz, $CDCl_3$) δ 5.73 (1H, ddt, *J* = 17.6, 9.8, 6.5 Hz), 5.08–4.97 (2H, m), 3.99 (2H, dd, *J* = 8.7, 8.0 Hz), 3.56 (2H, dd, *J* = 8.5,

5.4 Hz), 2.58 (1H, tt, *J* = 7.9, 5.4 Hz), 2.32 (2H, ddt, *J* = 7.8, 6.5, 1.4 Hz), 1.43 (9H, s); ^{13}C NMR (126 MHz, $CDCl_3$) δ 156.6, 135.2, 116.5, 79.3, 54.1, 38.3, 28.6, 28.0; HRMS (ESI) found MNa^+ 220.1303, $C_{11}H_{19}O_2NNa$ requires 220.1308.

***tert*-Butyl 4-Benzylpiperidine-1-carboxylate (47)**

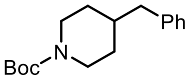

Following **GP4** but using $[Cu(MeCN)_4]PF_6$ (4 mg, 10%mol), cumOOTES (80 mg, 85 μL, 0.30 mmol, 3.0 equiv.) and no TMHD, *tert*-butyl 4-(iodomethyl)piperidine-1-carboxylate (33 mg, 0.10 mmol) and boronate **2a** (0.50 mL, 0.50 M in THF, 0.25 mmol) gave **47 (**15 mg, 54%) as an oil. ^1H NMR (400 MHz, $CDCl_3$) δ 7.31–7.24 (2H, m), 7.23–7.16 (1H, m), 7.16–7.11 (2H, m), 4.06 (2H, br. s), 2.63 (2H, t, *J* = 12.4 Hz), 2.53 (2H, d, *J* = 7.0 Hz), 1.69–1.57 (4H, m), 1.45 (9H, s), 1.21–1.08 (2H, m). Data in accordance with the literature.[28]

***tert*-Butyl 4-Phenylpiperidine-1-carboxylate (48)**

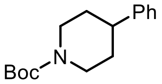

Following **GP4** but using cumOOTES (80 mg, 85 μL, 0.30 mmol, 3.0 equiv.) and no TMHD, *tert*-butyl 4-iodopiperidine-1-carboxylate (31 mg, 0.10 mmol) and boronate **2a** (0.50 mL, 0.50 M in THF, 0.25 mmol) gave **48** (16 mg, 62%) as an oil. ^1H NMR (400 MHz, $CDCl_3$) δ 7.34–7.28(2H, m), 7.24–7.18(3H, m), 4.25 (2H, d, J= 13.3 Hz), 2.80 (2H, dt, J= 13.0, 2.6 Hz), 2.64 (1H, tt, J= 12.2, 3.6 Hz), 1.86–1.78 (2H, m), 1.69–1.56 (2H, m), 1.48 (9H, s). Data in accordance with the literature.[29]

2-(4-Phenylpiperidin-1-yl)pyrimidine (49)

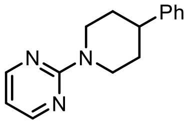

Following **GP4** but using cumOOTES (80 mg, 85 μL, 0.30 mmol, 3.0 equiv.) and no TMHD, 2-(4-iodopiperidin-1-yl)pyrimidine **S10** (29 mg, 0.10 mmol) and boronate **2a** (0.50 mL, 0.50 M in THF, 0.25 mmol) gave **49** (14 mg, 59%) as an oil. ^1H NMR (500 MHz, $CDCl_3$) δ 8.32 (2H, d, *J* = 4.7 Hz), 7.33–7.29 (2H, m), 7.24–7.18 (3H, m), 6.47 (1H, t, *J* = 4.7 Hz), 4.97–4.89 (2H, m), 2.97 (2H, td, *J* = 13.0, 2.6 Hz), 2.80 (1H, tt, *J* = 12.2, 3.6 Hz), 1.98–1.91 (2H, m), 1.71 (2H,

qd, J = 12.6, 4.2 Hz). ^{13}C NMR (126 MHz, $CDCl_3$) δ 161.6, 157.8, 146.0, 128.5, 126.8, 126.3, 109.4, 44.5, 43.1, 33.1. HRMS (ASAP): Found MH^+ 240.1492, $C_{15}H_{18}N_3$ requires 240.1495.

***tert*-Butyl 3-Phenyl-8-azabicyclo[3.2.1]octane-8-carboxylate (50)**

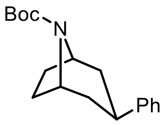

Following **GP4** but using cumOOTES (80 mg, 85 µL, 0.30 mmol, 3.0 equiv.) and no TMHD, tert-butyl 3-exo-iodo-8-azabicyclo[3.2.1]octane-8-carboxylate **S7** (34 mg, 0.10 mmol) and boronate **2a** (0.50 mL, 0.50 M in THF, 0.25 mmol) gave **50** (16 mg, 56%) as an oil. dr = 1.15:1. ^{1}H NMR (400 MHz, $CDCl_3$) δ 7.33–7.26 (2H, m), 7.25–7.15 (3H, m), 4.39–4.19 (2H, m), 3.08 (0.5H, tt, J = 11.9, 5.4 Hz), 2.65 (0.5H, tt, J = 10.4, 7.1 Hz), 2.56–2.39 (1H, m), 2.10–1.90 (3H, m), 1.87–1.60 (4H, m), 1.50 (9H, s). ^{13}C NMR (101 MHz, $CDCl_3$, diastereomers and rotamers) δ 154.6, 153.6, 145.2, 144.8, 128.5, 128.3, 127.4, 127.1, 126.3, 126.0, 79.14, 79.11, 54.2, 53.4, 51.6, 51.0, 35.2, 34.3, 31.7, 31.1, 28.5, 28.5, 27.8. HRMS (ESI): Found MNa^+ 310.1768, $C_{18}H_{25}O_2NNa$ requires 310.1768.

4-Phenyltetrahydro-2*H*-pyran (51)

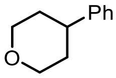

Following **GP4** but using cumOOTES (80 mg, 85 µL, 0.30 mmol, 3.0 equiv.) and no TMHD, 4-iodotetrahydro-2*H*-pyran (21.2 mg, 0.10 mmol) and boronate **2a** (0.50 mL, 0.50 M in THF, 0.25 mmol) gave **51** (11 mg, 66%) as an oil. ^{1}H NMR (400 MHz, $CDCl_3$) δ 7.33 (2H, d, J = 7.4 Hz), 7.26–7.18 (3H, m), 4.08 (2H, dd, J = 11.4, 4.3 Hz), 3.53 (2H, td, J = 11.5, 2.7 Hz), 2.75 (1H, tt, J = 11.5, 4.4 Hz), 1.90–1.72 (4H, m). Data in accordance with the literature.[30]

2-(4-Bromophenyl)-4-phenyltetrahydro-2*H*-pyran (52)

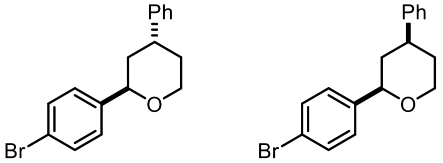

Following **GP4** but using cumOOTES (80 mg, 85 µL, 0.30 mmol, 3.0 equiv.) and no TMHD, 2-(4-bromophenyl)-4-iodotetrahydro-2*H*-pyran **S16** (37 mg, 0.10 mmol) and boronate **2a** (0.50 mL, 0.50 M in THF, 0.25 mmol) gave **52** (15 mg, 47%) as an oil as an unseparable mixture of diastereomers. dr = 1.8:1. ^{1}H NMR (500 MHz, $CDCl_3$, diastereomers) δ 7.51 (1.3H, d, J = 8.5

Hz), 7.46 (0.7H, d, *J* = 8.5 Hz), 7.38–7.28 (4H, m), 7.28–7.20 (3H, m), 4.90 (0.65H, t, *J* = 4.9 Hz), 4.45 (0.35H, dd, *J* = 11.1, 2.2 Hz), 4.29 (0.35H, ddd, *J* = 11.6, 4.2, 2.1 Hz), 3.84 (0.65H, ddd, *J* = 11.7, 6.0, 3.9 Hz), 3.81–3.72 (1H, m), 3.03 (0.65H, tt, *J* = 8.5, 4.5 Hz), 2.96 (0.35H, tt, *J* = 11.6, 4.1 Hz), 2.36–2.25 (1.3H, m), 2.10–1.96 (1H, m), 1.96–1.81 (1.3H, m), 1.75–1.65 (0.4H, m); ^{13}C NMR (126 MHz, $CDCl_3$, diastereomers) δ 145.2, 144.6, 141.8, 140.4, 131.6, 131.4, 128.6, 128.6, 128.2, 127.5, 127.1, 126.7, 126.5, 126.3, 121.1, 120.9, 79.2, 73.4, 68.7, 62.8, 42.0, 41.4, 35.7, 35.5, 33.2, 32.2. HRMS (ASAP): Found $2MH^+$ 633.0999, $C_{34}H_{45}O_2Br_2$ requires 633.0998.

4-Phenyltetrahydro-2*H*-thiopyran (53)

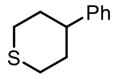

Following **GP4** but using cumOOTES (80 mg, 85 μL, 0.30 mmol, 3.0 equiv.) and no TMHD, 4-iodotetrahydro-2*H*-thiopyran **S4** (22.8 mg, 0.10 mmol) and boronate **2a** (0.50 mL, 0.50 M in THF, 0.25 mmol) gave **53** (11.0 mg, 62%) as an oil. ^{1}H NMR (500 MHz, $CDCl_3$) δ 7.33–7.28 (2H, m), 7.23–7.17 (3H, m), 2.85 (2H, ddd, *J* = 13.7, 12.3, 2.4 Hz), 2.73–2.68 (2H, m), 2.52 (1H, tt, *J* = 12.2, 3.2 Hz), 2.19–2.10 (2H, m), 1.91–1.82 (2H, m); ^{13}C NMR (126 MHz, $CDCl_3$) δ 146.9, 128.5, 126.8, 126.3, 44.3, 35.1, 29.3. HRMS (ASAP): Found MH^+ 179.0885, $C_{11}H_{15}SNa$ requires 179.0889.

3-Phenyloxetane (54)

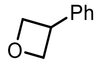

Following **GP4** but using cumOOTES (80 mg, 85 μL, 0.30 mmol, 3.0 equiv.) and no TMHD, 3-iodooxetane (18.4 mg, 0.10 mmol) and boronate **2a** (0.50 mL, 0.50 M in THF, 0.25 mmol) gave **54** (10 mg, 74%) as an oil. ^{1}H NMR (400 MHz, $CDCl_3$) δ 7.42–7.36 (4H, m), 7.30–7.27 (1H, m), 5.08 (2H, dd, *J* = 8.4, 6.0 Hz), 4.79 (2H, dd, J= 6.8, 6.0 Hz), 4.29–4.19 (1H, m). Data in accordance with the literature.[31]

Diisopropyl 3-Phenylcyclobutane-1,1-dicarboxylate (55)

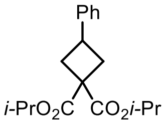

Following **GP4** but using $Cu(MeCN)_4PF_6$ (11 mg, 0.030 mmol, 30 mol%), cumOOTES (80 mg, 85 μL, 0.30 mmol, 3.0 equiv.) and no TMHD, diisopropyl 3-iodocyclobutane-1,1-

dicarboxylate **S1** (35 mg, 0.10 mmol) and boronate **2a** (0.50 mL, 0.50 M in THF, 0.25 mmol) gave **55** (21 mg, 68%) as an oil. ^{1}H NMR (400 MHz, $CDCl_3$) δ 7.34–7.28 (2H, m), 7.25–7.18 (3H, m), 5.15 (1H, hept, *J* = 6.3 Hz), 5.05 (1H, hept, *J* = 6.3 Hz), 3.61 (1H, p, *J* = 9.3 Hz), 2.94–2.85 (2H, m), 2.71–2.62 (2H, m), 1.28 (6H, d, *J* = 6.3 Hz), 1.24 (6H, d, *J* = 6.3 Hz). ^{13}C NMR (101 MHz, $CDCl_3$) δ 171.5, 170.9, 144.2, 128.4, 126.5, 126.4, 68.9, 68.7, 49.2, 36.1, 33.8, 21.6, 21.5. HRMS (ASAP): Found MH^+ 305.1741, $C_{18}H_{25}O_4$ requires 305.1747.

4-Phenylcyclohexan-1-one (56)

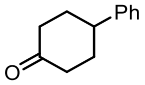

Following **GP4** but using cumOOTES (80 mg, 85 μL, 0.30 mmol, 3.0 equiv.) and no TMHD, 4-iodocyclohexan-1-one **S9** (22 mg, 0.10 mmol) and boronate **2a** (0.50 mL, 0.50 M in THF, 0.25 mmol) gave **56** (9 mg, 53%) as an oil. ^{1}H NMR (400 MHz, $CDCl_3$) δ 7.36−7.29 (2H, m), 7.28−7.20 (3H, m), 3.03 (1H, tt,J= 12.2, 3.7 Hz), 2.58−2.44 (4H, m), 2.32−2.19(2H, m), 2.01−1.89 (2H, m). Data in accordance with the literature.[29]

8-Phenyl-1,4-dioxaspiro[4.5]decane (57)

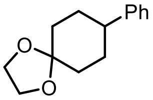

Following **GP4** but using cumOOTES (80 mg, 85 μL, 0.30 mmol, 3.0 equiv.) and no TMHD, 8-iodo-1,4-dioxaspiro[4.5]decane **S3** (27 mg, 0.10 mmol) and boronate **2a** (0.50 mL, 0.50 M in THF, 0.25 mmol) gave **57** (13 mg, 61%) as an oil. ^{1}H NMR (400 MHz, $CDCl_3$) δ 7.35−7.26 (2H, m), 7.26−7.16 (3H, m), 3.99 (4H, s), 2.61−2.49 (1H, m), 1.91−1.84 (4H, m), 1.83−1.74 (2H, m), 1.74−1.65 (2H, m). Data in accordance with the literature.[29]

***trans*-2-Methoxycyclohexyl)benzene (58)**

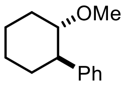

Following **GP4** but using cumOOTES (80 mg, 85 μL, 0.30 mmol, 3.0 equiv.) and no TMHD, trans-1-iodo-2-methoxycyclohexane **S13** (24.0 mg, 0.10 mmol) and boronate **2a** (0.50 mL, 0.50 M in THF, 0.25 mmol) gave **58 (**19 mg, 63%) as an oil. ^{1}H NMR (400 MHz, $CDCl_3$) δ 7.37–7.12 (5H, m), 3.27 (1H, dt, *J* = 4.7, 10.6 Hz), 3.10 (3H, s), 2.65–2.45 (1H, m), 2.32–2.20 (1H, m), 1.95–1.67 (3H, m), 1.60–1.15 (4H, m). Data in accordance with the literature.[32]

2-Phenyl-2,3-dihydro-1*H*-indene (59)

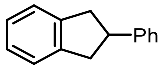

Following **GP4** but using $Cu(MeCN)_4PF_6$ (11 mg, 30%mol), cumOOTES (80 mg, 85 μL, 0.30 mmol, 3.0 equiv.) and no TMHD, 2-iodo-2,3-dihydro-1*H*-indene **S8** (24 mg, 0.10 mmol) and boronate **2a** (0.50 mL, 0.50 M in THF, 0.25 mmol) gave **59** (12 mg, 64%) as an oil. ^{1}H NMR (400 MHz, $CDCl_3$) δ 7.30 –7.24 (4H, m), 7.22 –7.12 (5H, m), 3.65 (1H, p, J= 8.6 Hz), 3.31 (2H, dd, J= 15.5, 8.2 Hz), 3.05 (2H, dd, J= 15.5, 9.0 Hz). Data in accordance with the literature.[33]

Propane-1,2-diyldibenzene (60)

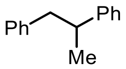

Following **GP4** but using cumOOTES (80 mg, 85 μL, 0.30 mmol, 3.0 equiv.) and no TMHD, (2-iodopropyl)benzene **S2** (24.6 mg, 0.10 mmol) and boronate **2a** (0.50 mL, 0.50 M in THF, 0.25 mmol) gave **60** (17 mg, 73%) as an oil. ^{1}H NMR (400 MHz, $CDCl_3$) δ 7.34–7.15 (8H, m), 7.13–7.07 (2H, m), 3.08–2.93 (2H, m), 2.83–2.75 (1H, m), 1.26 (3H, d, *J* = 6.8 Hz). Data in accordance with the literature.[34]

4-Phenyl-1-oxaspiro[5.5]undecane (61)

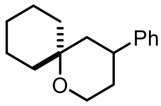

Following **GP4** but using cumOOTES (80 mg, 85 μL, 0.30 mmol, 3.0 equiv.) and no TMHD, 4-iodo-1-oxaspiro[5.5]undecane **S15** (28.0 mg, 0.10 mmol) and boronate **2a** (0.50 mL, 0.50 M in THF, 0.25 mmol) gave **61** (14 mg, 73%) as an oil. ^{1}H NMR (400 MHz, $CDCl_3$) δ 7.33–7.28 (2H, m), 7.23–7.18 (3H, m), 3.85–3.73 (1H, m), 2.97–2.90 (1H, m), 2.14–2.10 (1H, m), 1.80–1.26 (14H, m). Data in accordance with the literature.[35]

***tert*-Butyl 2-Phenyl-7-azaspiro[3.5]nonane-7-carboxylate (62)**

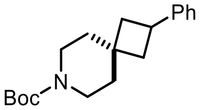

Following **GP4** but using cumOOTES (80 mg, 85 μL, 0.30 mmol, 3.0 equiv.) and no TMHD, *tert*-butyl 2-iodo-7-azaspiro[3.5]nonane-7-carboxylate **S6** (35.1 mg, 0.10 mmol) and boronate **2a** (0.50 mL, 0.50 M in THF, 0.25 mmol) gave **62** (6 mg, XX%) as an oil. ^{1}H NMR (400 MHz, $CDCl_3$) δ 7.36–7.26 (2H, m), 7.24–7.14 (3H, m), 3.52 (1H, p, *J* = 9.1 Hz), 3.45–3.38 (2H, m),

3.34–3.25 (2H, m), 2.35–2.24 (2H, m), 1.95–1.85 (2H, m), 1.74–1.68 (2H, m), 1.54–1.48 (2H, m), 1.47 (9H, s). Data in accordance with the literature.[36]

***tert*-Butyl 6-Phenyl-2-azaspiro[3.3]heptane-2-carboxylate (63)**

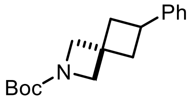

Following **GP4**, but using cumOOTES (80 mg, 85 μL, 0.30 mmol, 3.0 equiv.) and no TMHD, *tert*-butyl 6-iodo-2-azaspiro[3.3]heptane-2-carboxylate **S5** (32 mg, 0.10 mmol) and boronate **2a** (0.50 mL, 0.50 M in THF, 0.25 mmol) gave **63** (18 mg, 65%) as an oil. ^{1}H NMR (500 MHz, $CDCl_3$) δ 7.34–7.28 (2H, m), 7.22–7.14 (3H, m), 4.06 (2H, s), 3.84 (2H, s), 3.39 (1H, p, *J* = 8.9 Hz), 2.62–2.53 (2H, m), 2.32–2.25 (2H, m), 1.44 (9H, s). ^{13}C NMR (126 MHz, $CDCl_3$) δ 156.3, 144.6, 128.3, 126.2, 126.1, 79.3, 62.2 (br.), 60.2 (br.), 40.2, 34.1, 33.9, 28.4. HRMS (ESI): Found MNa^+ 296.1612, $C_{17}H_{23}O_2NNa$ requires 296.1621.

***tert*-Butyl 6-(3-Fluoro-4-methoxyphenyl)-2-azaspiro[3.3]heptane-2-carboxylate (64)**

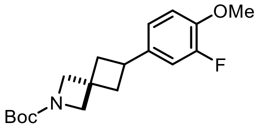

Following **GP4** but using cumOOTES (80 mg, 85 μL, 0.30 mmol, 3.0 equiv.) and no TMHD, iodide **S5** (32 mg, 0.10 mmol), boronate **2ar** (0.50 mL, 0.50 M in THF, 0.25 mmol) gave **64** (30 mg, 94%) as an oil. ^{1}H NMR (400 MHz, $CDCl_3$) δ 6.92–6.82 (3H, m), 4.04 (2H, s), 3.86 (3H, s), 3.83 (2H, s), 3.38–3.22 (1H, m), 2.62–2.44 (2H, m), 2.25–2.16 (2H, m), 1.44 (9H, s); ^{13}C NMR (101 MHz, $CDCl_3$) δ 156.4, 152.5 (d, *J* = 245.8 Hz), 146.0 (d, *J* = 10.8 Hz), 138.1 (d, *J* = 5.6 Hz), 121.9 (d, *J* = 3.4 Hz), 114.2 (d, *J* = 18.0 Hz), 113.5 (d, *J* = 2.2 Hz), 79.5, 62.3, 60.3, 56.6, 40.5, 34.0, 33.5, 28.6; ^{19}F NMR (376 MHz, $CDCl_3$) δ –135.3. HRMS (ESI) found MNa^+ 344.1632, $C_{18}H_{24}O_3NFNa$ requires 344.1632.

***tert*-Butyl 6-(4-(Trifluoromethoxy)phenyl)-2-azaspiro[3.3]heptane-2-carboxylate (65)**

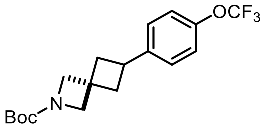

Following **GP4** but using cumOOTES (80 mg, 85 μL, 0.30 mmol, 3.0 equiv.) and no TMHD, iodide **S5** (32 mg, 0.10 mmol), boronate **2k** (0.50 mL, 0.50 M in THF, 0.25 mmol) gave **65** (29 mg, 81%) as an oil. ^{1}H NMR (500 MHz, $CDCl_3$) δ 7.17 (2H, d, *J* = 8.6 Hz), 7.13 (2H, d, *J* =

8.4 Hz), 4.06 (2H, s), 3.84 (2H, s), 3.39 (1H, t, *J* = 8.9 Hz), 2.64–2.53 (2H, m), 2.29–2.21 (2H, m), 1.44 (9H, s); ^{13}C NMR (126 MHz, $CDCl_3$) δ 156.4, 147.6 (d, *J* = 1.9 Hz), 143.5, 127.7, 121.1, 120.6 (q, *J* = 256.6 Hz), 79.5, 62.3, 60.3, 40.4, 34.0, 33.7, 28.6; ^{19}F NMR (376 MHz, $CDCl_3$) δ –58.0; HRMS (ESI) found: MNa^+ 380.1433, $C_{18}H_{22}O_3NF_3Na$ requires 380.1444.

1,3-Dimethyl-7-(2-phenylpropyl)-3,7-dihydro-1*H*-purine-2,6-dione (68)

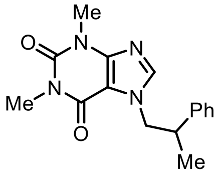

Following **GP4** but using cumOOTES (80 mg, 85 μL, 0.30 mmol, 3.0 equiv.) and no TMHD, 7-(2-iodopropyl)-1,3-dimethyl-3,7-dihydro-1*H*-purine-2,6-dione **S12** (35 mg, 0.10 mmol) and boronate **2a** (0.50 mL, 0.50 M in THF, 0.25 mmol) gave **68** (16 mg, 53%) as an oil. ^{1}H NMR (400 MHz, $CDCl_3$) δ 7.33–7.27 (2H, m), 7.26–7.20 (1H, m), 7.16–7.11 (2H, m), 7.04 (1H, s), 4.48 (1H, dd, *J* = 13.4, 6.6 Hz), 4.25 (1H, dd, *J* = 13.4, 8.5 Hz), 3.56 (3H, s), 3.44 (3H, s), 3.44–3.29 (1H, m), 1.35 (3H, d, *J* = 7.0 Hz). ^{13}C NMR (101 MHz, $CDCl_3$) δ 155.3, 151.7, 148.8, 142.2, 141.2, 128.8, 127.3, 127.0, 106.7, 54.3, 40.9, 29.8, 28.0, 18.0. HRMS (ASAP): Found MH^+ 299.1491, $C_{16}H_{19}O_2N_4$ requires 299.1503.

1,3-Dimethyl-7-(2-(1-methyl-1*H*-pyrazol-4-yl)propyl)-3,7-dihydro-1*H*-purine-2,6-dione (69)

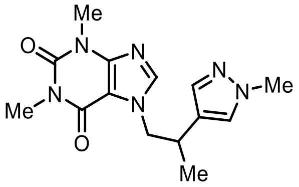

Following **GP4** but using cumOOTES (80 mg, 85 μL, 0.30 mmol, 3.0 equiv.) and no TMHD, 7-(2-iodopropyl)-1,3-dimethyl-3,7-dihydro-1*H*-purine-2,6-dione **S12** (35 mg, 0.10 mmol) and boronate **2z** (0.50 mL, 0.50 M in THF, 0.25 mmol) gave **69** (11 mg, 35%) as an oil. ^{1}H NMR (400 MHz, $CDCl_3$) δ 7.28 (1H, s), 7.27 (1H, s), 7.09 (1H, s), 4.32 (1H, dd, *J* = 13.3, 7.0 Hz), 4.23 (1H, dd, *J* = 13.3, 7.7 Hz), 3.84 (3H, s), 3.59 (3H, s), 3.43 (3H, s), 3.33 (1H, h, *J* = 7.1 Hz), 1.28 (3H, d, *J* = 7.0 Hz). ^{13}C NMR (101 MHz, $CDCl_3$) δ 155.2, 151.7, 148.9, 141.3, 137.1, 127.8, 122.8, 106.8, 54.4, 38.9, 31.4, 29.8, 28.0, 18.2. HRMS (ASAP): Found MH^+ 303.1561, $C_{14}H_{19}O_2N_6$ requires 303.1564.

(*E*)-7-(5-((*tert*-Butyldimethylsilyl)oxy)-2-methylpent-3-en-1-yl)-1,3-dimethyl-3,7-dihydro-1*H*-purine-2,6-dione (70)

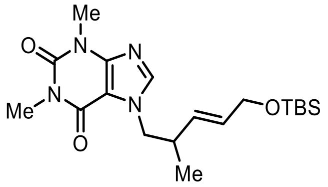

Following **GP4** but using cumOOTES (80 mg, 85 μL, 0.30 mmol, 3.0 equiv.) and no TMHD, 7-(2-iodopropyl)-1,3-dimethyl-3,7-dihydro-1*H*-purine-2,6-dione **S12** (35 mg, 0.10 mmol) and boronate **2aj** (0.50 mL, 0.50 M in THF, 0.25 mmol) gave **70** (16 mg, 42%) as an oil. ^{1}H NMR (400 MHz, $CDCl_3$) δ 7.45 (1H, s), 5.57–5.37 (2H, m), 4.23 (1H, dd, *J* = 13.4, 6.5 Hz), 4.13–4.03 (3H, m), 3.58 (3H, s), 3.41 (3H, s), 2.86–2.72 (1H, m), 1.06 (3H, d, *J* = 6.8 Hz), 0.88 (9H, s), 0.03 (6H, s). ^{13}C NMR (101 MHz, $CDCl_3$) δ 155.2, 151.7, 148.9, 141.3, 131.5, 130.6, 106.9, 63.2, 52.6, 37.8, 29.8, 28.0, 25.9, 18.4, 17.4, –5.3. HRMS (ASAP): Found MH^+ 393.2310, $C_{19}H_{33}O_3N_4Si$ requires 393.2316.

4,5-*cis*-3-Benzylhexahydro-4H-furo[2,3-b]pyran (72)

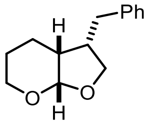

Following **GP4** but using cumOOTES (80 mg, 85 μL, 0.30 mmol, 3.0 equiv.) and no TMHD, 2-(allyloxy)-3-iodotetrahydro-2*H*-pyran **S14** (26.8 mg, 0.10 mmol) and boronate **2a** (0.50 mL, 0.50 M in THF, 0.25 mmol) gave **72** (12 mg, d.r. 12:1, 53%) as an oil. ^{1}H NMR (400 MHz, $CDCl_3$) δ 7.29 (2H, t, *J* = 7.4 Hz), 7.20 (2H, t, *J* = 7.5 Hz), 7.17 (1H, d, *J* = 7.2 Hz), 5.28 (1H, d, *J* = 3.7 Hz), 3.88 (1H, t, *J* = 7.7 Hz), 3.78 (2H, t, *J* = 8.7 Hz), 3.65 (1H, d, *J* = 11.3 Hz), 2.78–2.59 (3H, m), 2.00–1.91 (1H, m), 1.81–1.72 (1H, m), 1.66–1.51 (3H, m). Data in accordance with the literature.[37]

3-Benzyl-1-tosylpyrrolidine (74)

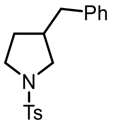

Following **GP4** but using cumOOTES (80 mg, 85 μL, 0.30 mmol, 3.0 equiv.) and no TMHD, *N*-allyl-*N*-(2-iodoethyl)-4-methylbenzenesulfonamide **S11** (36.5 mg, 0.10 mmol) and boronate **2a** (0.50 mL, 0.50 M in THF, 0.25 mmol) gave **74** (15 mg, 48%) as an oil. ^{1}H NMR (400 MHz, $CDCl_3$) δ 7.70 (2H, d, *J* = 8.2 Hz), 7.32 (2H, d, *J* = 7.9 Hz), 7.29–7.16 (3H, m), 7.05 (2H, d, *J* =

6.8 Hz), 3.42–3.31 (2H, m), 3.19 (1H, dt, *J* = 9.2, 8.0 Hz), 2.91 (1H, dd, *J* = 9.9, 7.5 Hz), 2.54 (2H, d, *J* = 7.7 Hz), 2.44 (3H, s), 2.37–2.27 (1H, m), 1.90–1.84 (1H, m), 1.55–1.46 (1H, m). Data in accordance with the literature.[38]

8 Mechanistic Studies

8.1 Proposed Mechanism

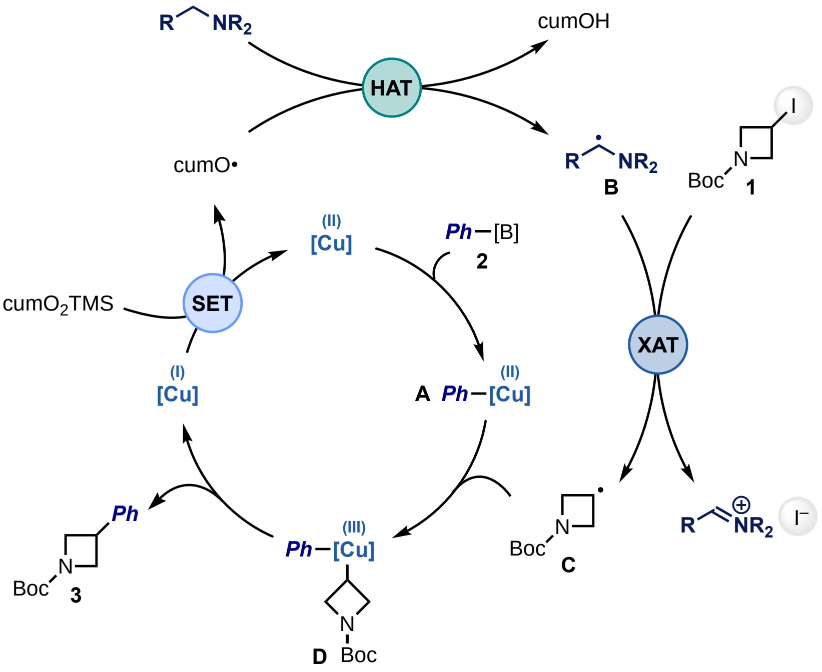

Scheme S4.

8.1.1 Probing the Oxidation of [Cu(I)]

The oxidation of [Cu(I)] to [Cu(II)] by cumO_2TMS was demonstrated by UV/Vis absorption spectroscopy studies showing the formation of a new band (λ = 750 nm), which is characteristic for [Cu(II)] species.

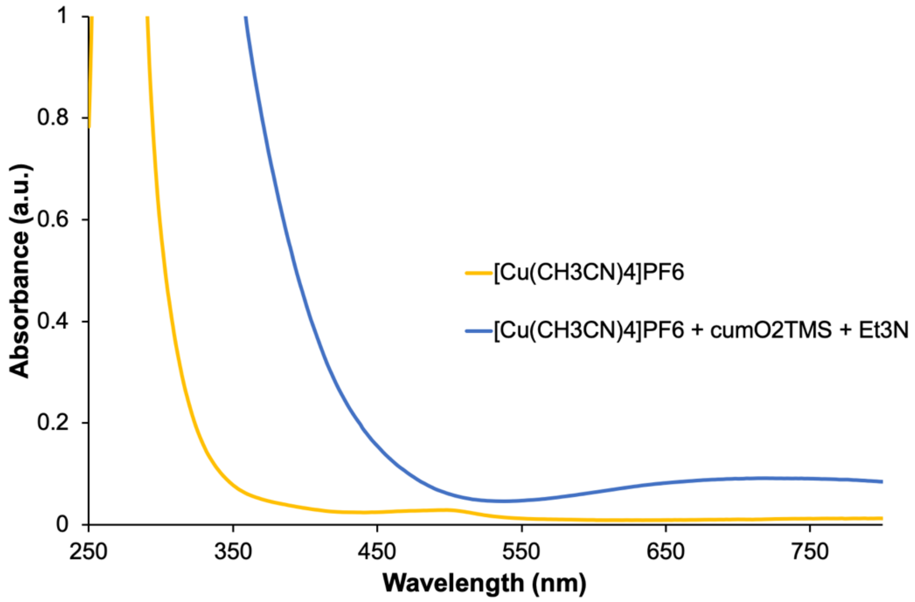

Figure S1.

8.1.2 Probing the Transmetalation of 2a with [Cu(II)]

The transmetalation of **2a** with [Cu(II)] was supported by UV/Vis absorption spectroscopy studies (**Figure S2**).

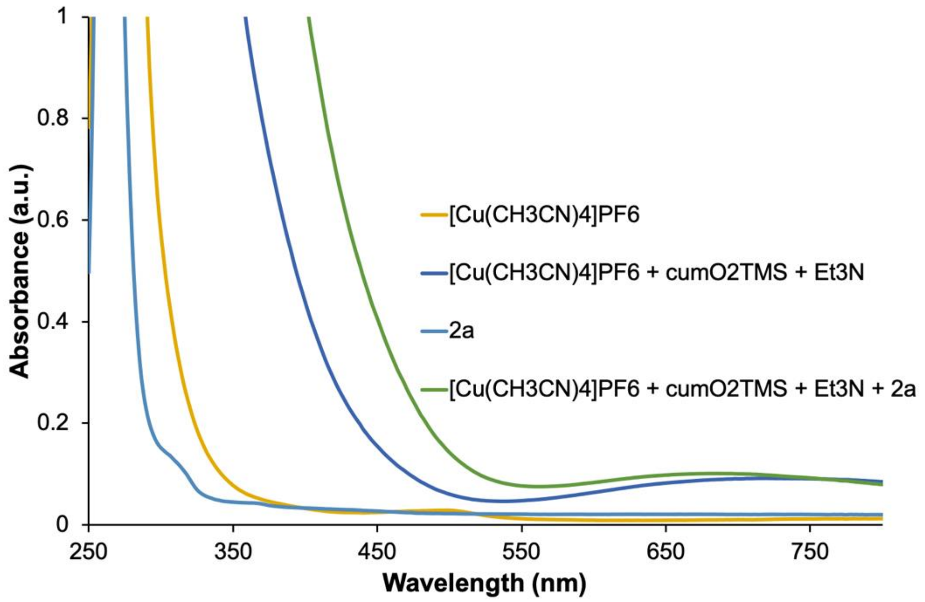

Figure S2.

The transmetalation between **2a** and [Cu(II)] should lead to the formation of Bu–B(pin), which was supported by ^{11}B NMR spectroscopy studies and GC-MS analysis (**Figure S3**).

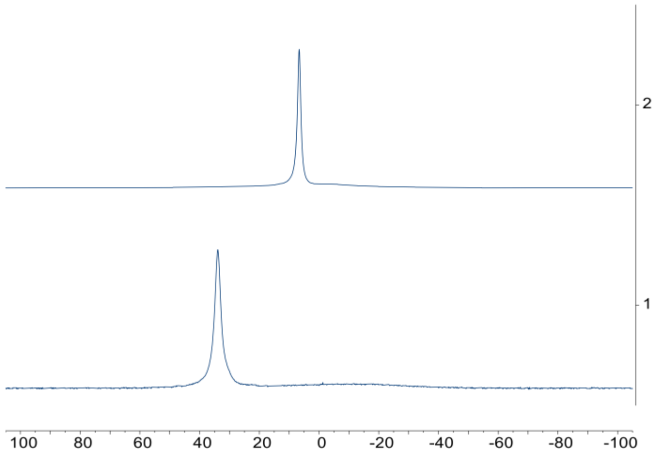

^{11}B NMR 2 = **2a**; ^{11}B NMR 1 = **2a** + $Cu(OTf)_2$

Figure S3.

Furthermore, a stoichiometric reaction between **2a** and [Cu(II)] followed by water work up gave Bu–B(pin) (**Scheme S5**).

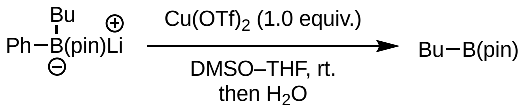

Scheme S5.

In order to obtain initial information regarding the lack of reactivity of organoborons **2b–d**, we have evaluated the direct transmetalation of these species with [Cu(II)] by UV/VIS absorption spectroscopy (**Figure S4**). These studies indicates that these species might not be able to react with [Cu(II)] under our reaction conditions.

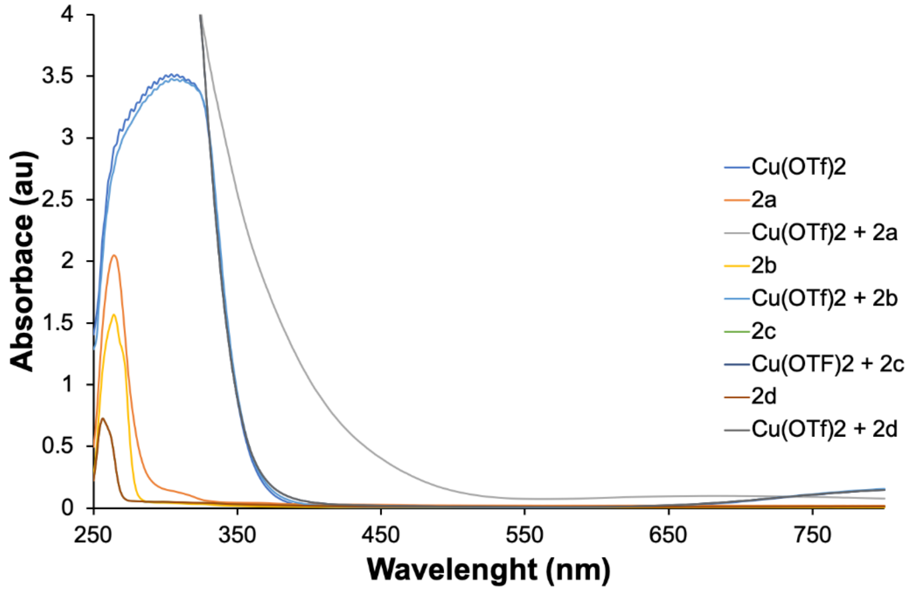

Figure S4.

8.1.3 SET Oxidation of 2a

Boronate complexes are electron rich species which can be oxidised. A ground state SET between **2a** and cumO_2TMS should be difficult (**Equation S1**).

$$\Delta G^{º} = F(E_{ox} - E_{red})$$

$$\Delta G^{º} = F\big(0.37\,V - (-1.61\,V)\big) = F(1.98\,V)$$

Equation S1.

Furthermore, **2a** proved stable in the presence of cumO_2TMS on the NMR time scale as demonstrated by ^{11}B NMR spectroscopy studies (**Figure S5**). However, we cannot exclude that over time a SET might take place.

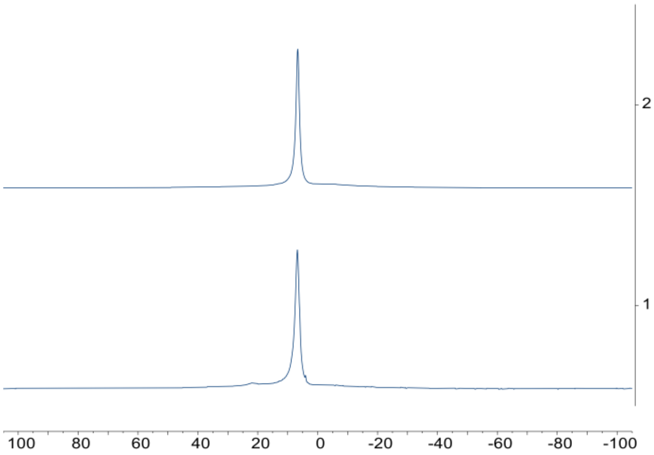

^{11}B NMR 2 = **2a**; ^{11}B NMR 1 = **2a** + cumOOTMS

Figure S5.

8.1.4 Probing α-Aminoalkyl Radical Generation by HAT

According to our proposed mechanism, the key α-aminoalkyl radical **B** is generated by HAT from the cumO• on Et_3N. To obtain more information on this step, we run competition experiments adding equimolar amount Hantzsch ester to the optimised reaction between **1** and **2a** (**Scheme S7**). As Hantzsch ester has significantly weaker C(*sp*^{3})–H bonds than Et_3N, it ought to undergo more facile HAT from cumO•. This should hamper α-aminoalkyl radical generation and therefore product formation.

As shown in **Scheme S6**, upon addition of Hantzsch ester the reactivity was completely suppressed and **3** was not observed.

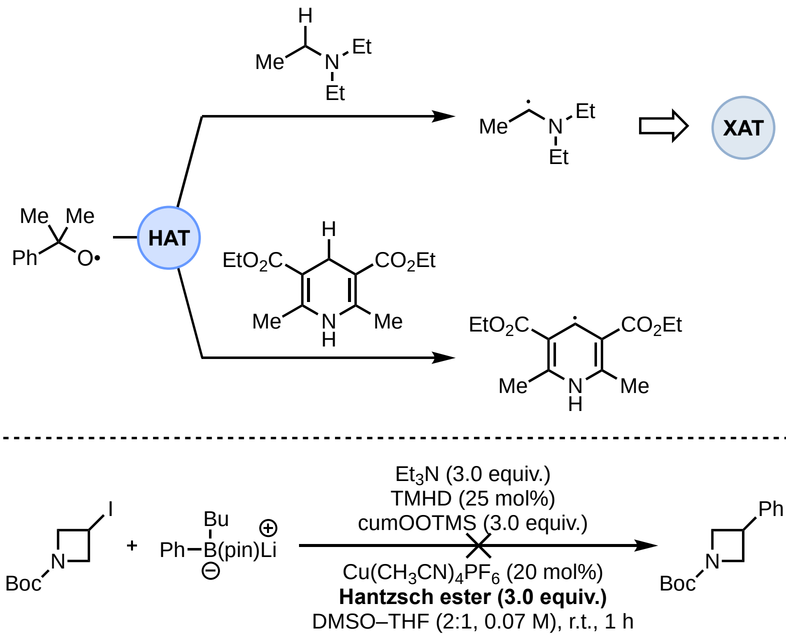

Scheme S6.

8.1.5 Probing α-Aminoalkyl Radical-Mediated XAT

To confirm the generation of α-aminoalkyl radicals and their involvement in the formation of the alkyl radical via XAT, we run the standard reaction between **1** and **2a** but replacing Et_3N with other amines that cannot lead to the formation of α-aminoalkyl radicals (**Scheme S7**).

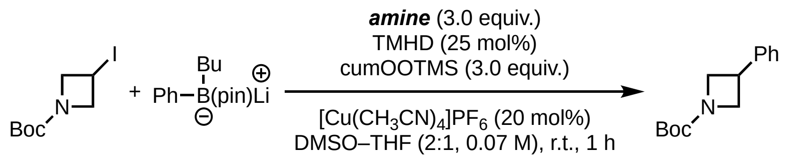

Scheme S7.

As shown in **Table S4**, the use of TMP and DABCO lead to formation of **3** in yield analogous to the experiment run in the absence of amine (compare entries 1 and 2 with 3). These results support the fact that with these two amines no α-aminoalkyl radical is generated and therefore low yielding reactivity might occur via the formation of either Ph• or Me•.

Table S4.

Entry	Amine	Yield (%)	**1** (%)
**1**	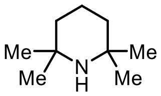	19	76
**2**	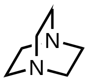	17	80
**3**	–	16	80

8.1.6 Ruling Out Direct SET Reduction of the Alkyl Iodide

Unactivated secondary alkyl iodides have a low reduction potential (E_{red} < –2 V vs SCE) that makes a direct SET with [Cu(I)] challenging. To support this hypothesis, we have attempted the reaction of **1** and **2a** using a stoichiometric [Cu(I)]. This experiment was performed by premixing **2a** with the [Cu(I)], followed by the addition of **1**.

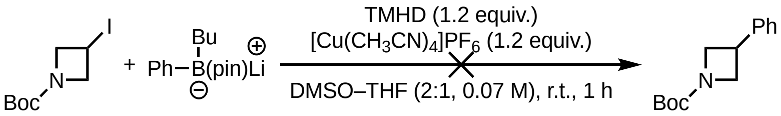

Scheme S8.

The fact that **3** was not formed and **1** was recovered in quantitative yield, confirm the inability of [Cu(I)] to undergo ground-state SET with **2a** as well as direct oxidative addition.

8.1.7 Probing Radical Capture by [Cu(II)]–Ar Species as C(sp^3)–C(sp^2) Bond Forming Step

To obtain more information supporting the capture of the alkyl radical **C** by the Ph–[Cu(II)] species **A** to give a Ph,alkyl–[Cu(III)] intermediated **D** followed by reductive elimination to **3**, we studied the reaction between a pre-formed [Cu(II)]–Ph complex [prepared by premixing **2a** with $Cu(OTf)_2$] with lauroyl peroxide **4** (**Scheme S9**). As **4** undergoes thermal O–O bond homolysis,[24] the alkyl radical formation does not require SET or XAT activation. The desired product **5** was observed in 38% yield and this supports our proposed pathway for C(sp^3)–C(sp^2) bond assembly.

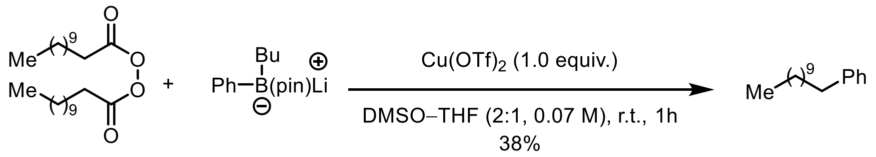

Scheme S9

8.2 Alternative Mechanism

We also considered an alternative mechanism based on transmetalation of **2a** onto [Cu(I)] followed by oxidation to [Cu(II)]–Ph (**Scheme S10**). However, we have not been able to obtain mechanistic evidence on the key group state SET (see below).

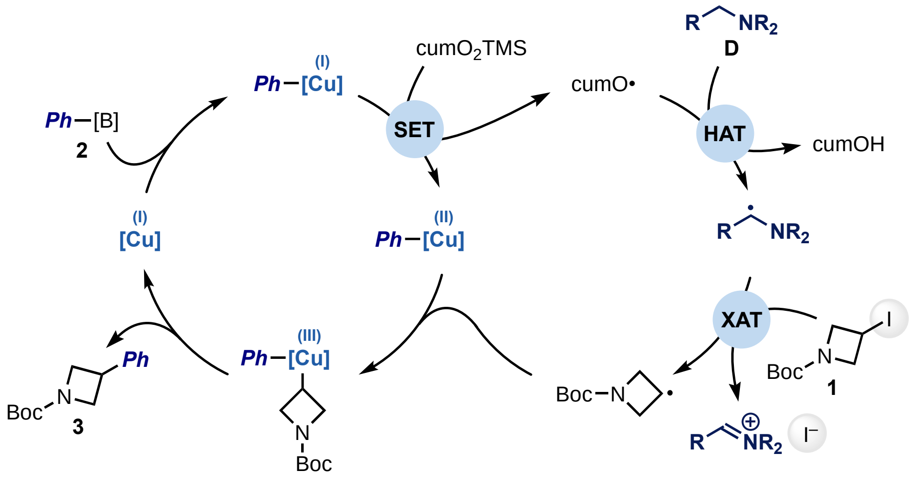

Scheme S10.

8.2.1 Coordination of 2a to [Cu(I)] to Give [Cu(I)]–Ph

In this proposed mechanism, the boronate **2a** undergoes transmetalation with [Cu(I)], forming the Ph–[Cu(I)] complex **A**. We have evaluated the transmetalation by UV/Vis absorption spectroscopy and observed a strong change in the absorption profile (**Figure S6**).

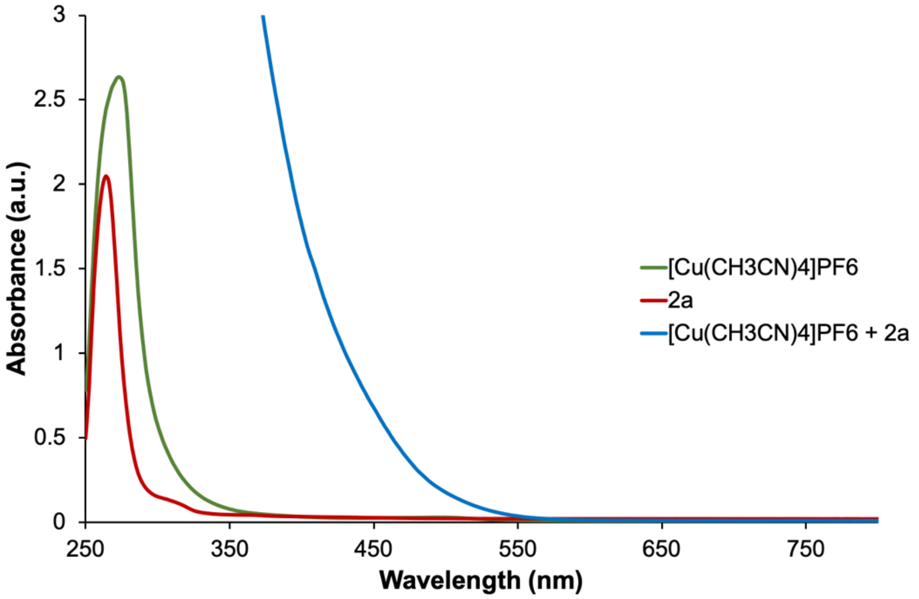

Figure S6.

8.2.2 Oxidation of [Cu(I)]–Ph by cumO_2TMS to [Cu(II)]–Ph

We have tried to follow the reaction of **A** with cumOOTMS but we did not succeed in observing the formation of a [Cu(II)] species which should display a characteristic absorption at 750 nm by UV/Vis absorption spectroscopy analysis.

9 Cyclic Voltammetry Studies

Cyclic voltammetry was conducted on an EmStat (PalmSens) potentiostat using a 3-electrodes cell configuration. A glassy carbon working electrode was employed alongside a platinum wire counter electrode and a Ag/AgCl reference electrode. Solution was degassed by bubbling N_2 prior to measurements. 5 mM solution of the the boronate **2a** was freshly prepared in dry acetonitrile along with 0.1 M of tetrabutylammonium hexafluorophosphate as supporting electrolyte and was examined at a scan rate of 0.1 Vs^{-1}. Decamethylferrocene ($E_{1/2}$ = −0.125 V vs SCE) was added at the end of the measurement as an internal standard to determine the precise potential scale. [39] Potential values are given versus the saturated calomel electrode (SCE). Irreversible wave was obtained; therefore, the potentials was estimated at half the maximum the maximum current, as previously in the literature (**Figure S7**).[40]

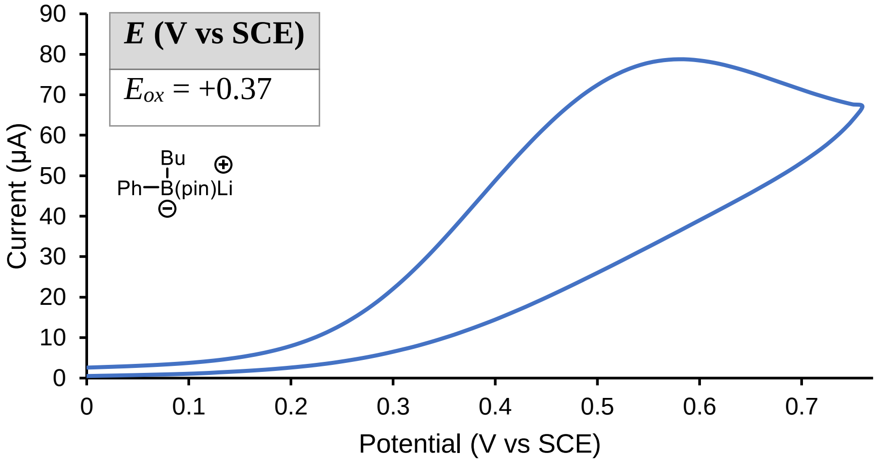

Figure S7.

10 NMR Spectra

S1 - ^{1}H NMR (400 MHz, $CDCl_3$)

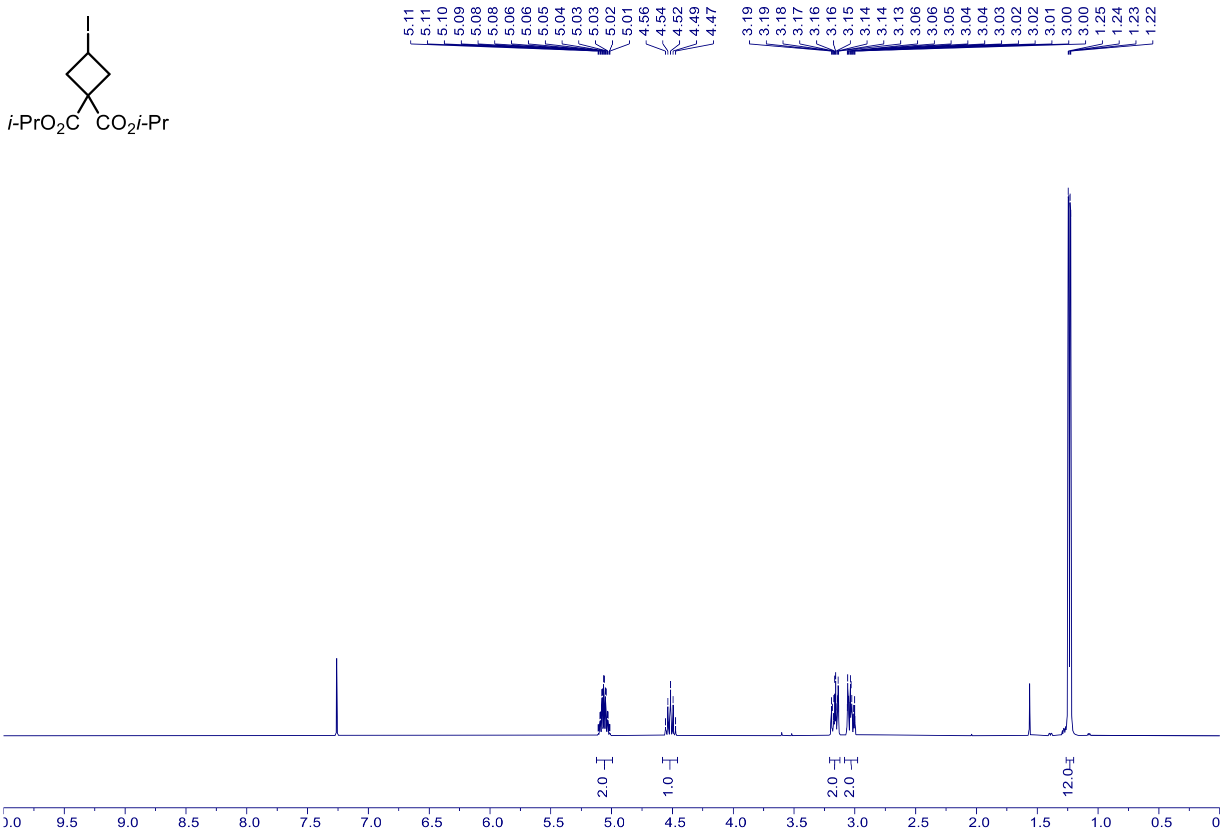

S1 - ^{13}C NMR (101 MHz, $CDCl_3$)

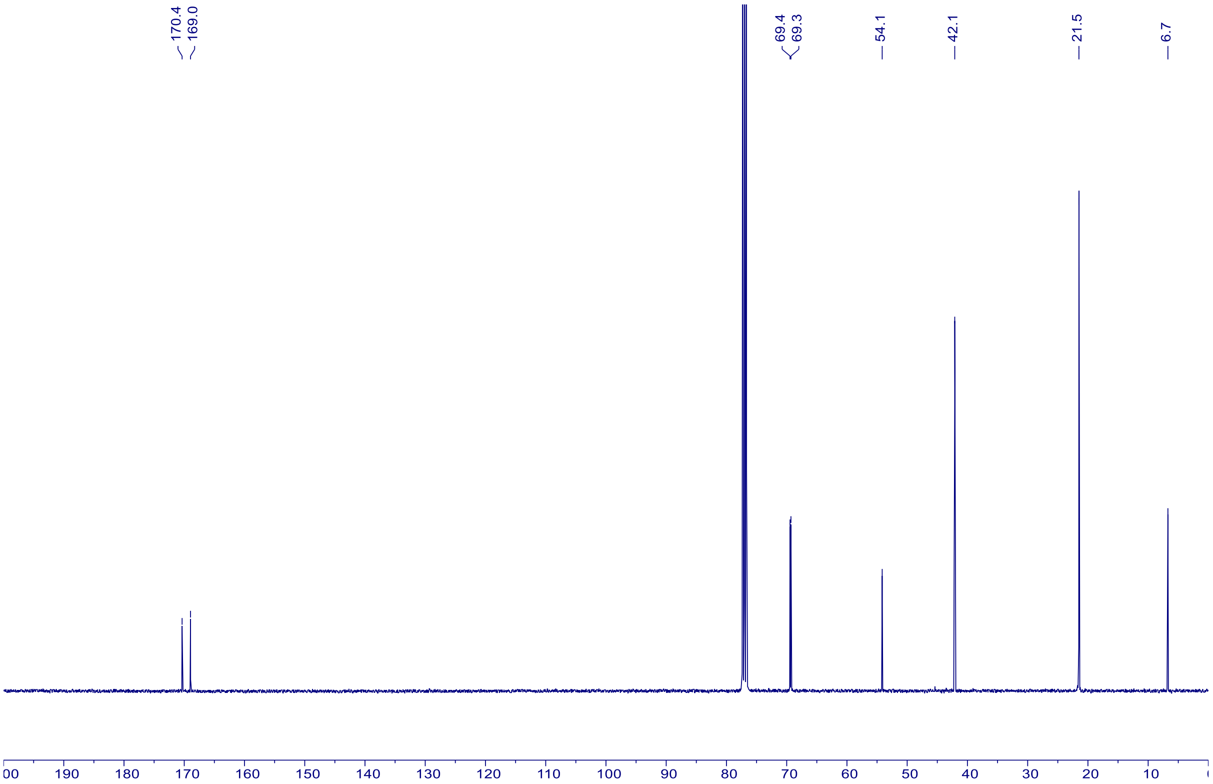

S17 - ^{1}H NMR (400 MHz, $CDCl_3$)

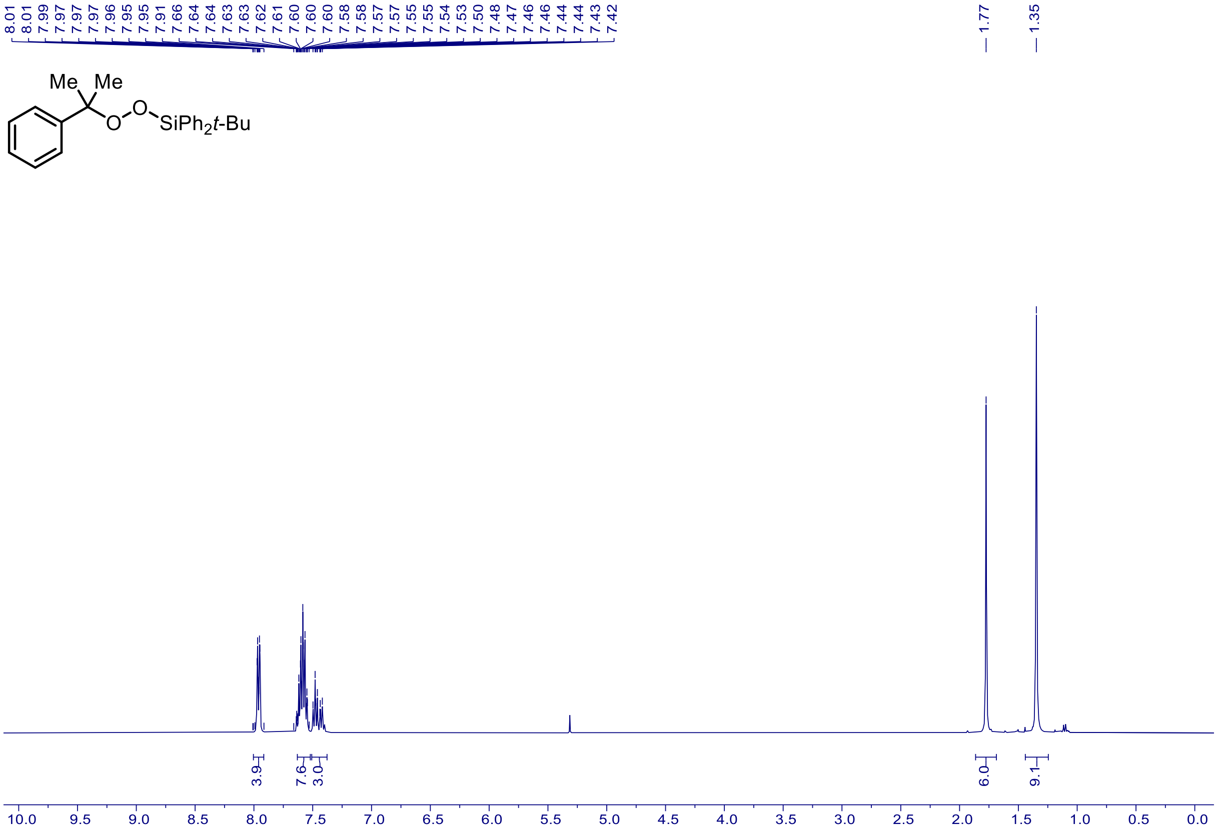

S17 - ^{13}C NMR (101 MHz, $CDCl_3$)

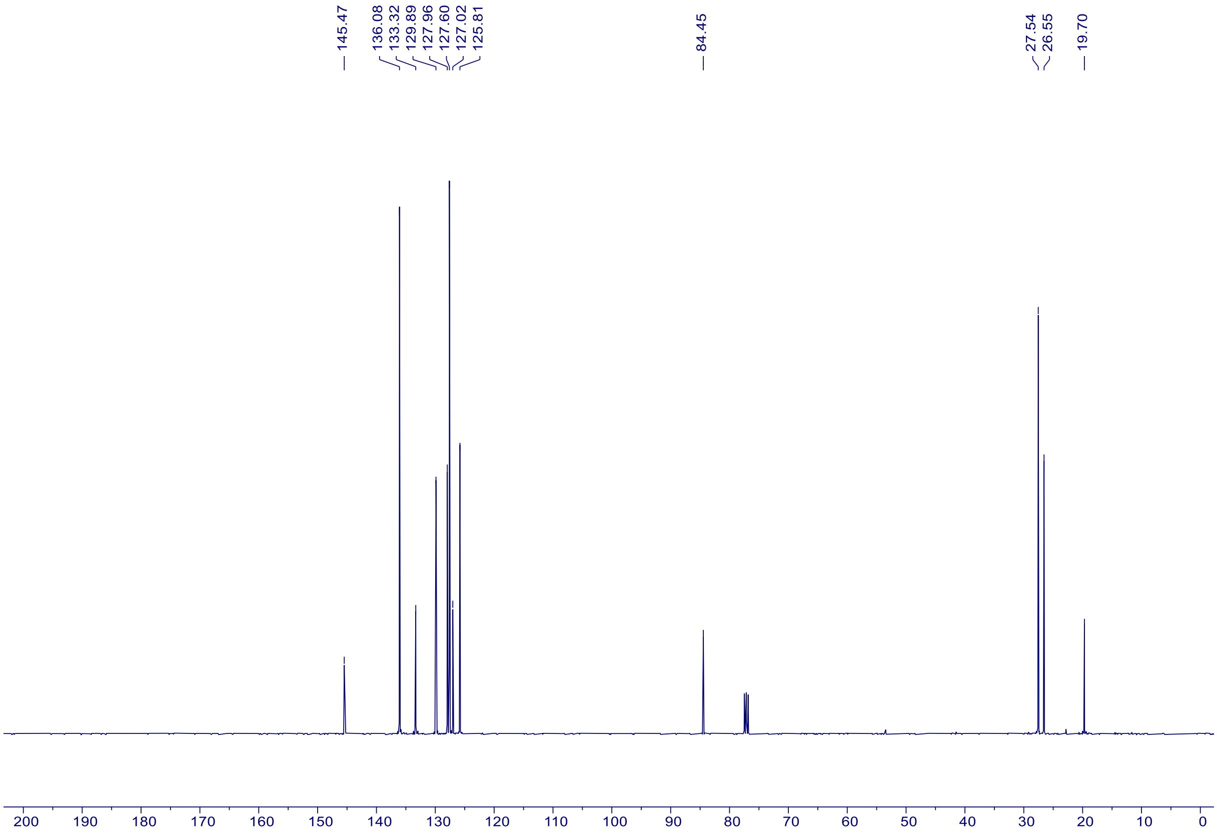

8- ^{1}H NMR (400 MHz, $CDCl_3$)

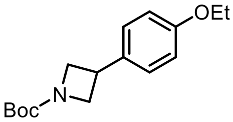

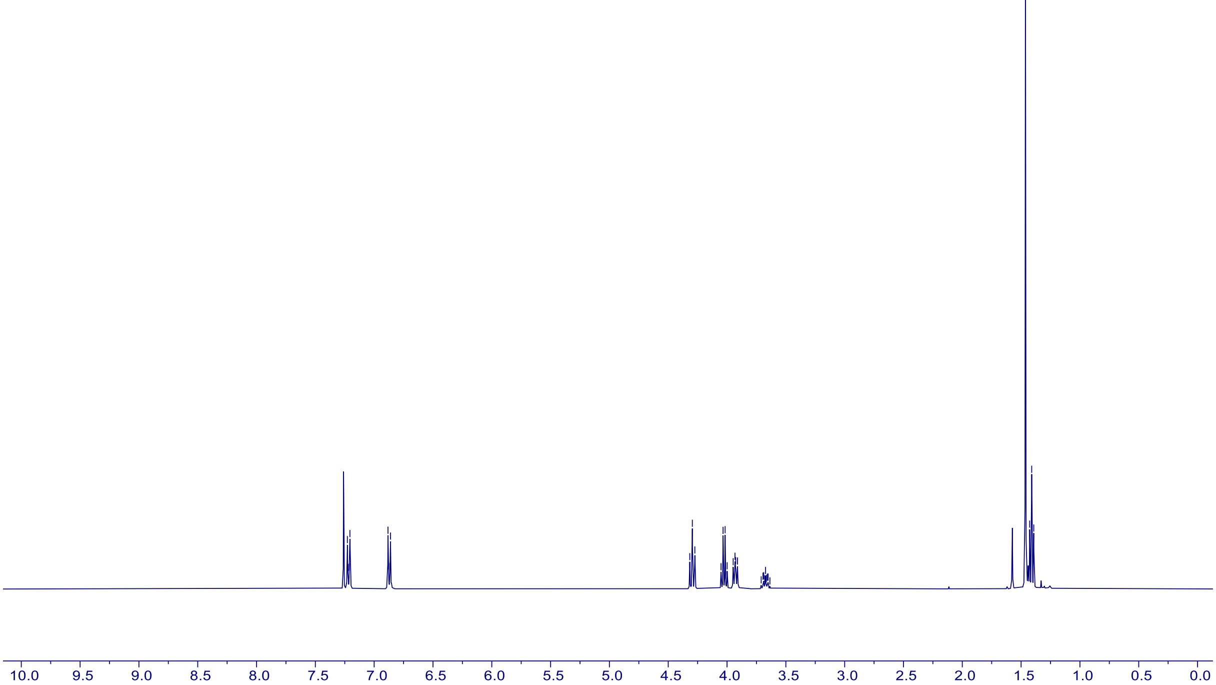

8 - ^{13}C NMR (101 MHz, $CDCl_3$)

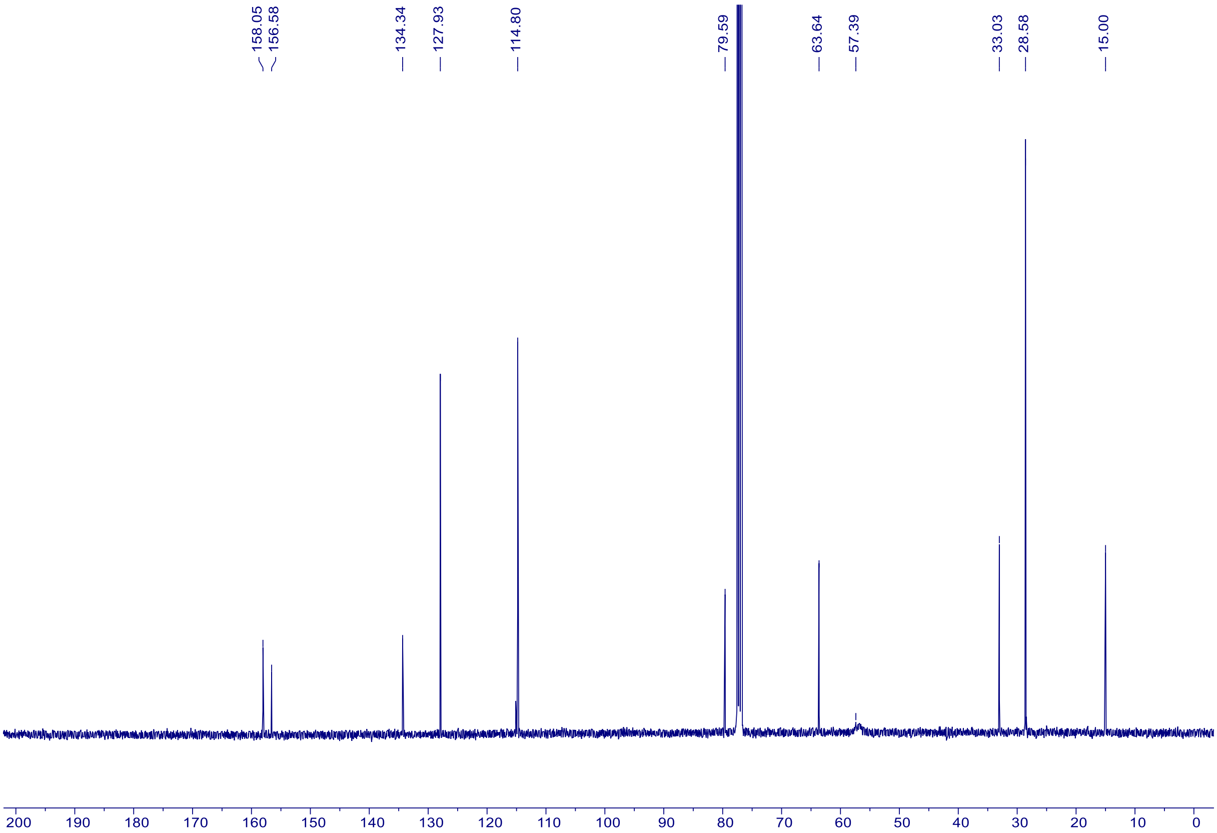

16 - ^{1}H NMR (500 MHz, $CDCl_3$)

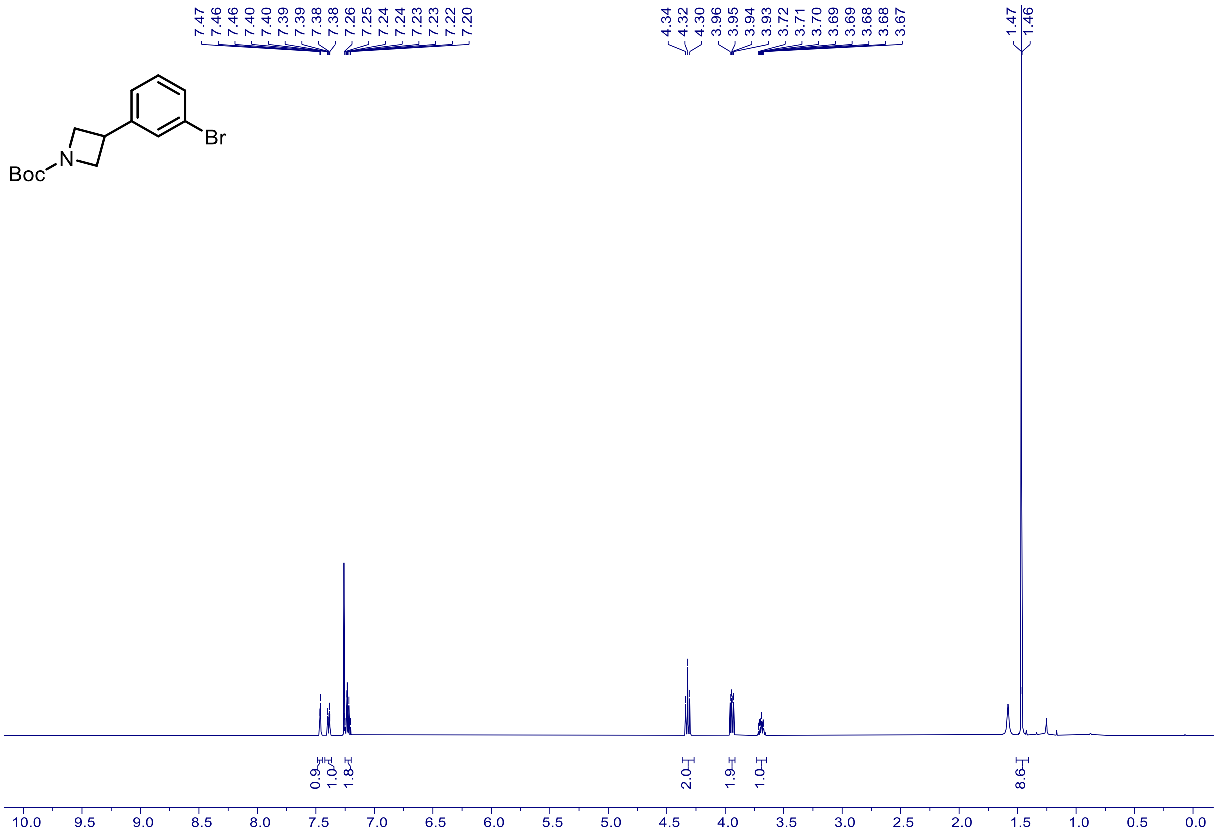

16 - ^{13}C NMR (126 MHz, $CDCl_3$)

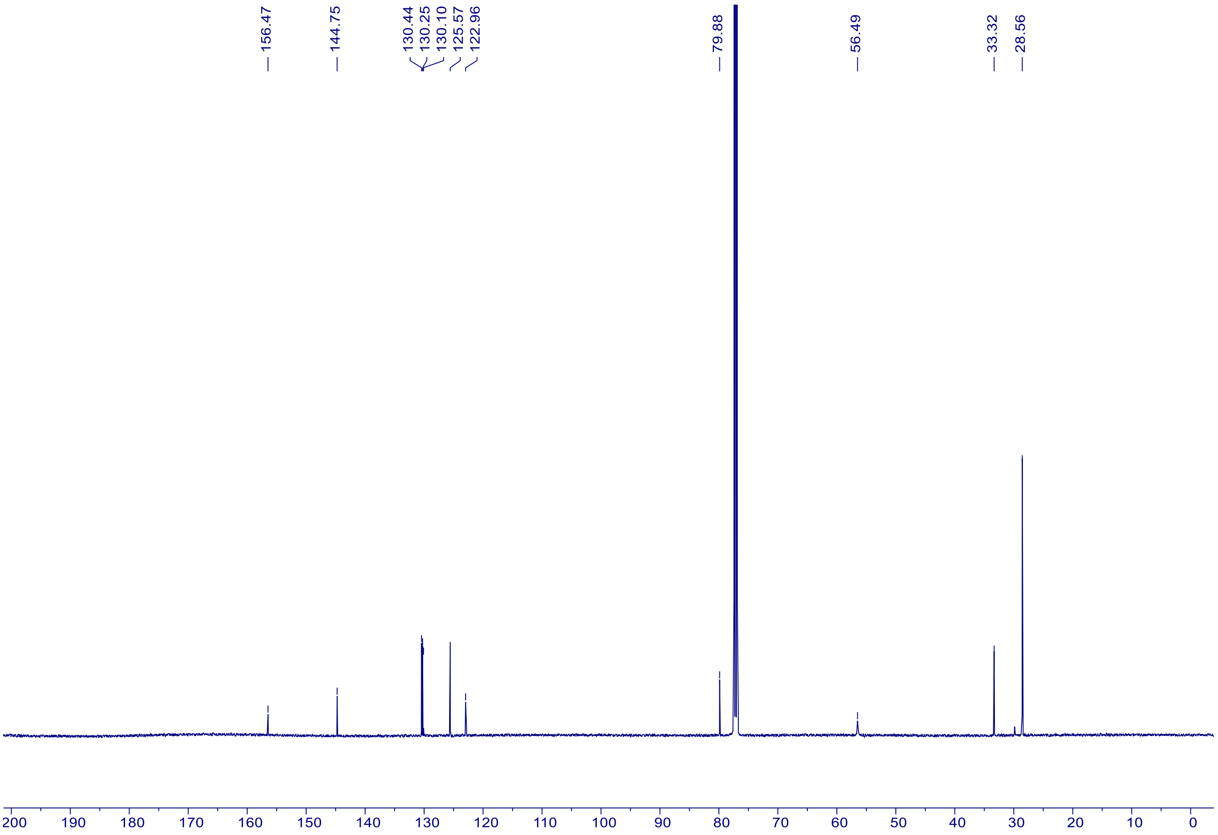

19 - ^{1}H NMR (500 MHz, $CDCl_3$)

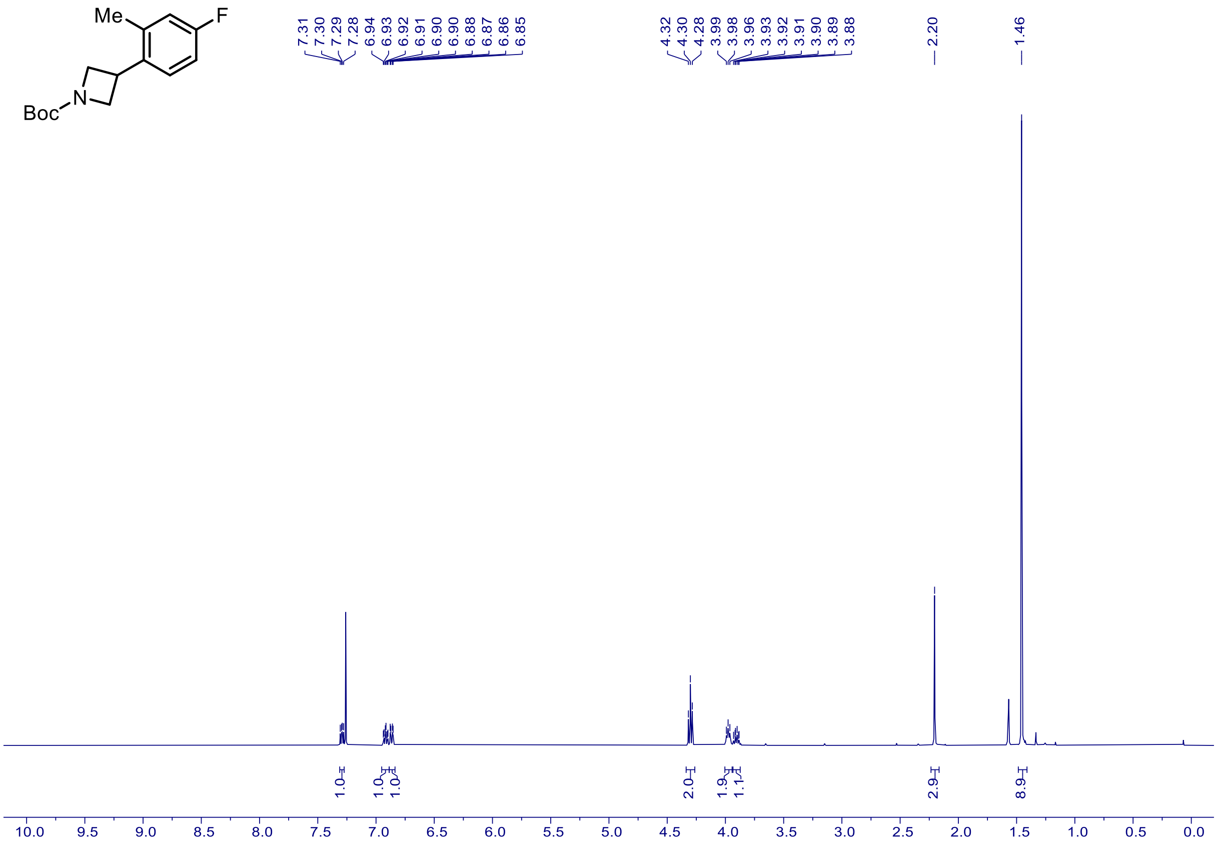

19 - ^{13}C NMR (126 MHz, $CDCl_3$)

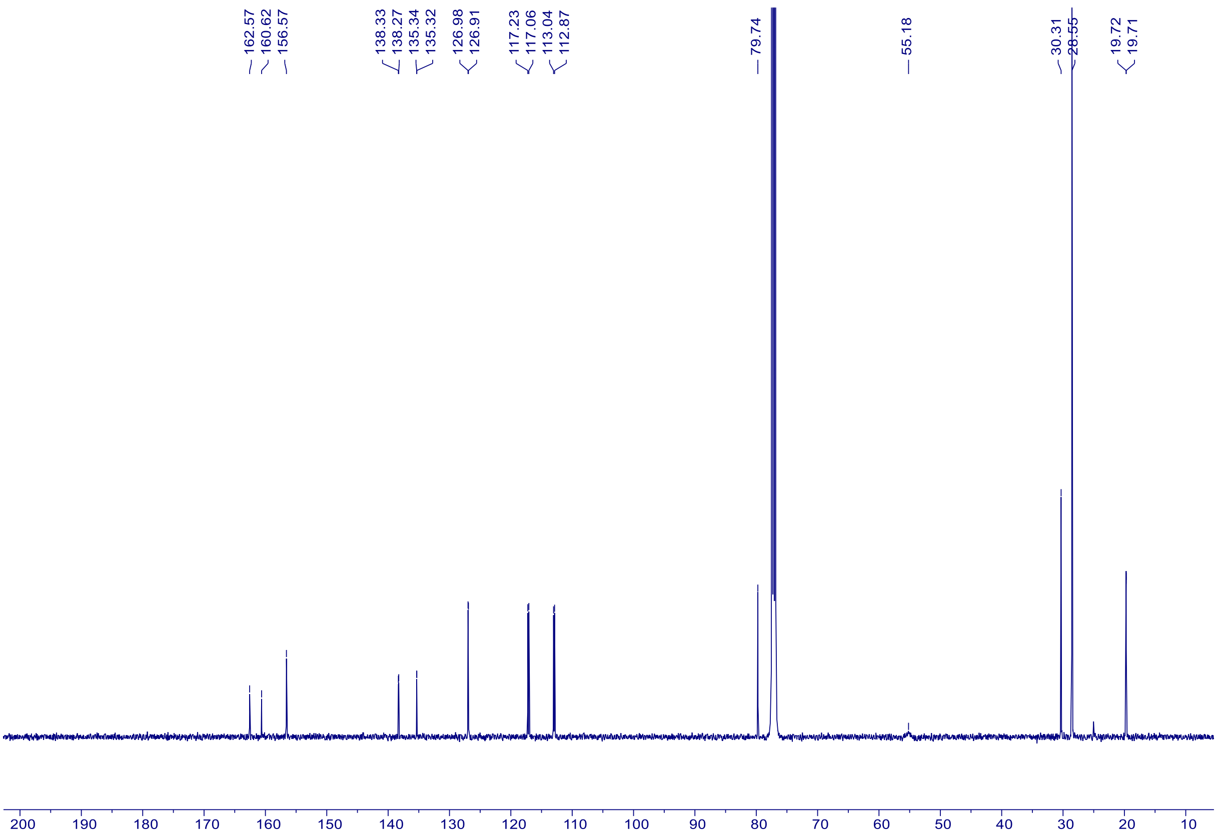

19 - ^{19}F NMR (376 MHz, $CDCl_3$)

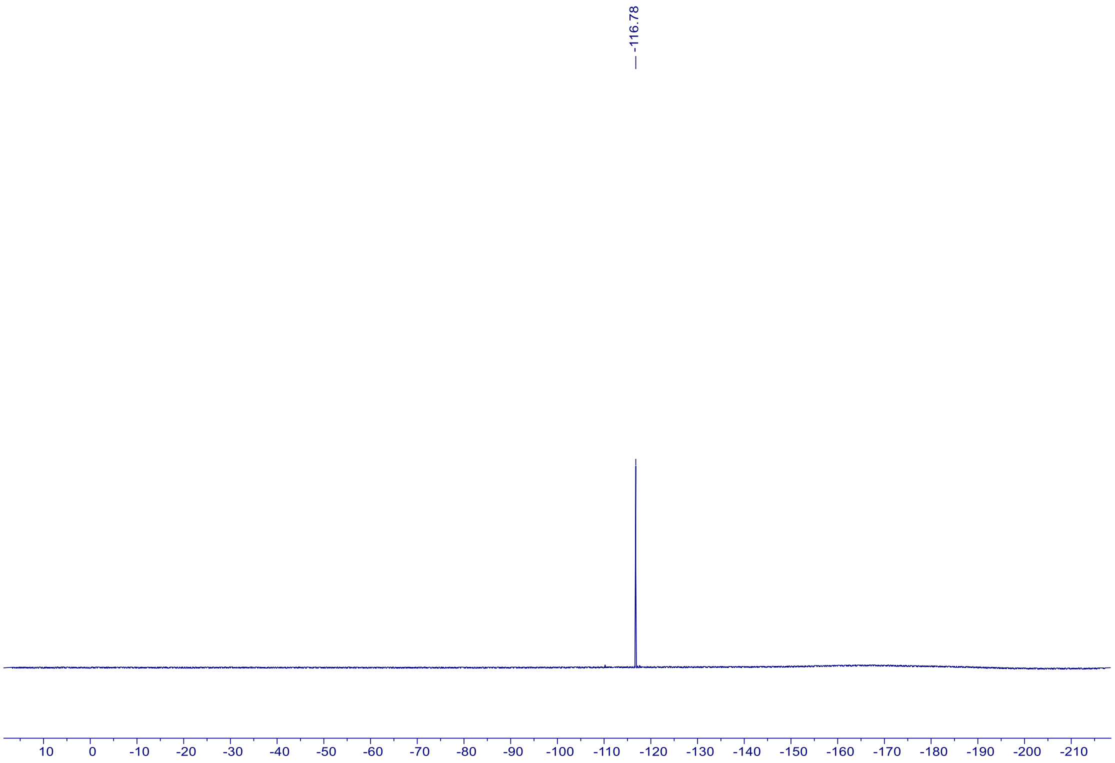

21 - ^{1}H NMR (400 MHz, $CDCl_3$)

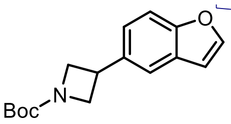

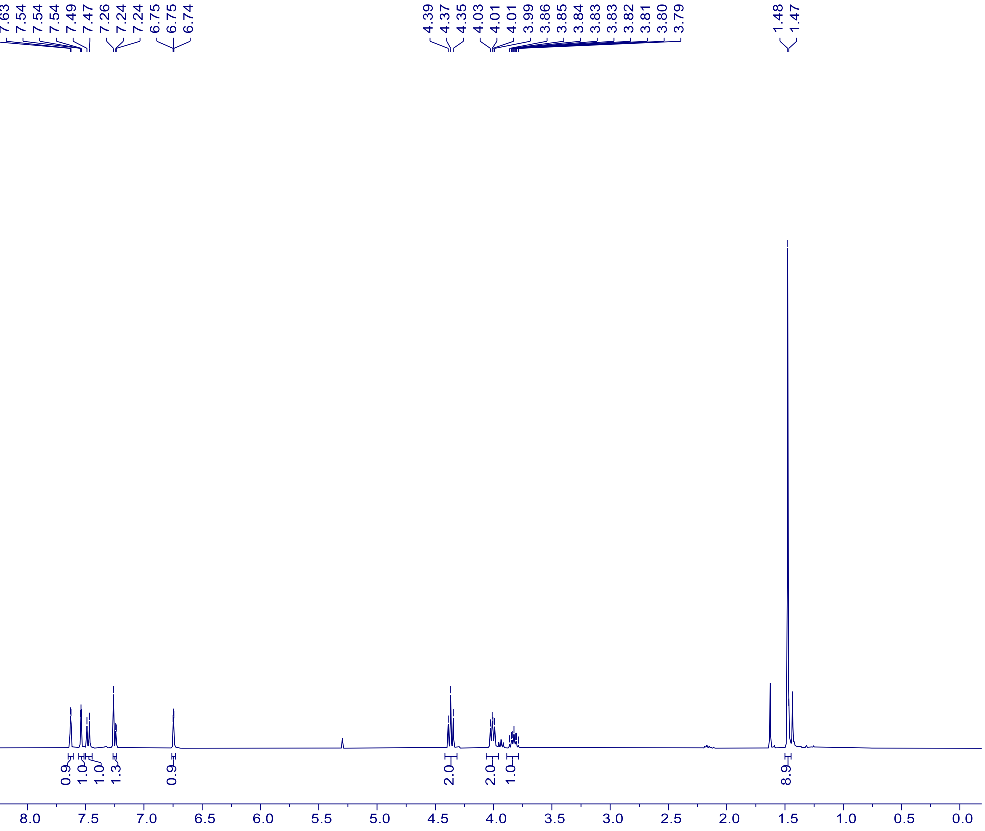

21 - ^{13}C NMR (101 MHz, $CDCl_3$)

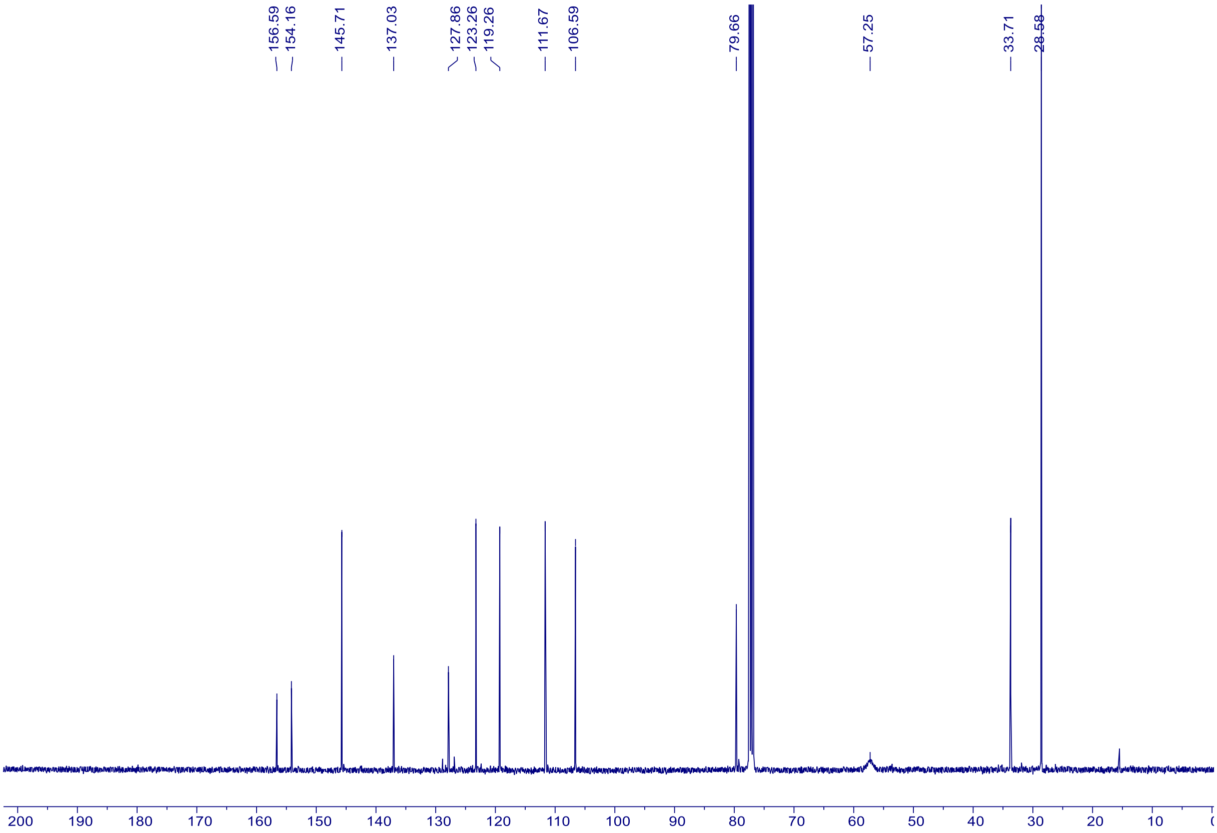

23 - ^{1}H NMR (500 MHz, $CDCl_3$)

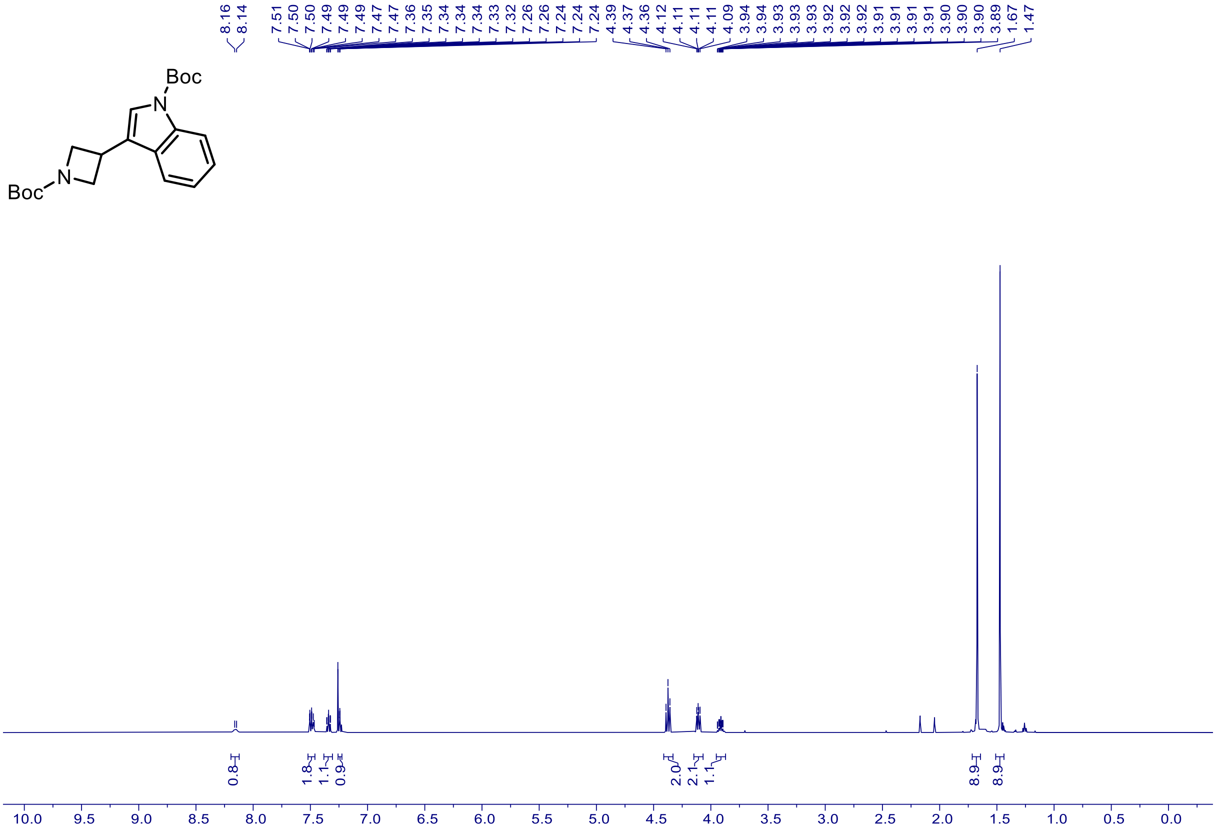

23 - ^{13}C NMR (126 MHz, $CDCl_3$)

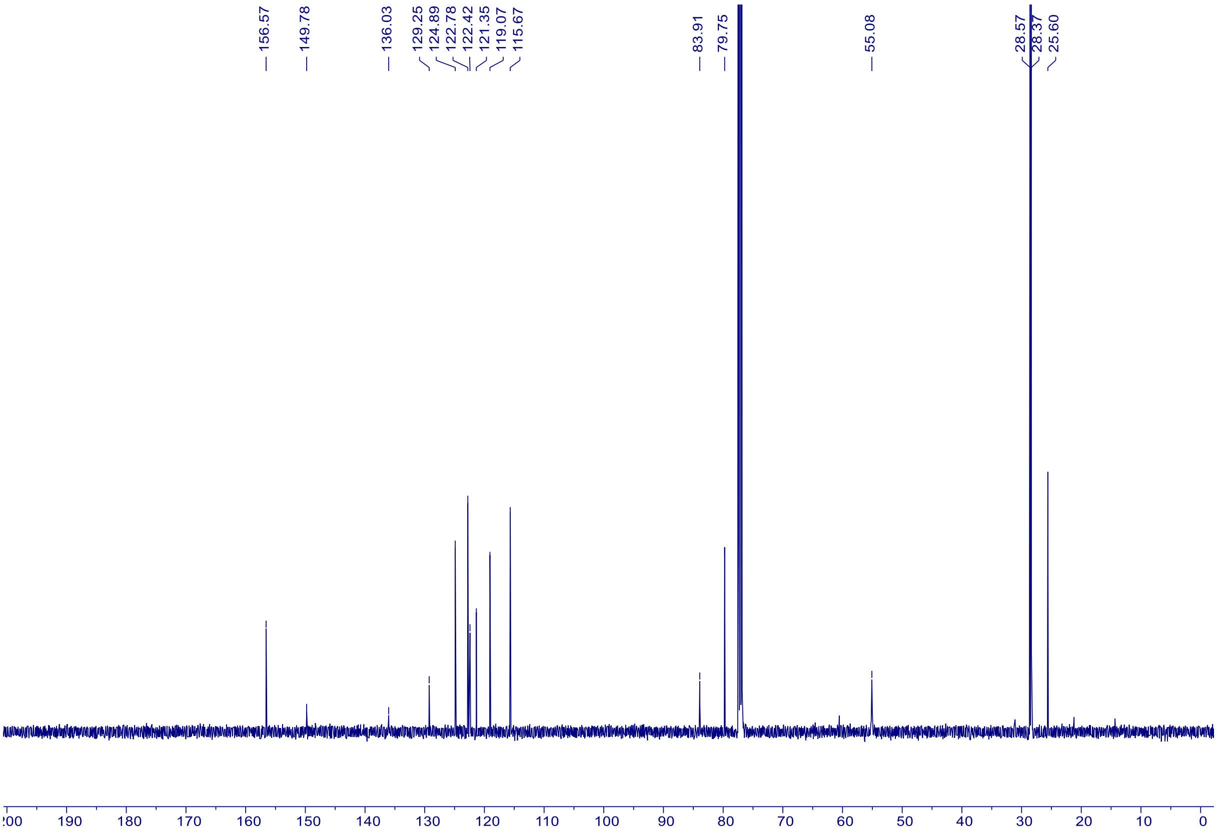

24 - ^{1}H NMR (500 MHz, $CDCl_3$)

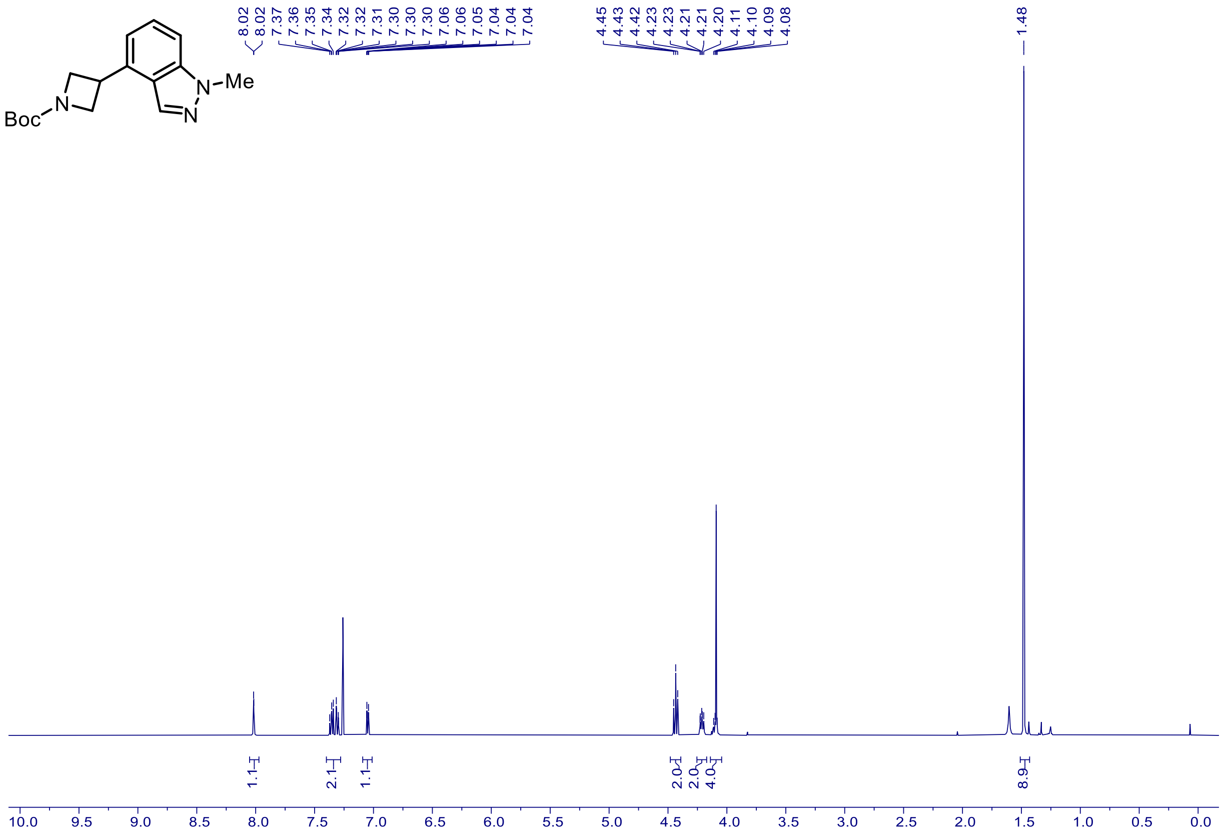

24 - ^{13}C NMR (126 MHz, $CDCl_3$)

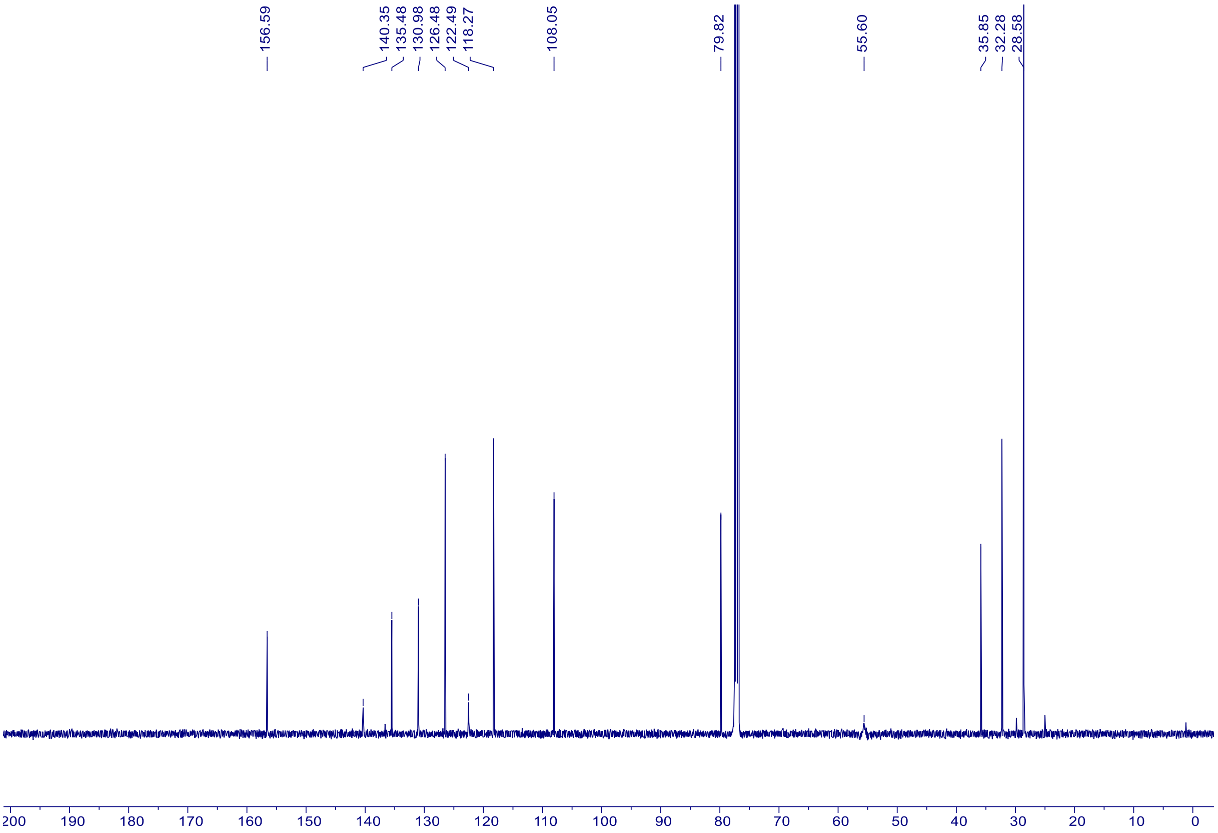

27 - ^{1}H NMR (400 MHz, $CDCl_3$)

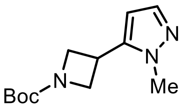

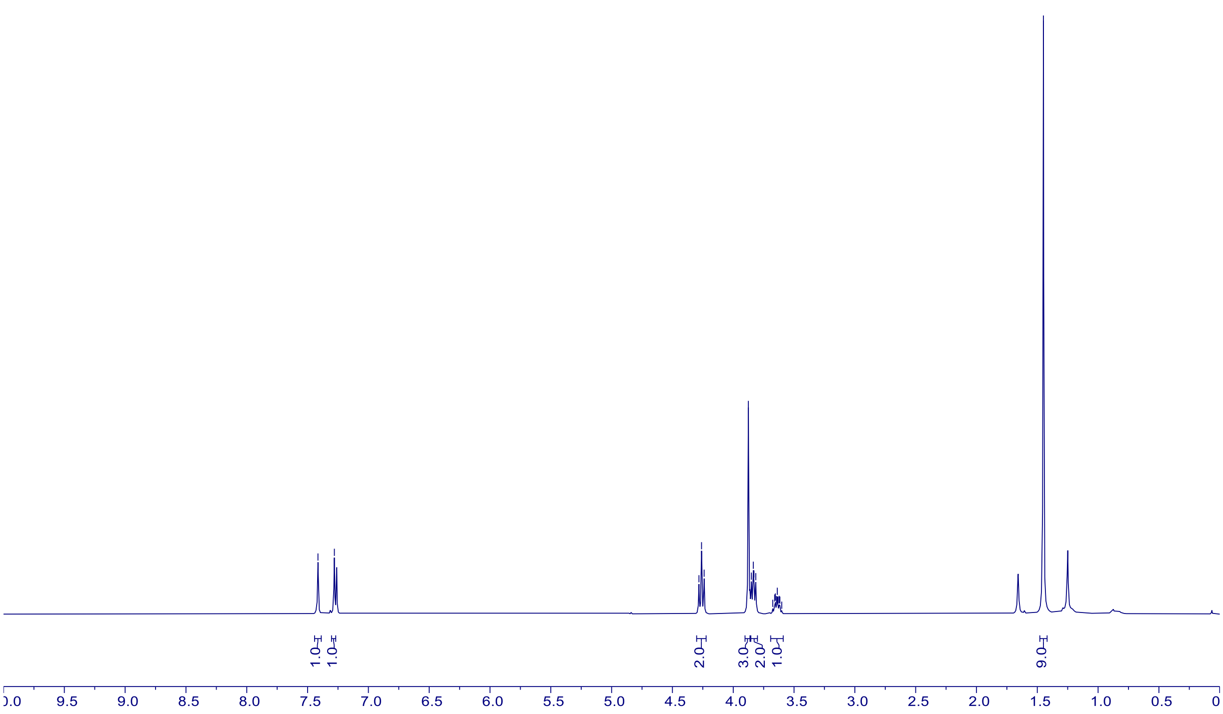

27 - ^{13}C NMR (101 MHz, $CDCl_3$)

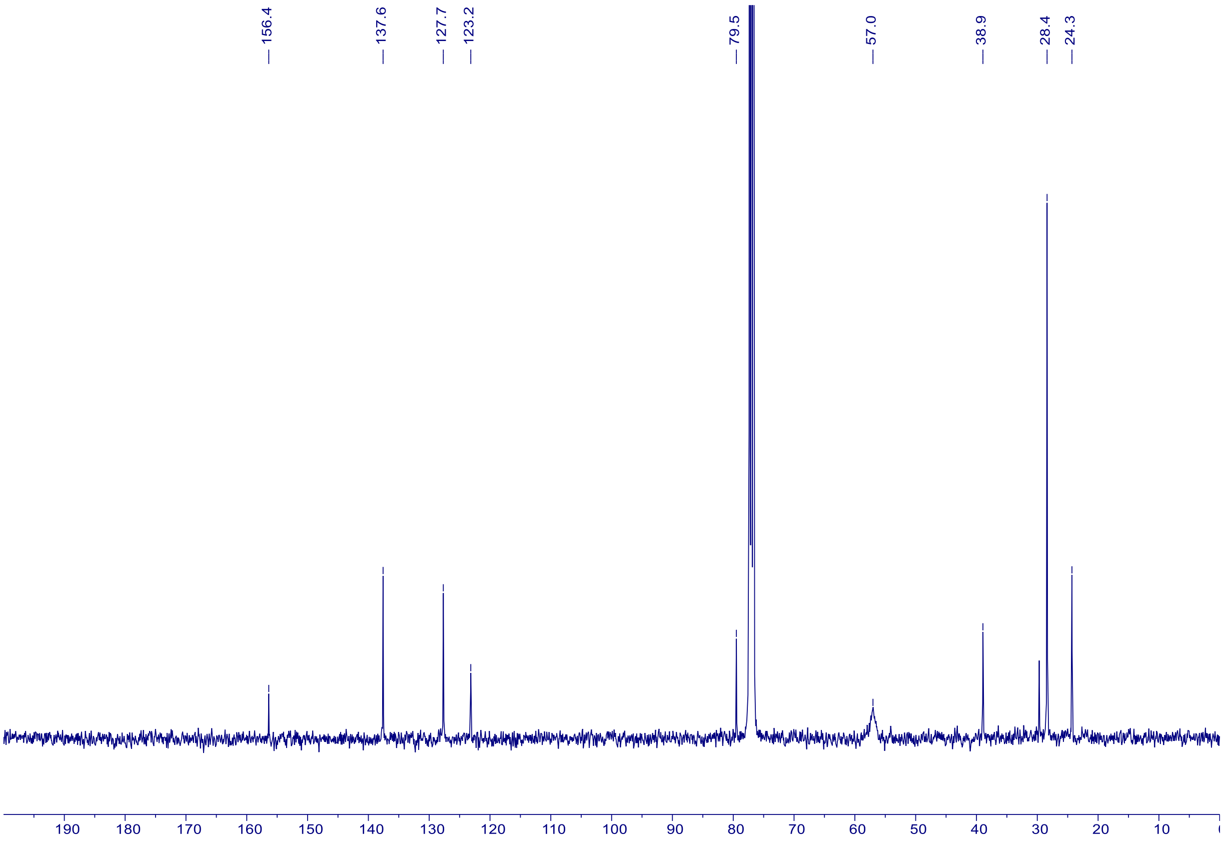

28 - ^{1}H NMR (500 MHz, $CDCl_3$)

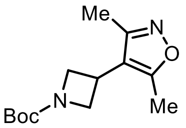

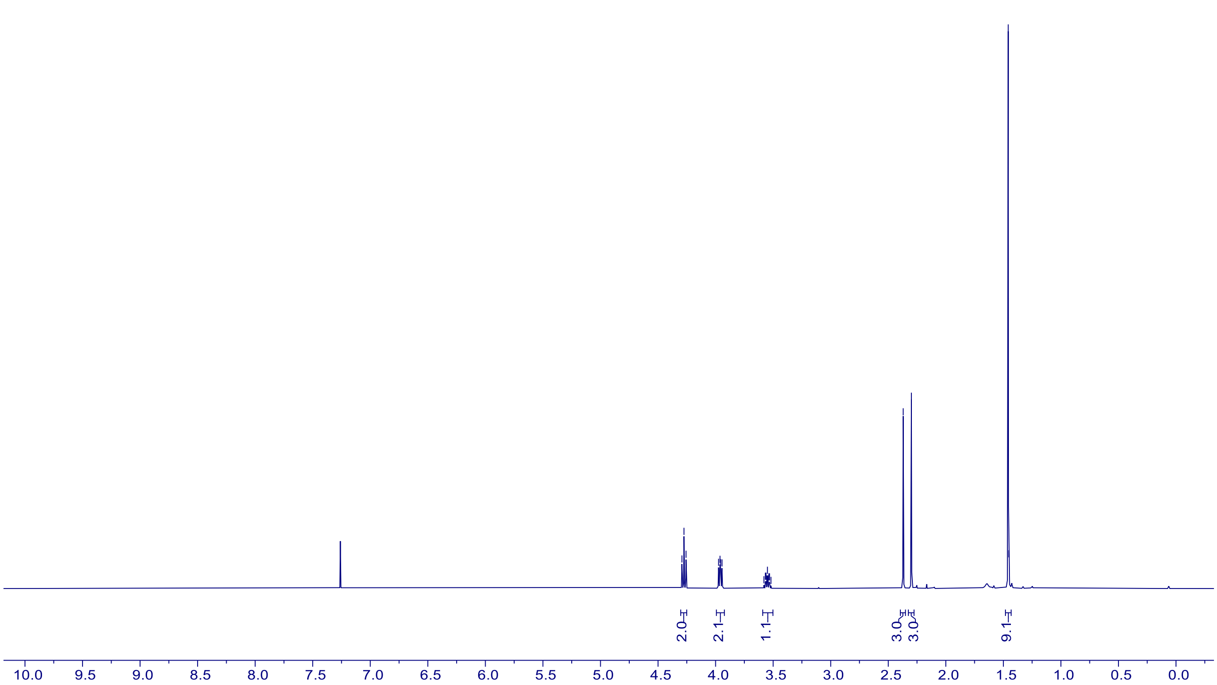

28 - ^{13}C NMR (126 MHz, $CDCl_3$)

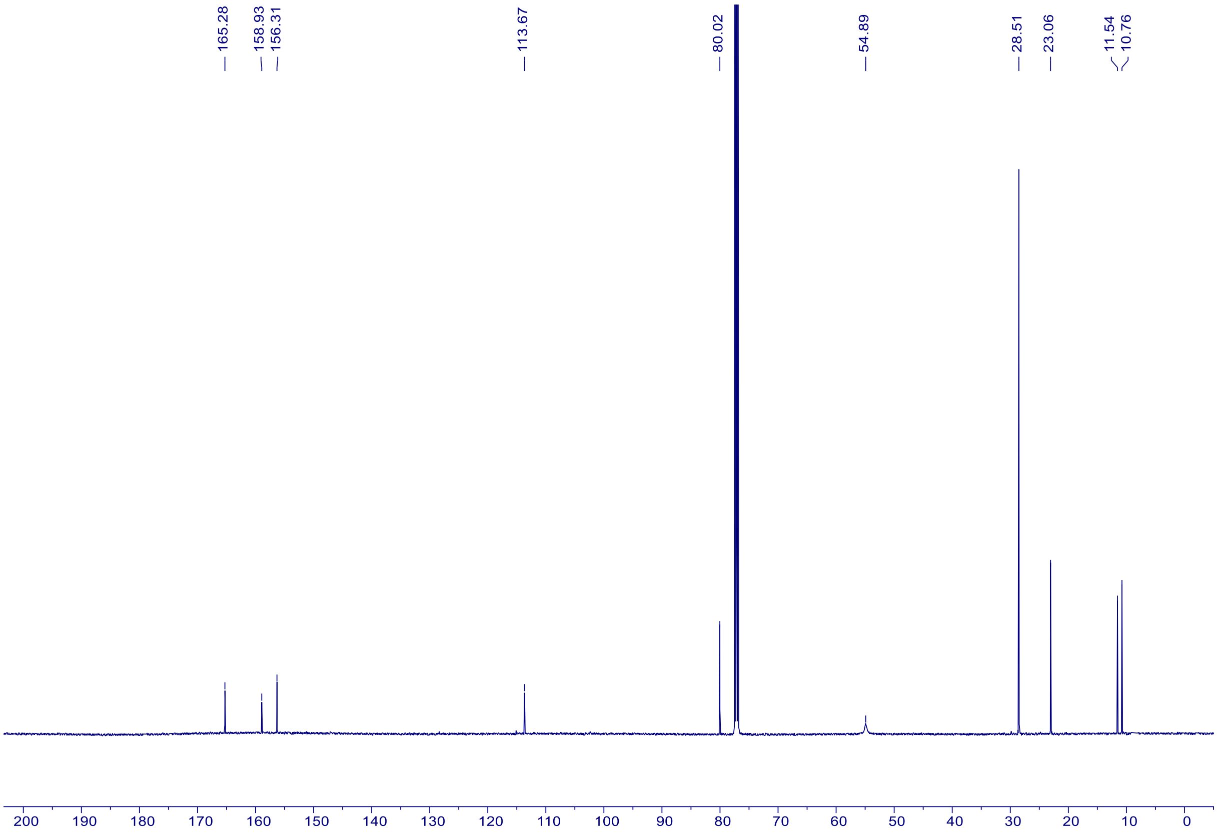

32 - ^{1}H NMR (500 MHz, $CDCl_3$)

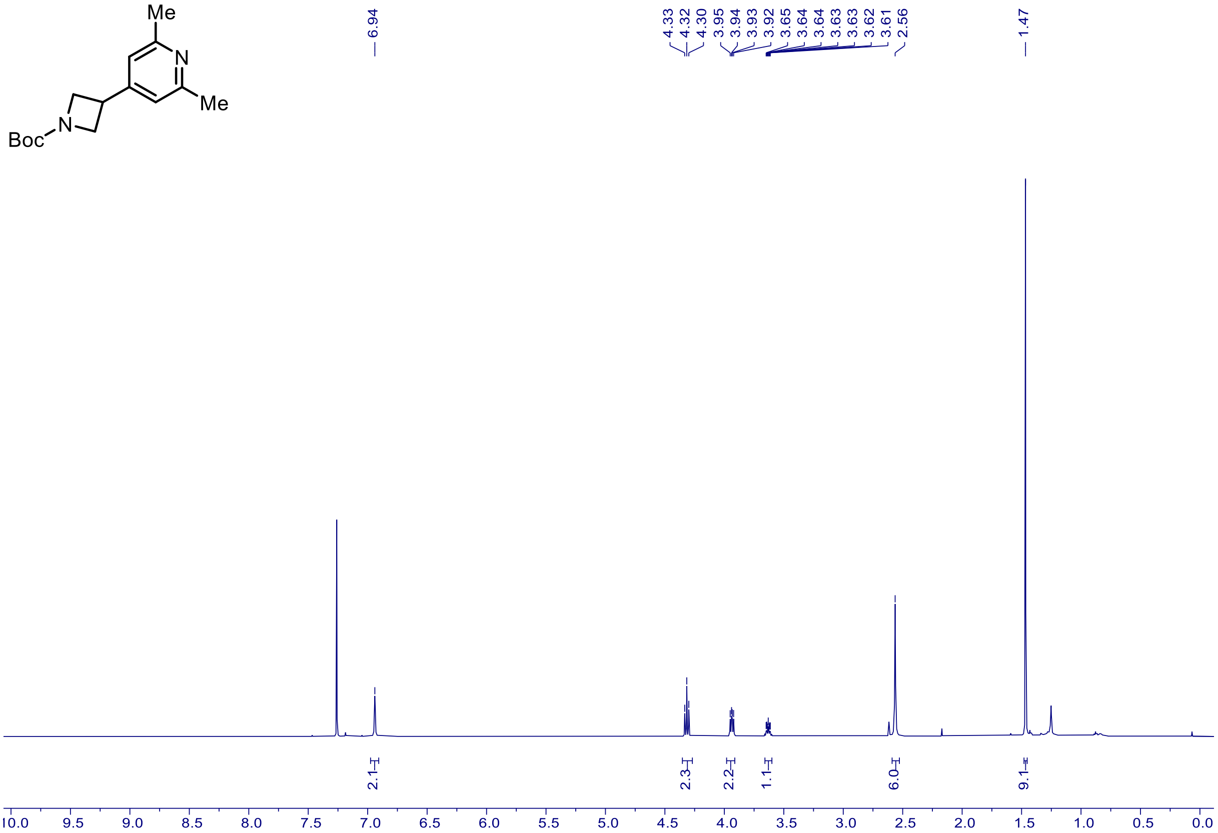

32 - ^{13}C NMR (126 MHz, $CDCl_3$)

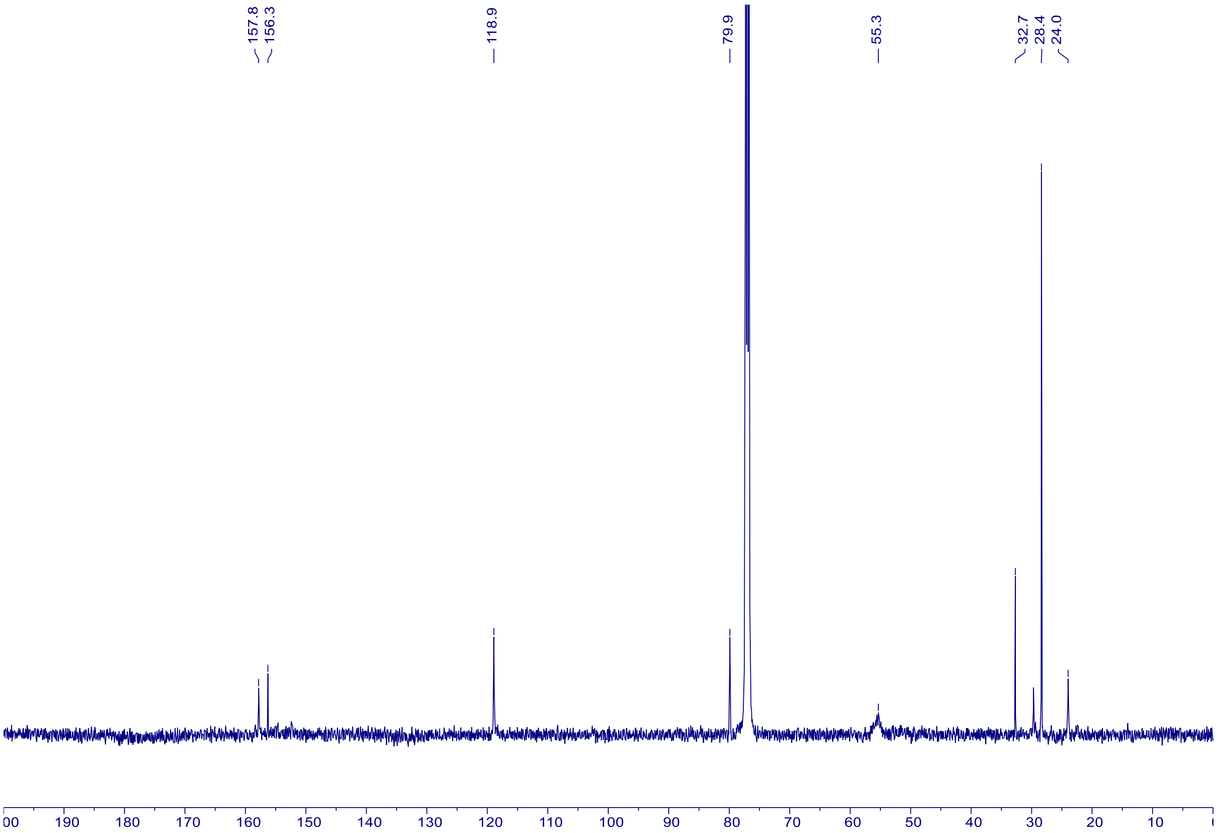

33 - ^{1}H NMR (500 MHz, $CDCl_3$)

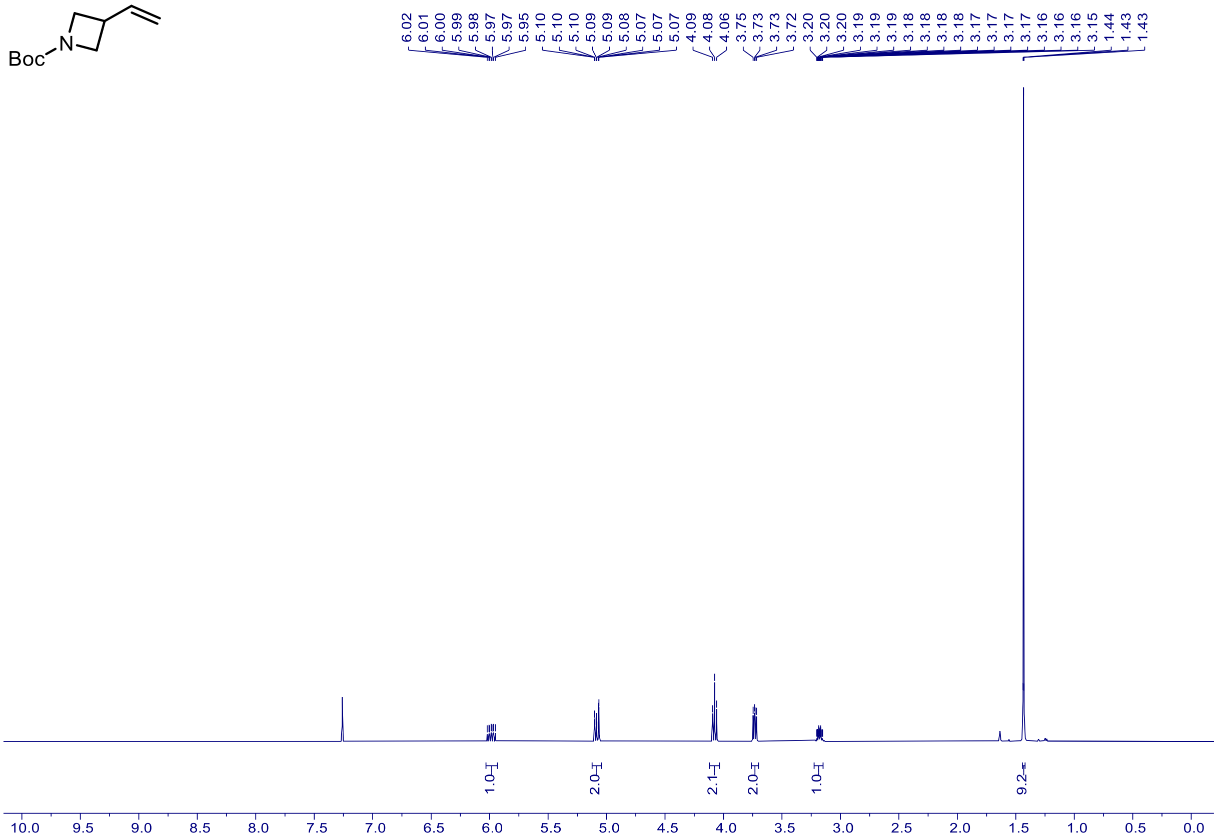

33 - ^{13}C NMR (126 MHz, $CDCl_3$)

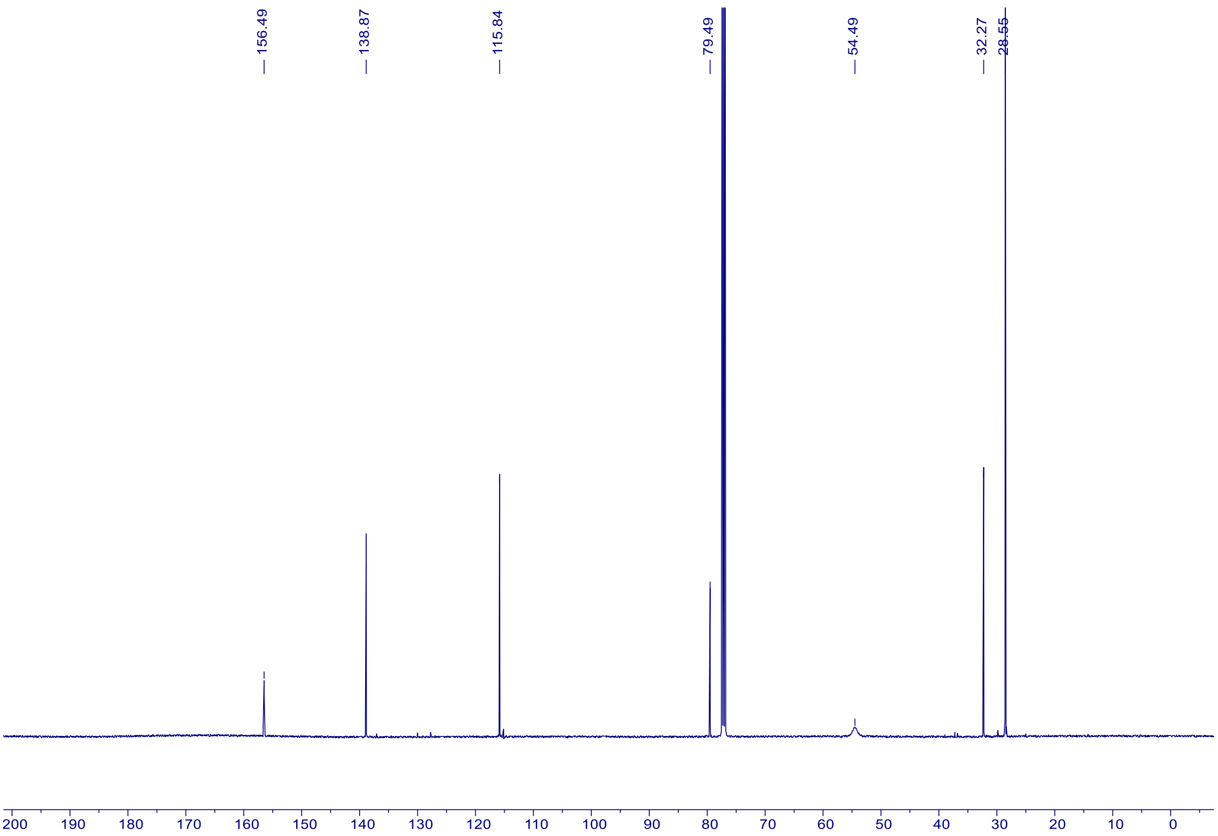

36 - ^{1}H NMR (400 MHz, CDCl$_3$)

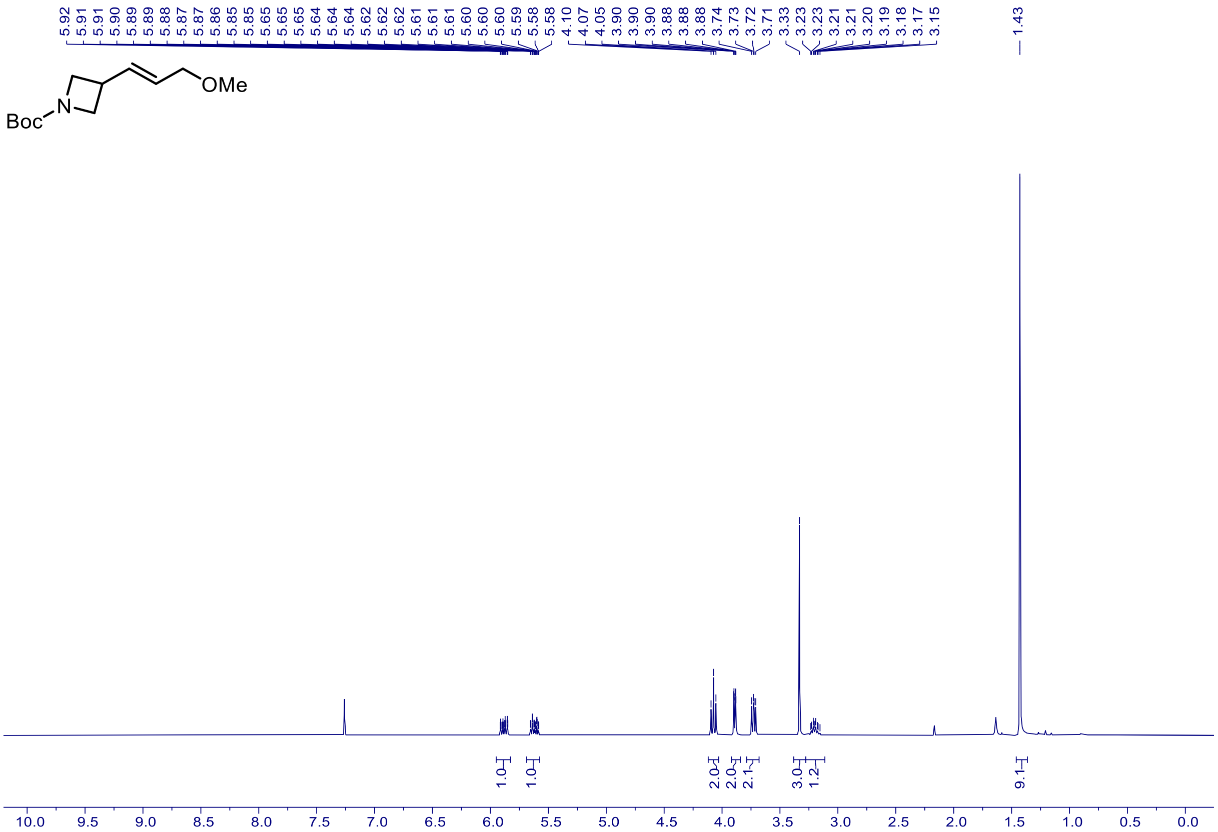

36 - ^{13}C NMR (101 MHz, CDCl$_3$)

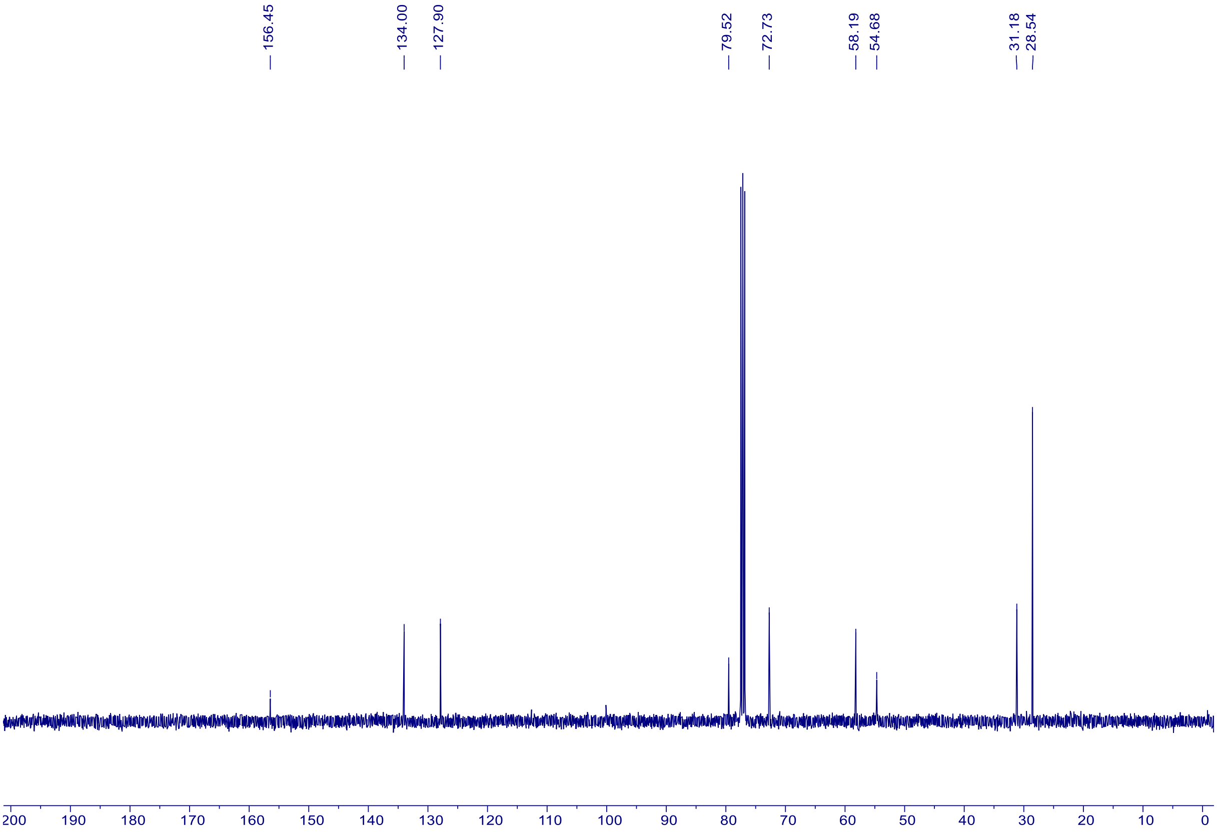

37 - ^{1}H NMR (500 MHz, $CDCl_3$)

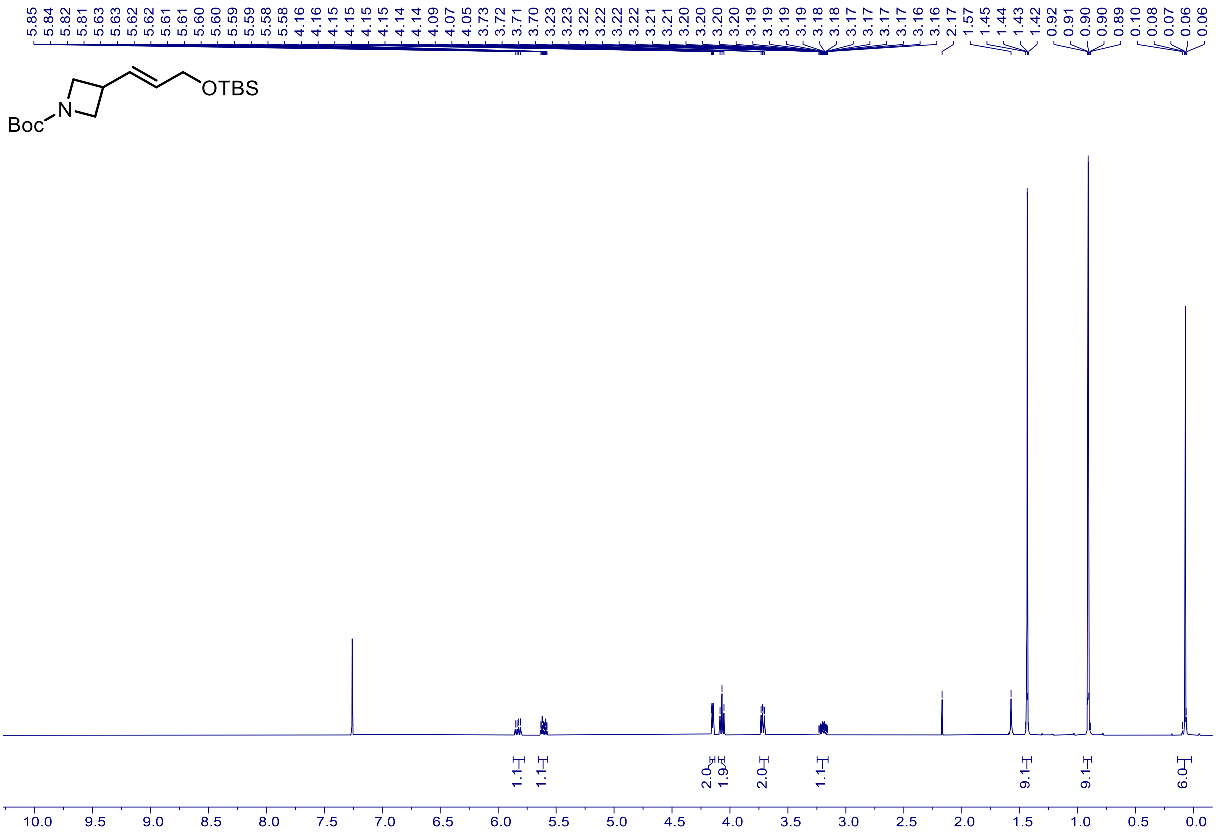

37 - ^{13}C NMR (126 MHz, $CDCl_3$)

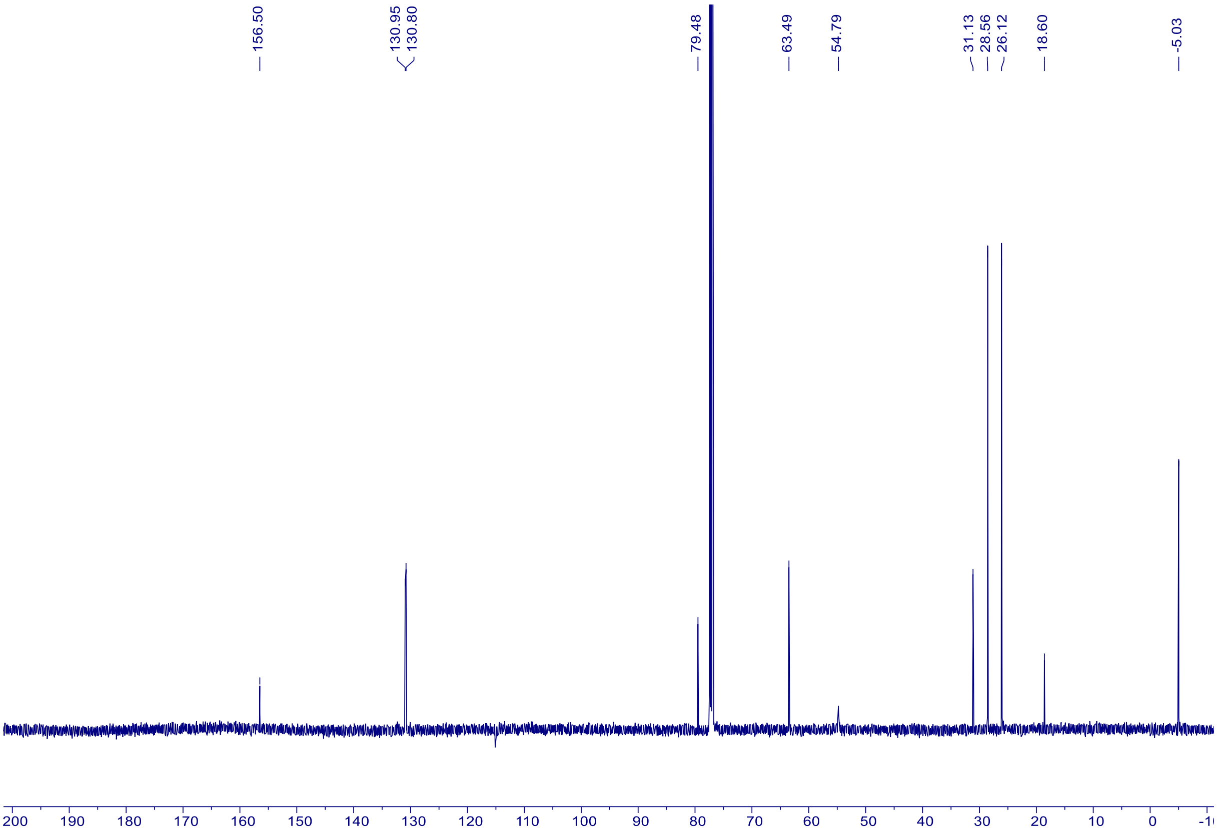

38 - ^{1}H NMR (500 MHz, $CDCl_3$)

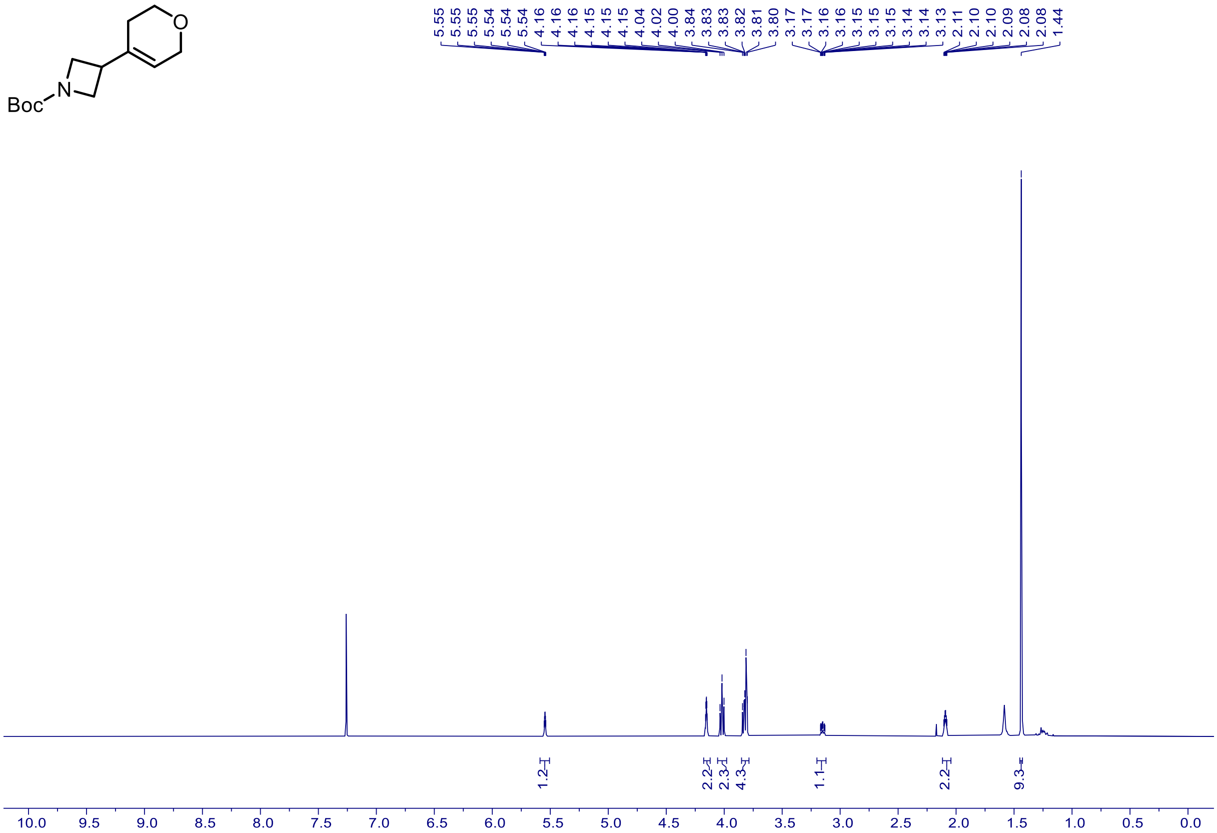

38 - ^{13}C NMR (126 MHz, $CDCl_3$)

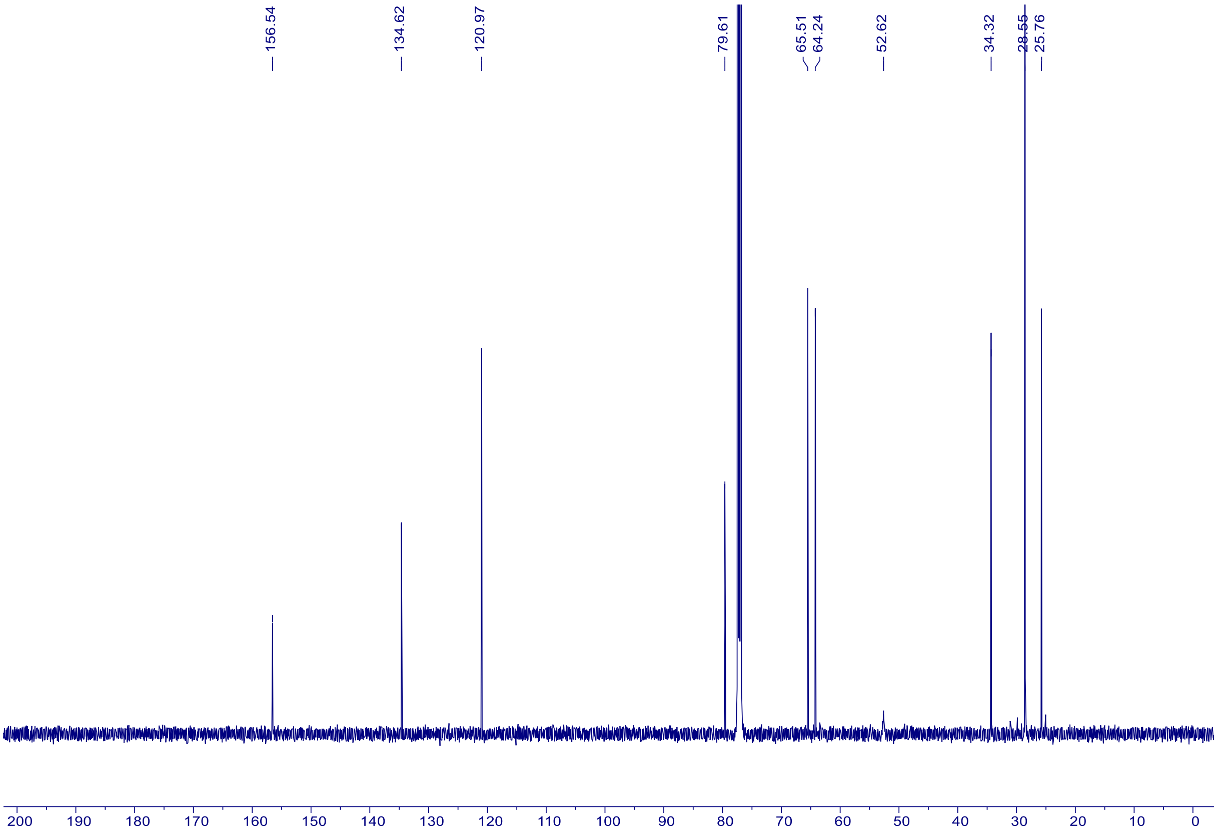

39 - ^{1}H NMR (400 MHz, $CDCl_3$)

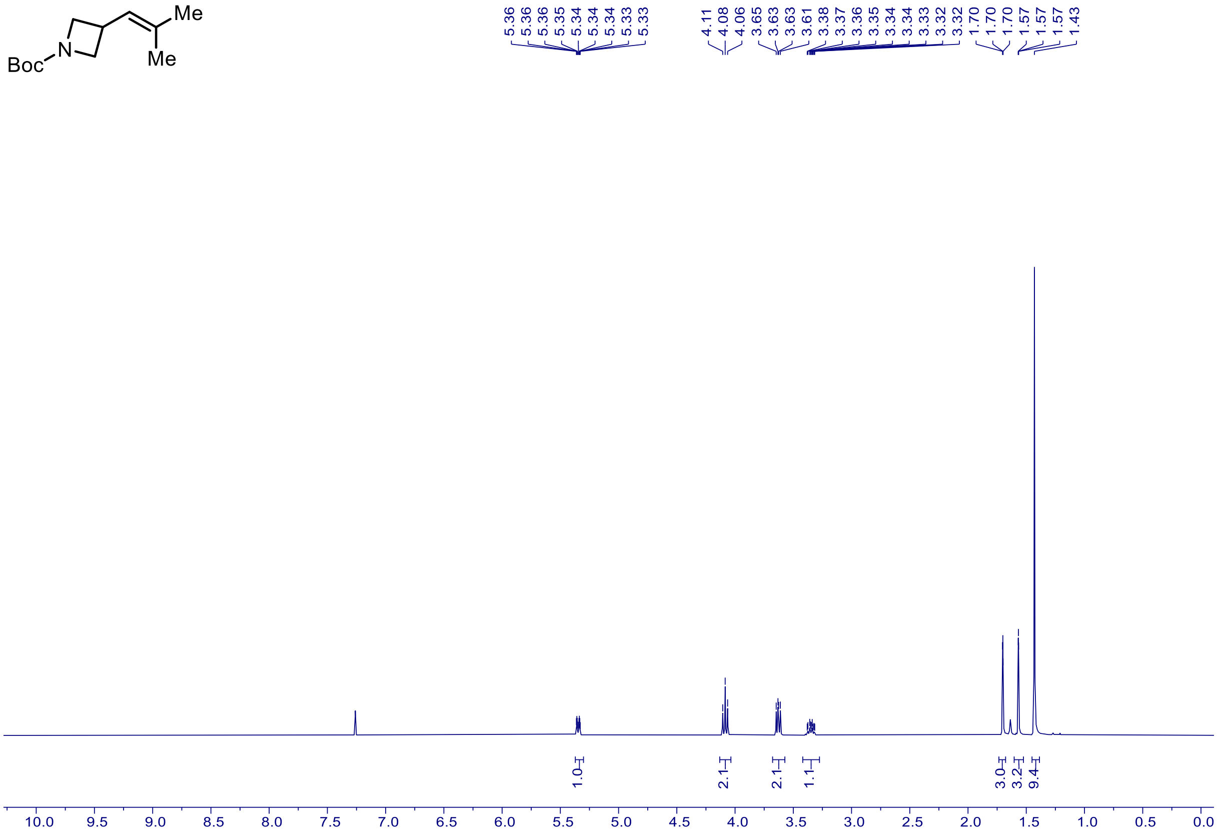

39 - ^{13}C NMR (101 MHz, $CDCl_3$)

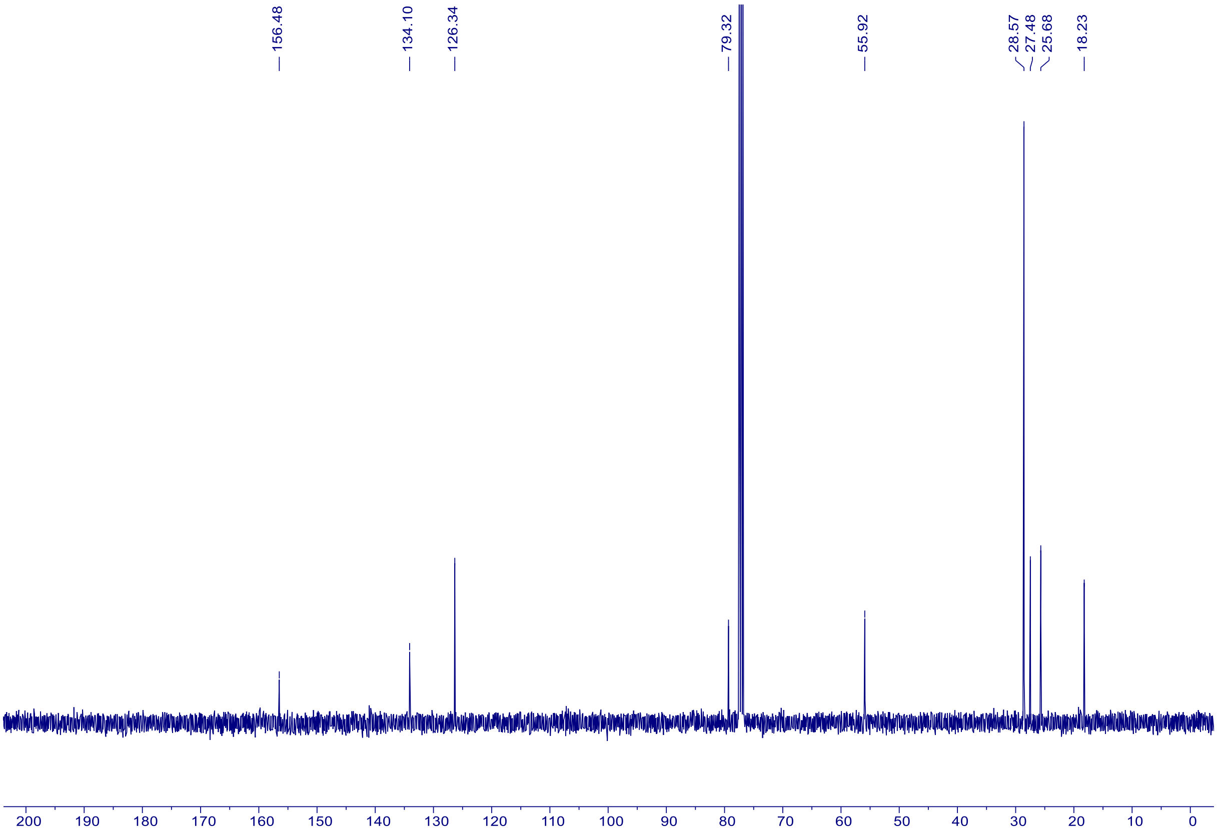

42 - ^{1}H NMR (500 MHz, $CDCl_3$)

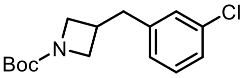

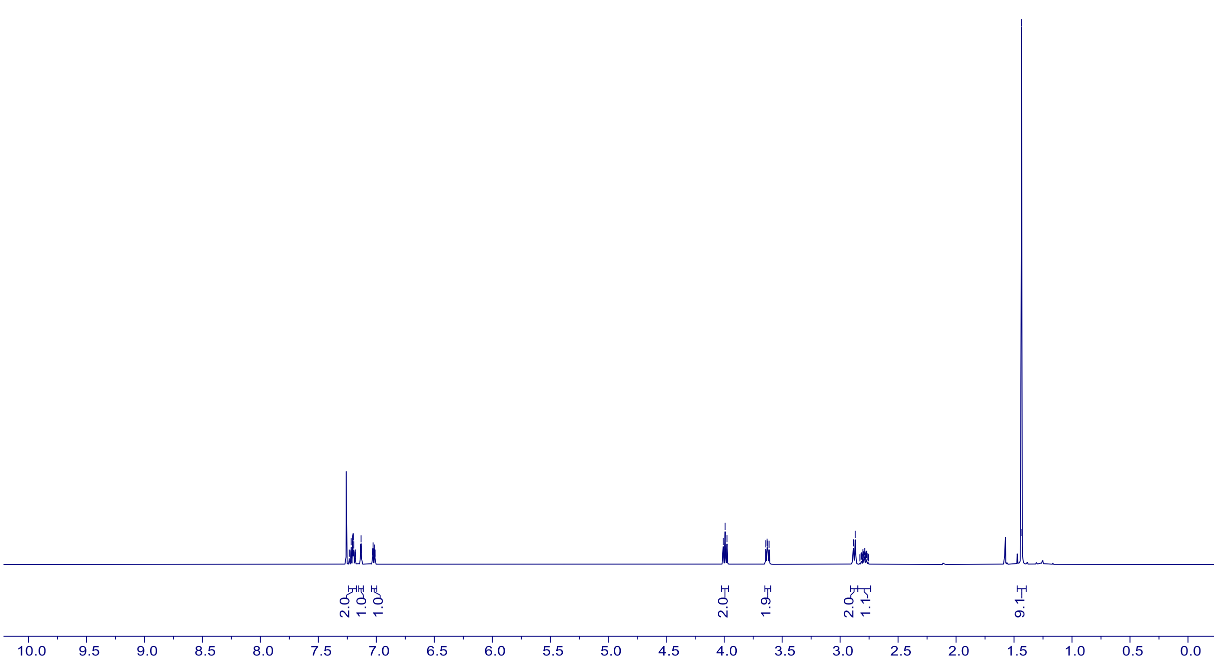

42 - ^{13}C NMR (126 MHz, $CDCl_3$)

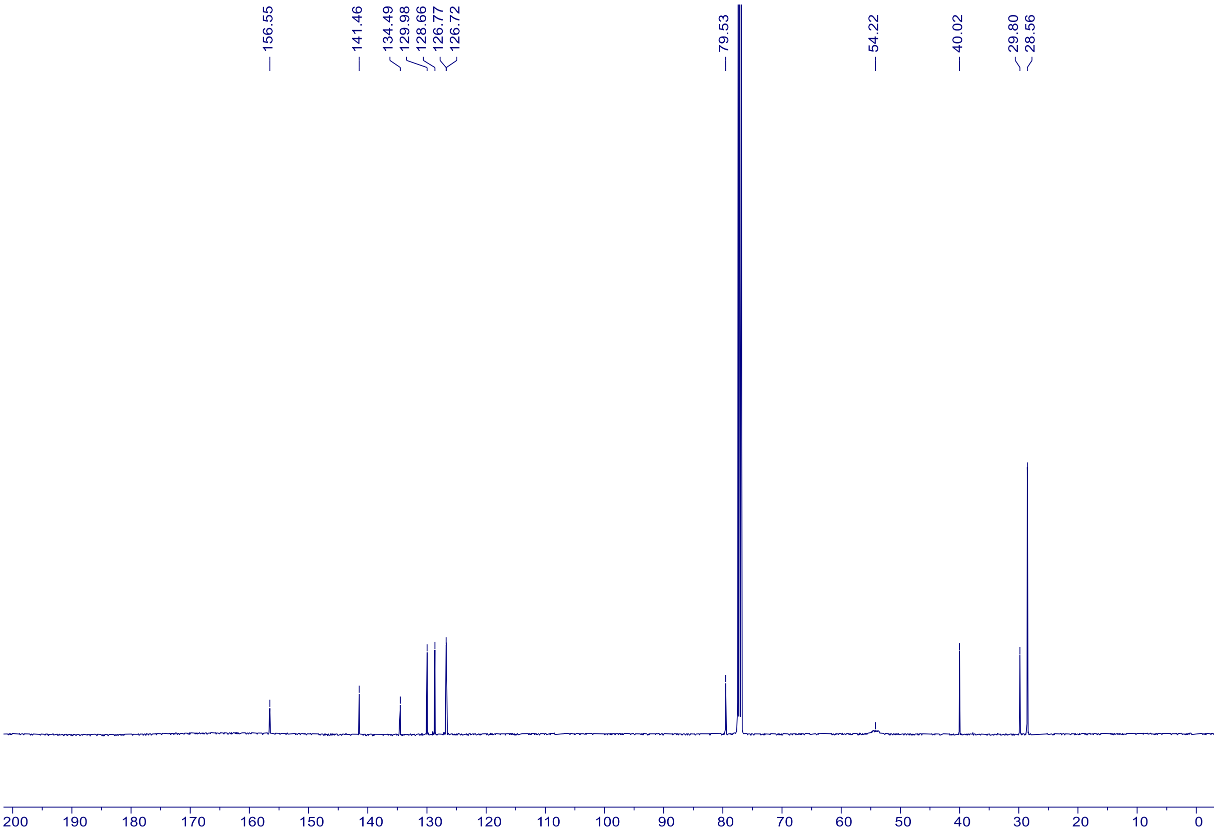

43 - ^{1}H NMR (500 MHz, $CDCl_3$)

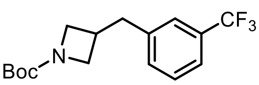

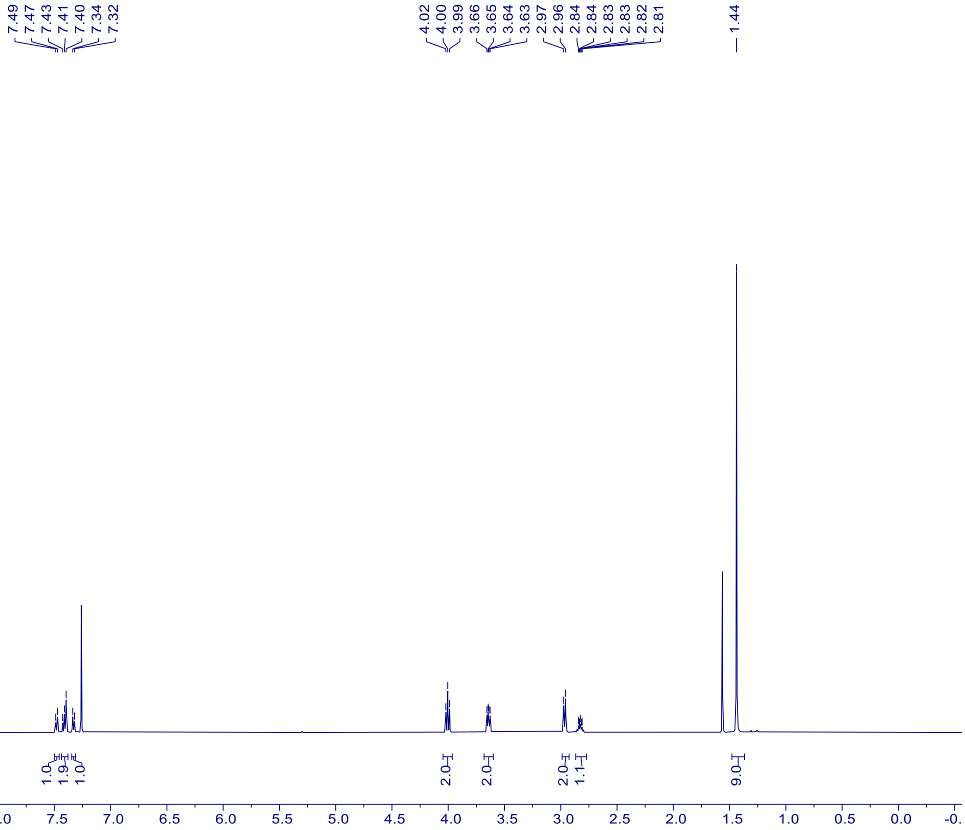

43 - ^{13}C NMR (126 MHz, $CDCl_3$)

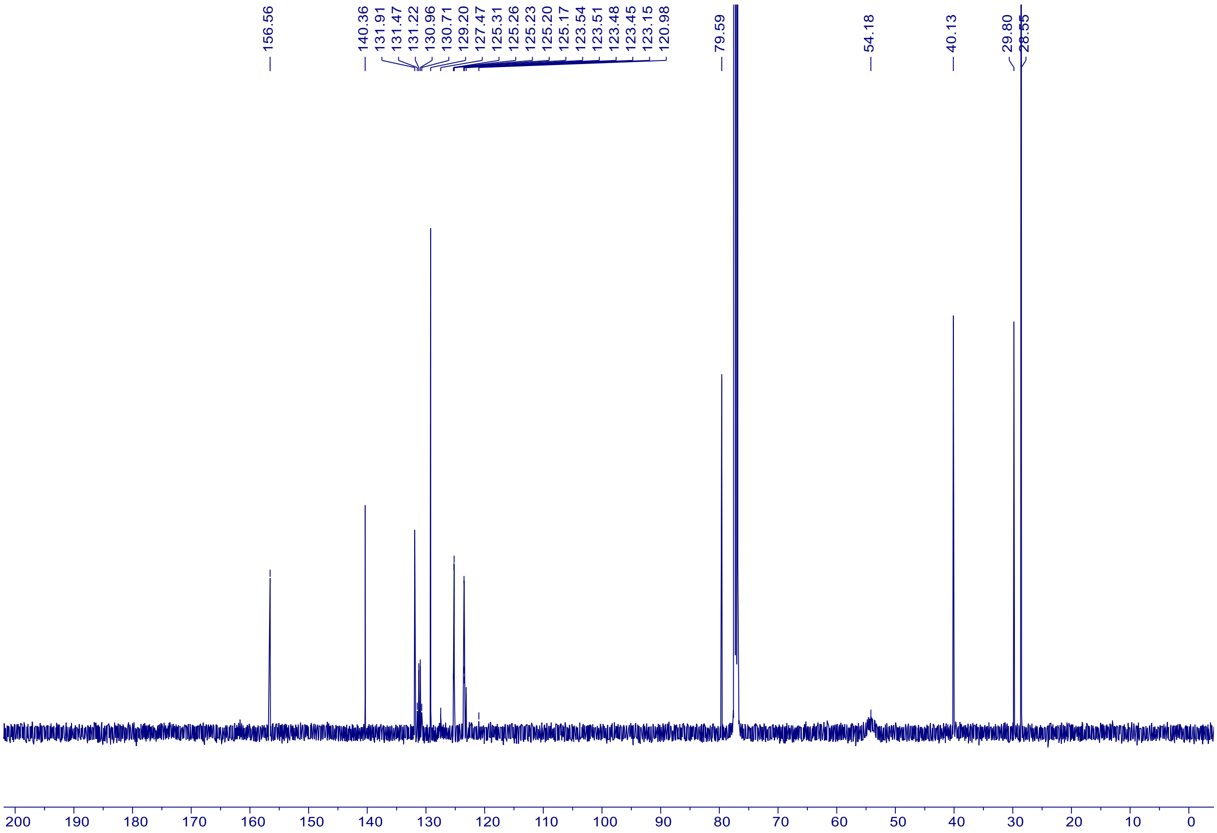

43 - ^{19}F NMR (376 MHz, $CDCl_3$)

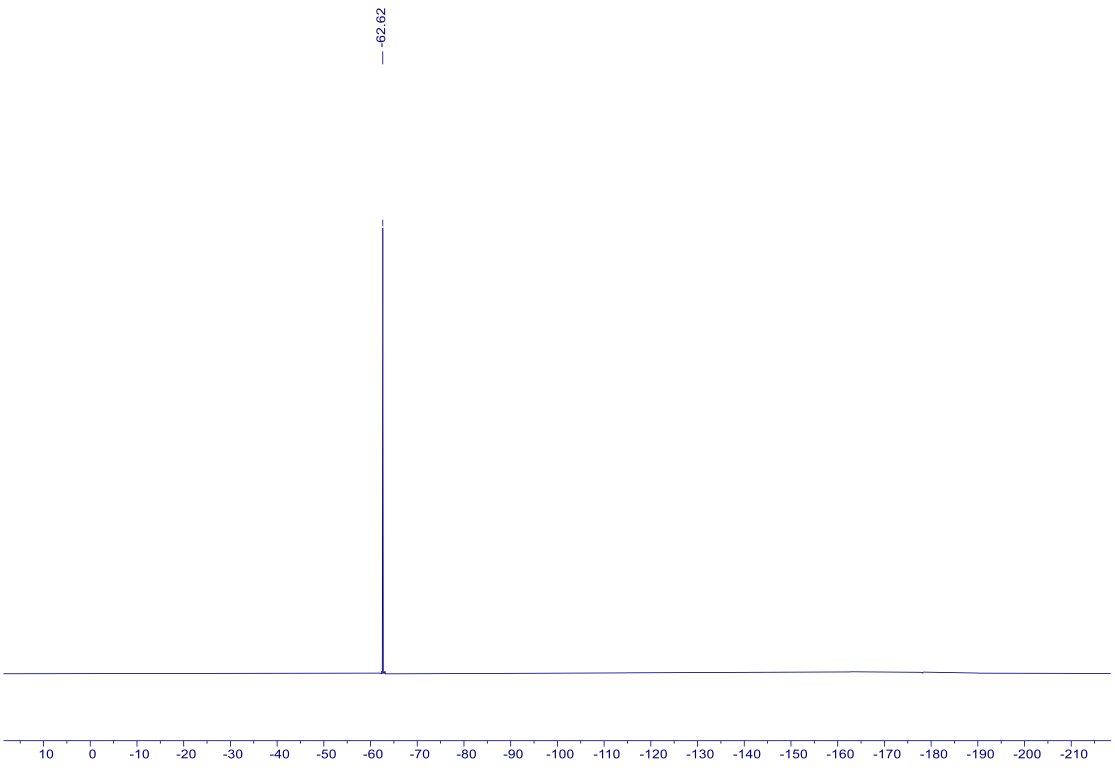

44 - ^{1}H NMR (400 MHz, $CDCl_3$)

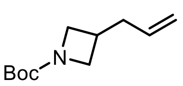

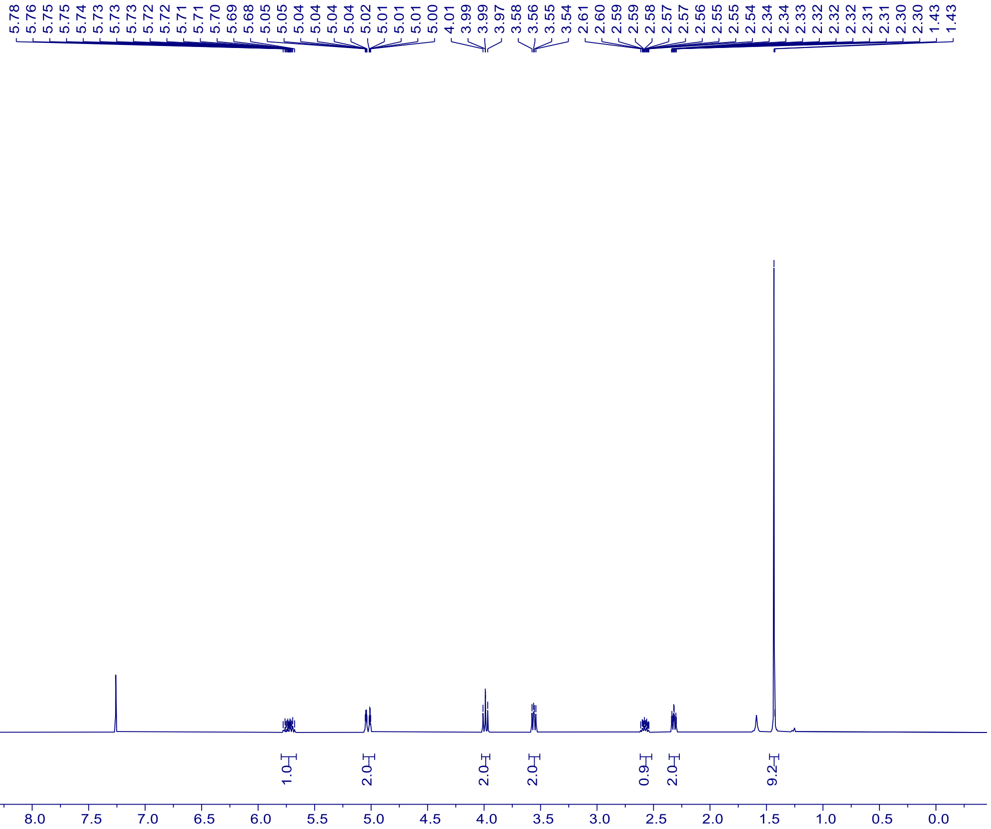

44 - ^{13}C NMR (126 MHz, $CDCl_3$)

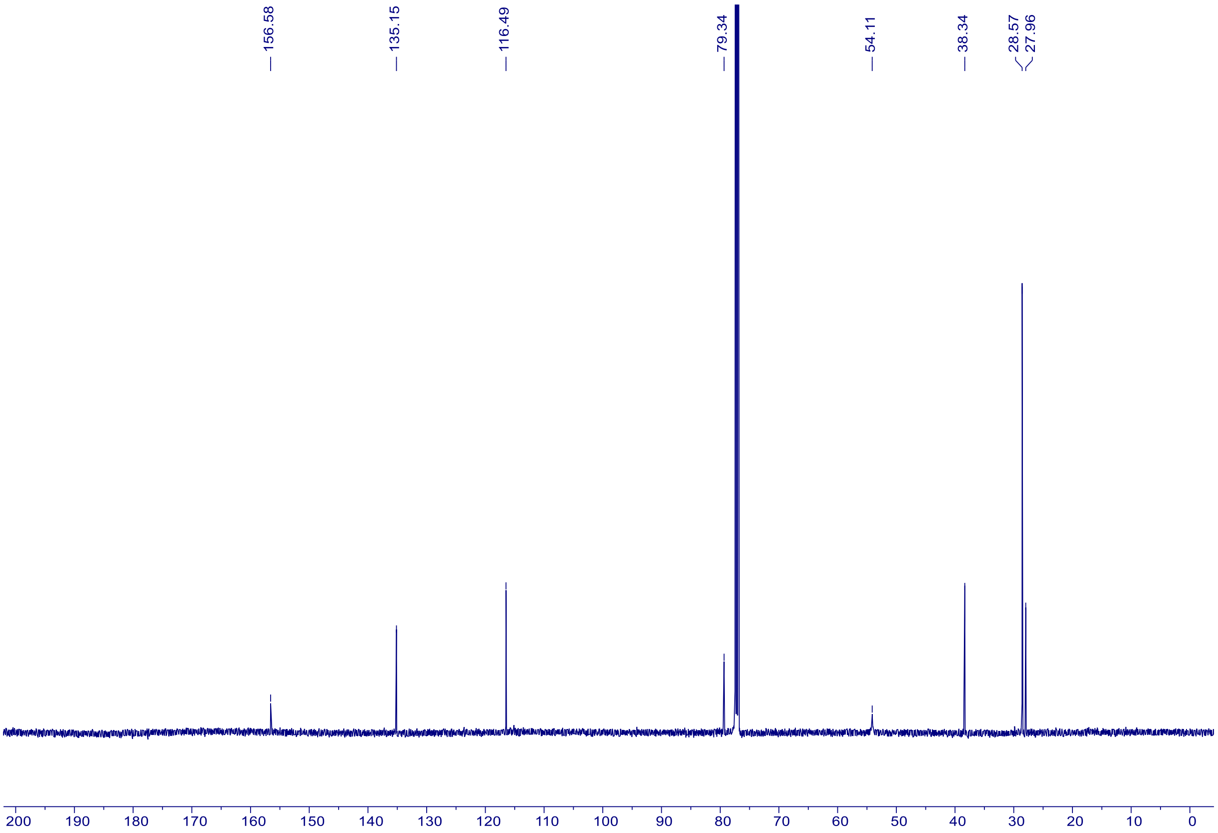

49 - ^{1}H NMR (500 MHz, $CDCl_3$)

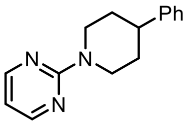

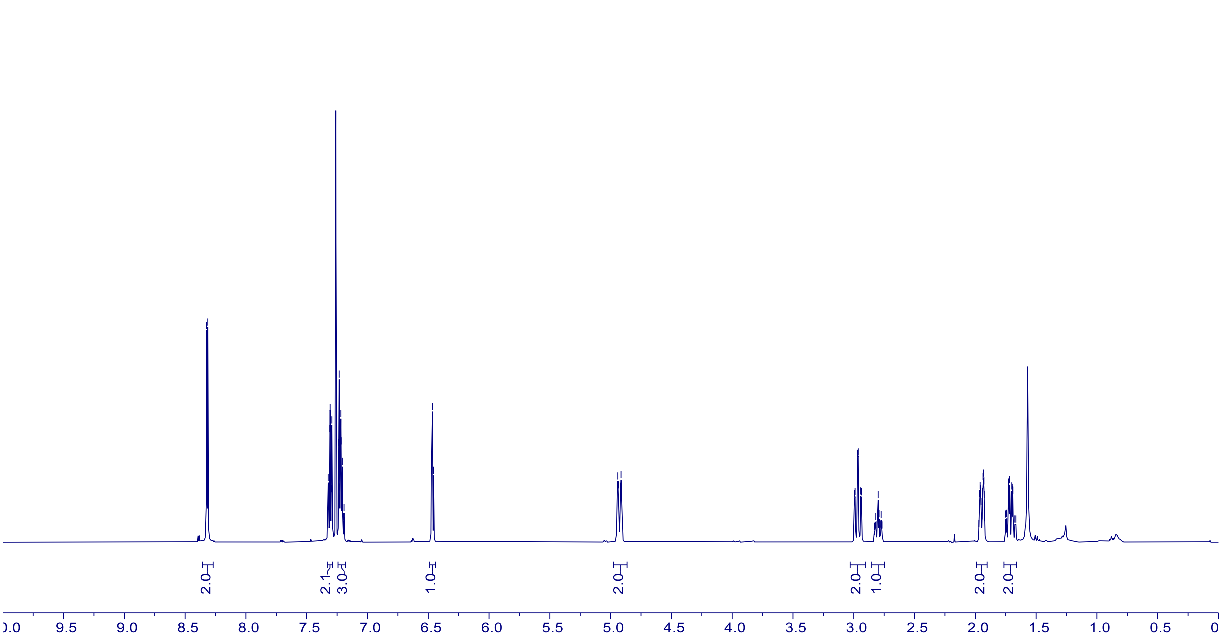

49 - ^{13}C NMR (126 MHz, $CDCl_3$)

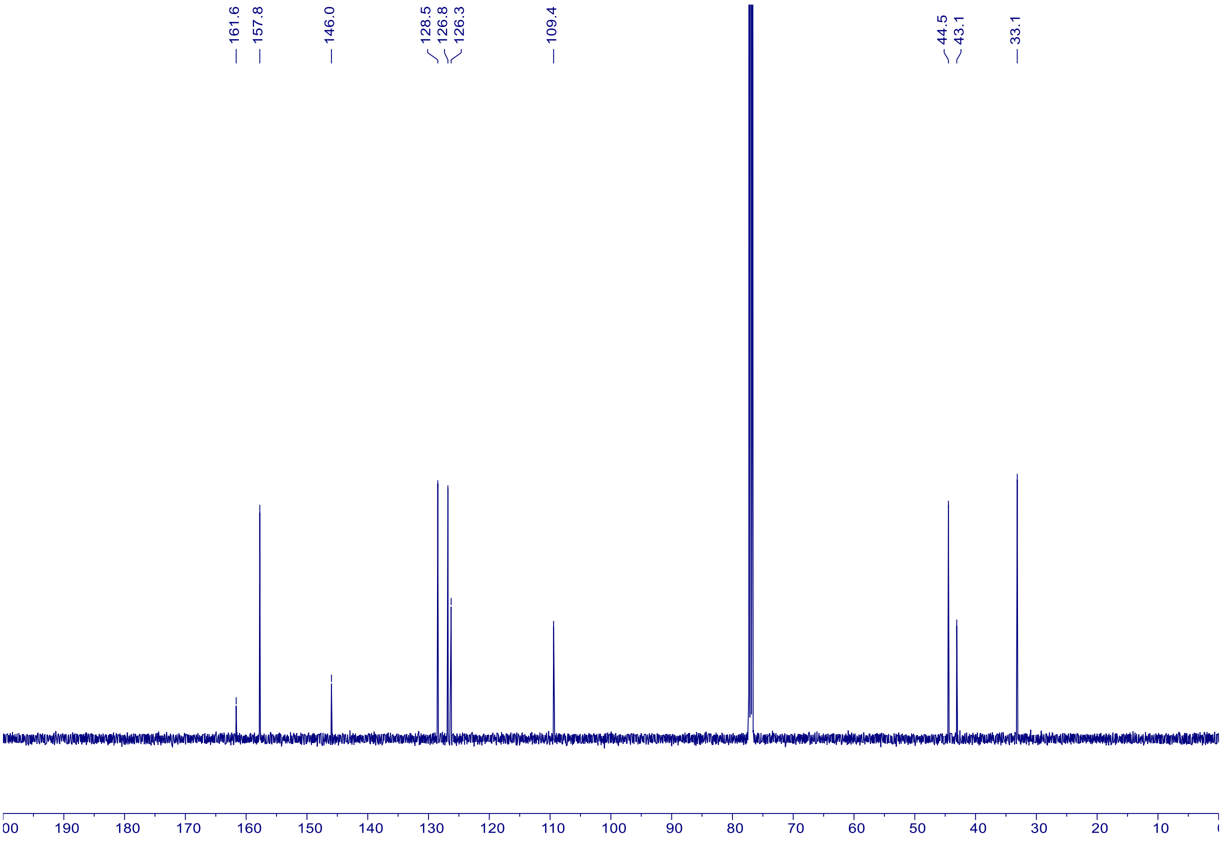

50 - ^{1}H NMR (400 MHz, $CDCl_3$)

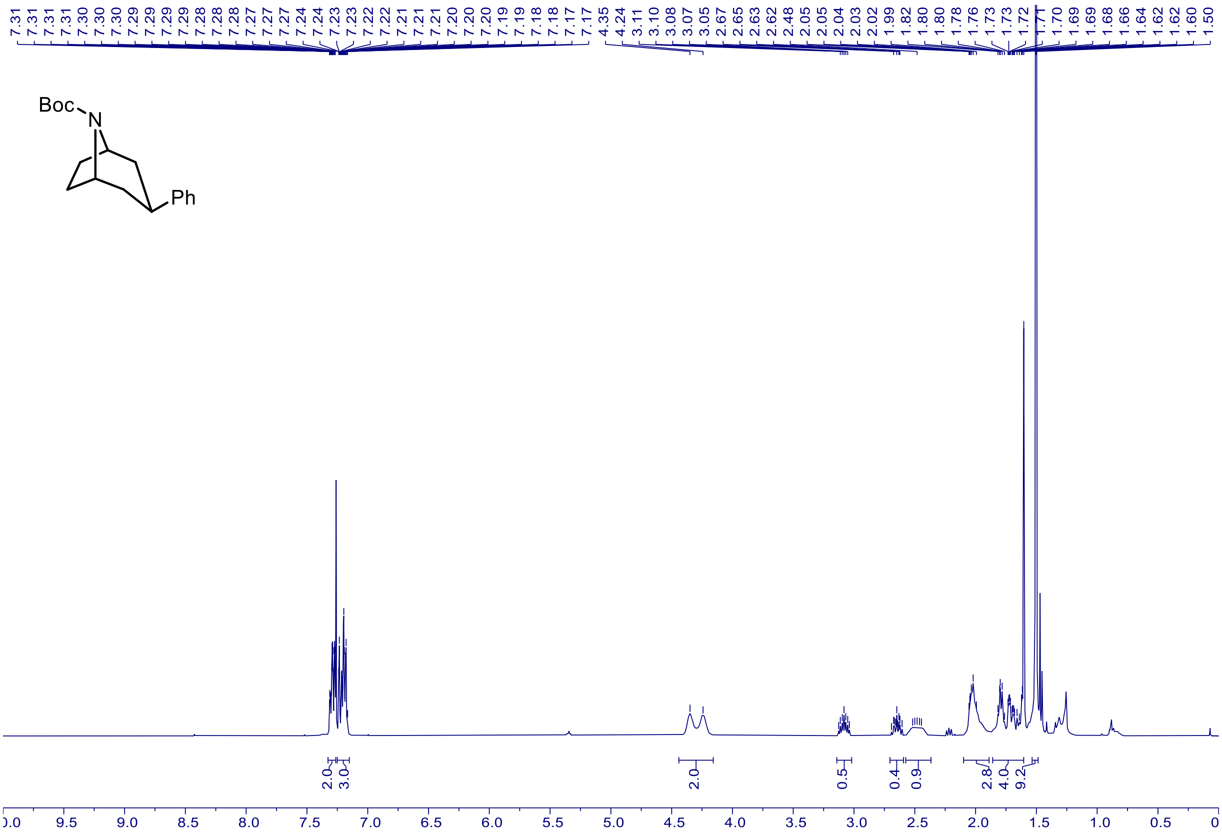

50 - ^{13}C NMR (101 MHz, $CDCl_3$)

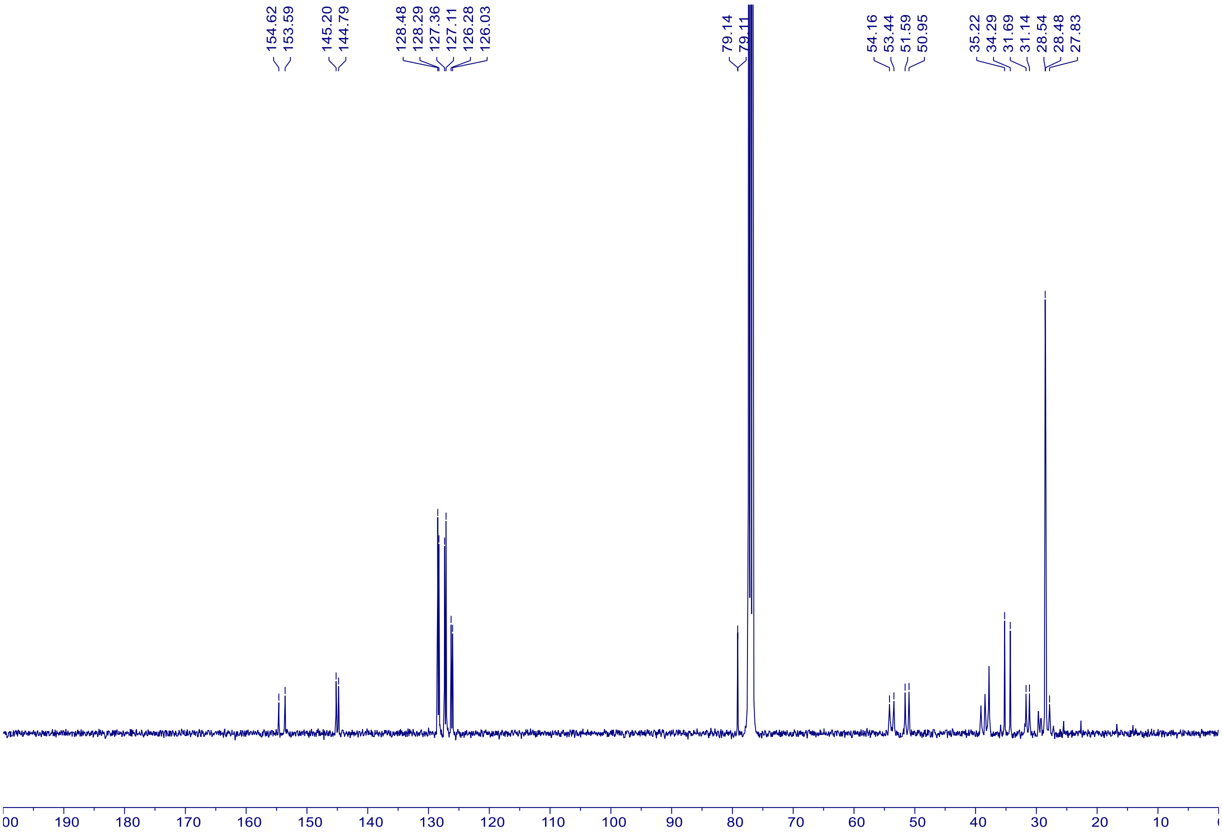

52 - ^{1}H NMR (500 MHz, $CDCl_3$)

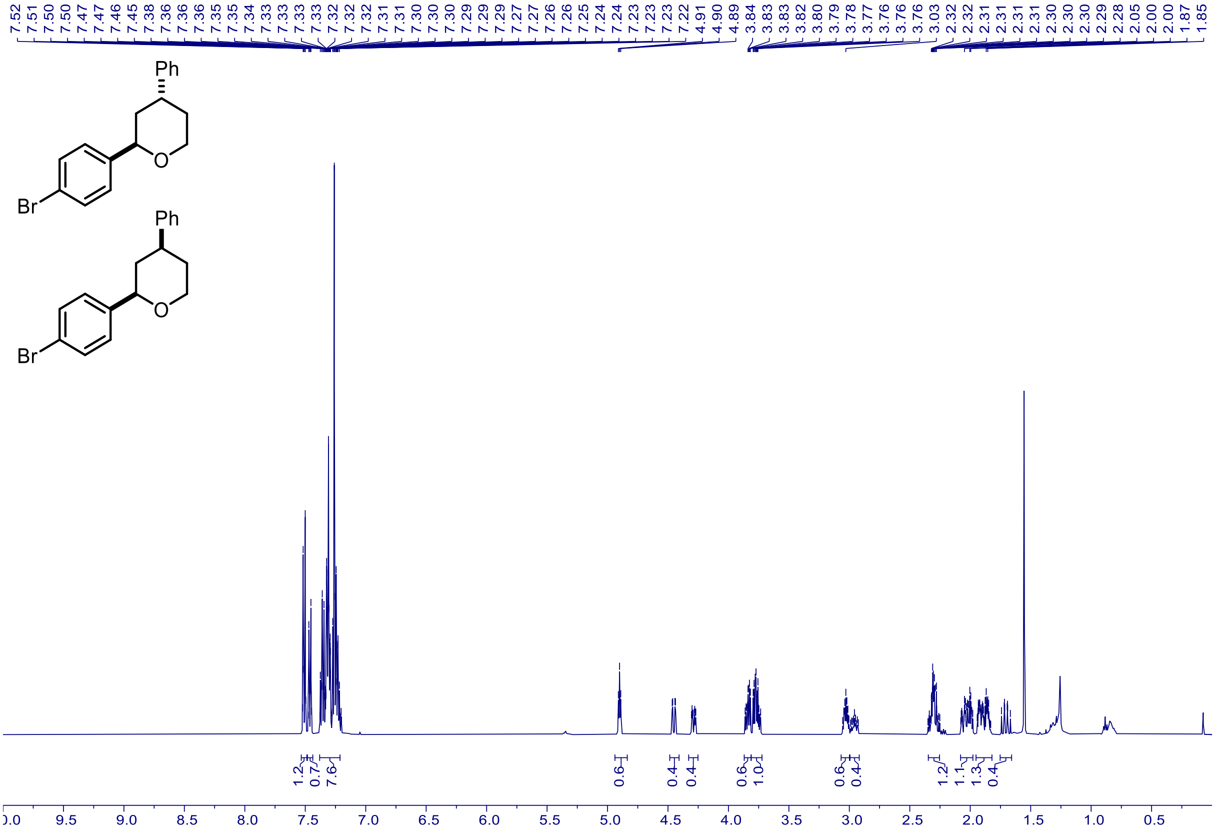

52 - ^{13}C NMR (126 MHz, $CDCl_3$)

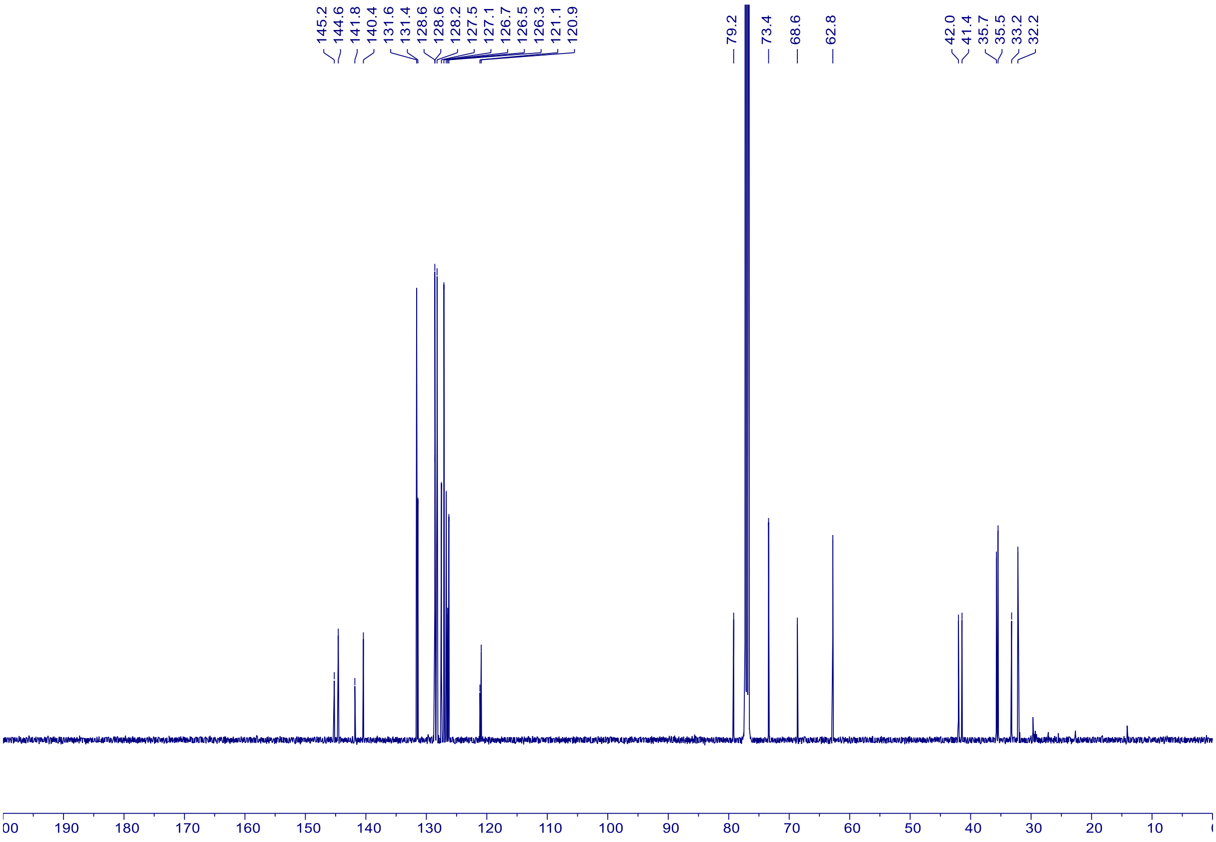

53 - ^{1}H NMR (500 MHz, $CDCl_3$)

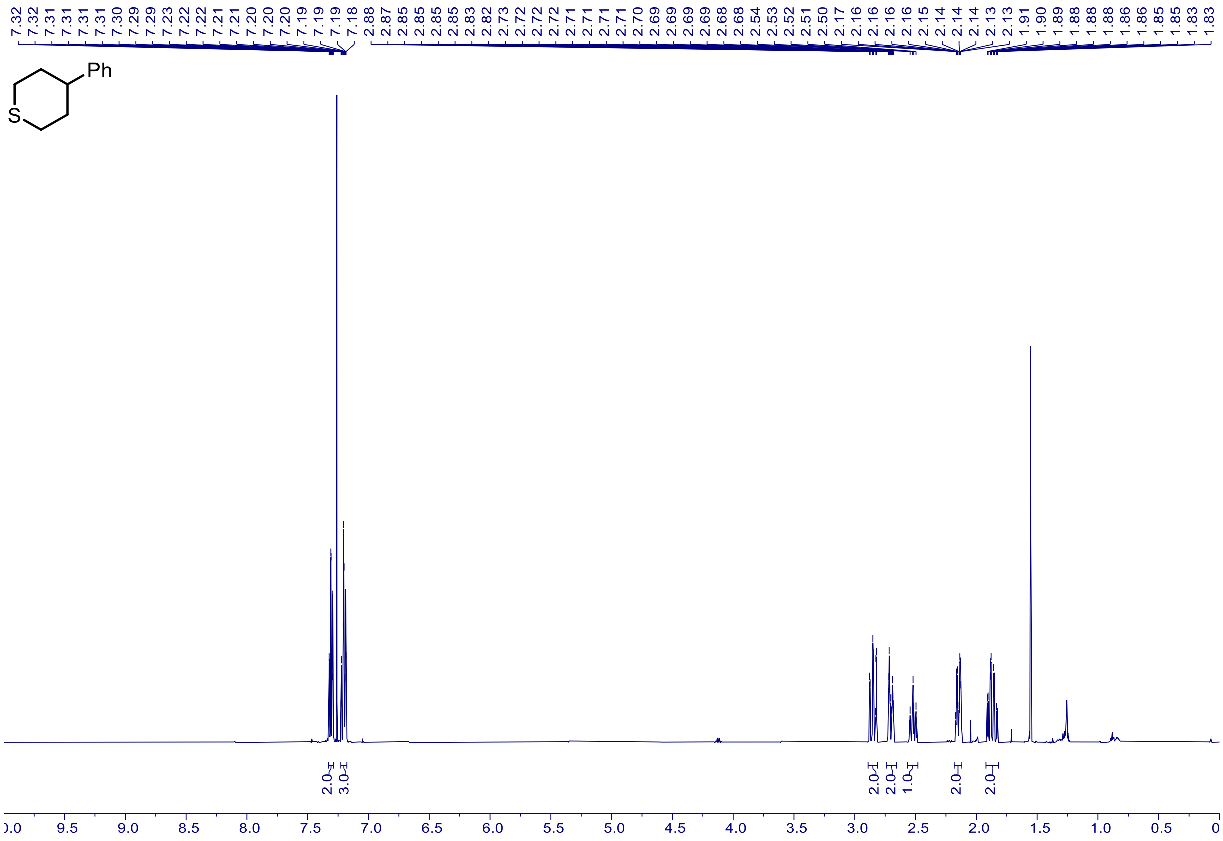

53 - ^{13}C NMR (126 MHz, $CDCl_3$)

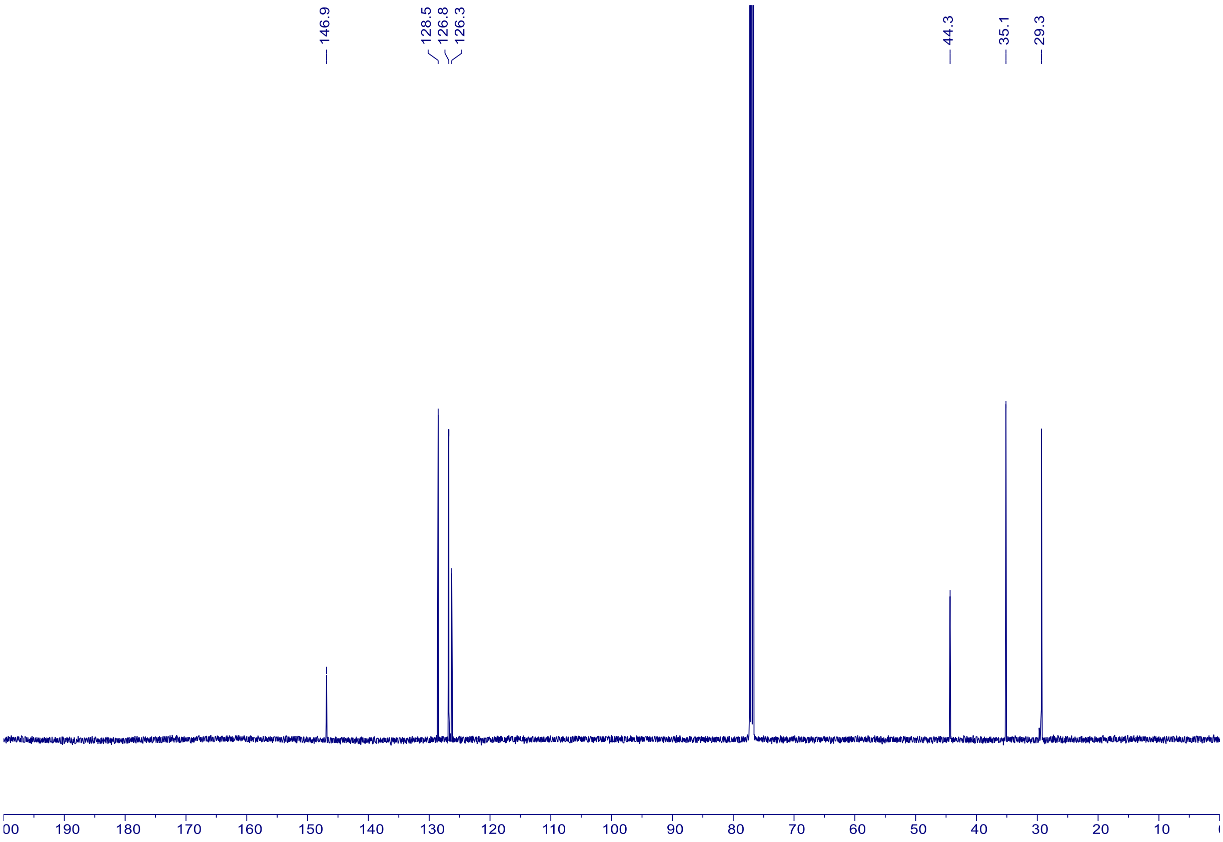

55 - ^{1}H NMR (400 MHz, $CDCl_3$)

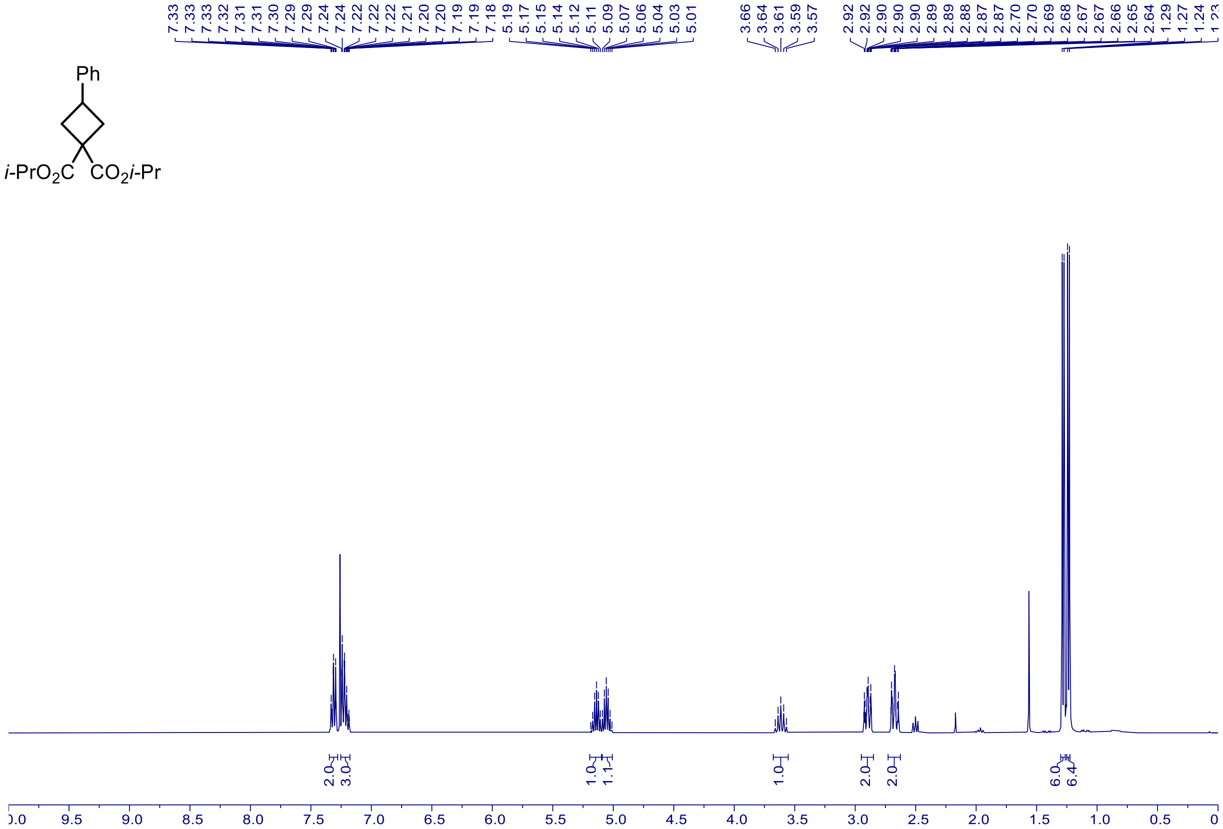

55 - ^{13}C NMR (101 MHz, $CDCl_3$)

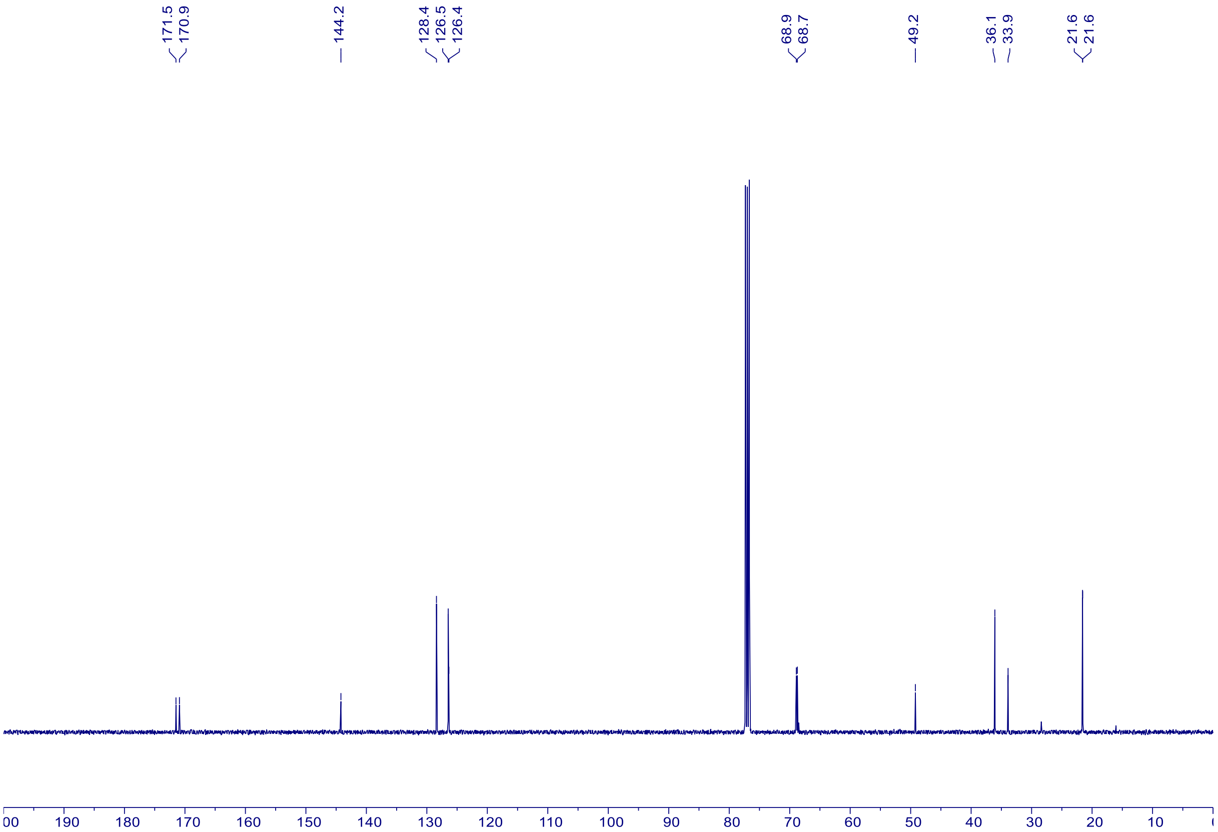

63 - ^{1}H NMR (500 MHz, $CDCl_3$)

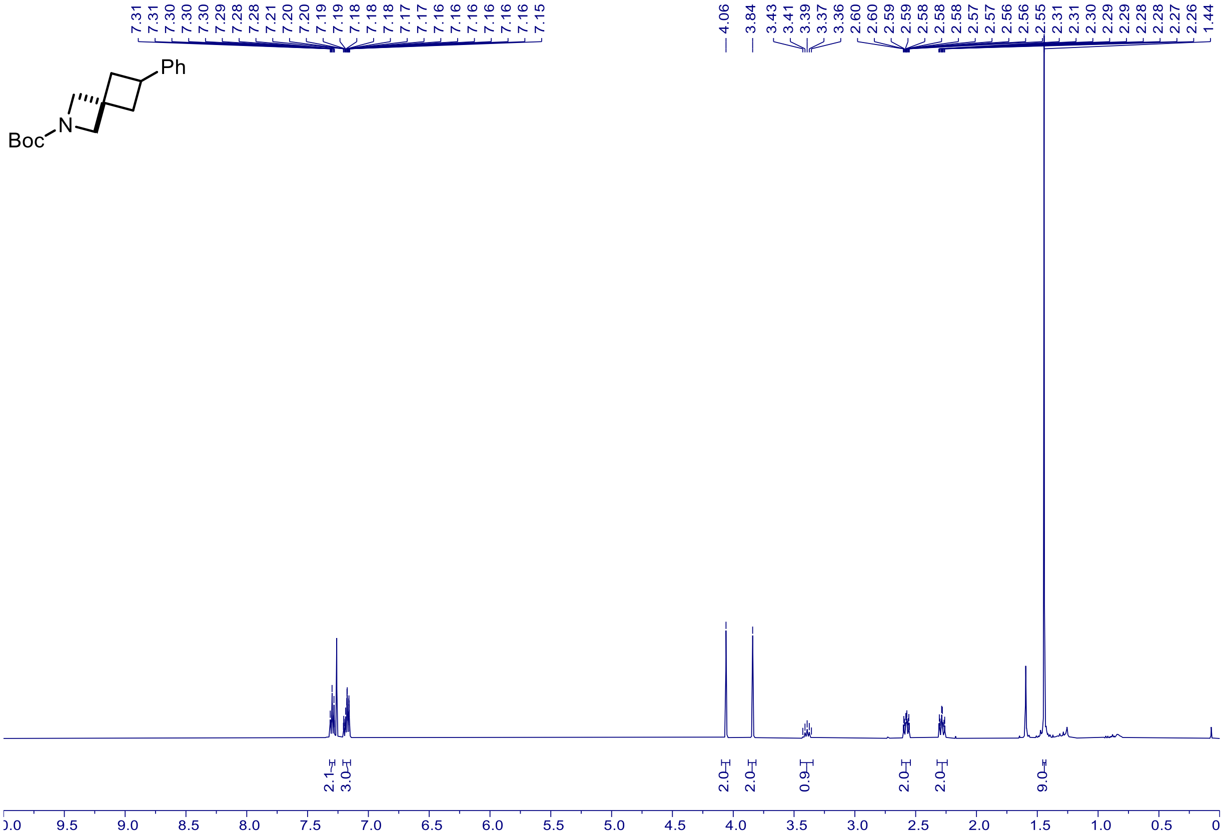

63 - ^{13}C NMR (126 MHz, $CDCl_3$)

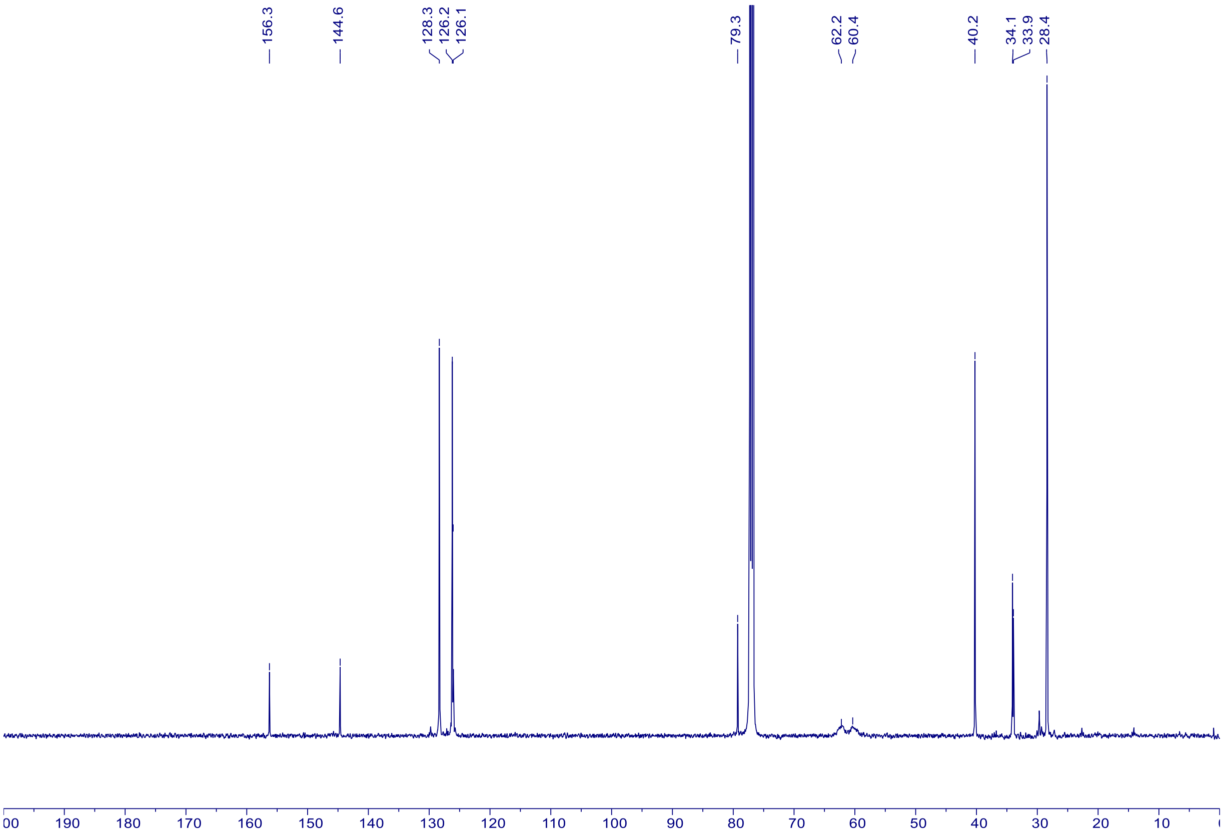

64 - ^{1}H NMR (400 MHz, $CDCl_3$)

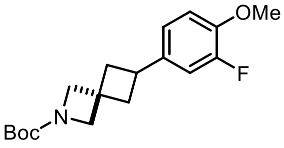

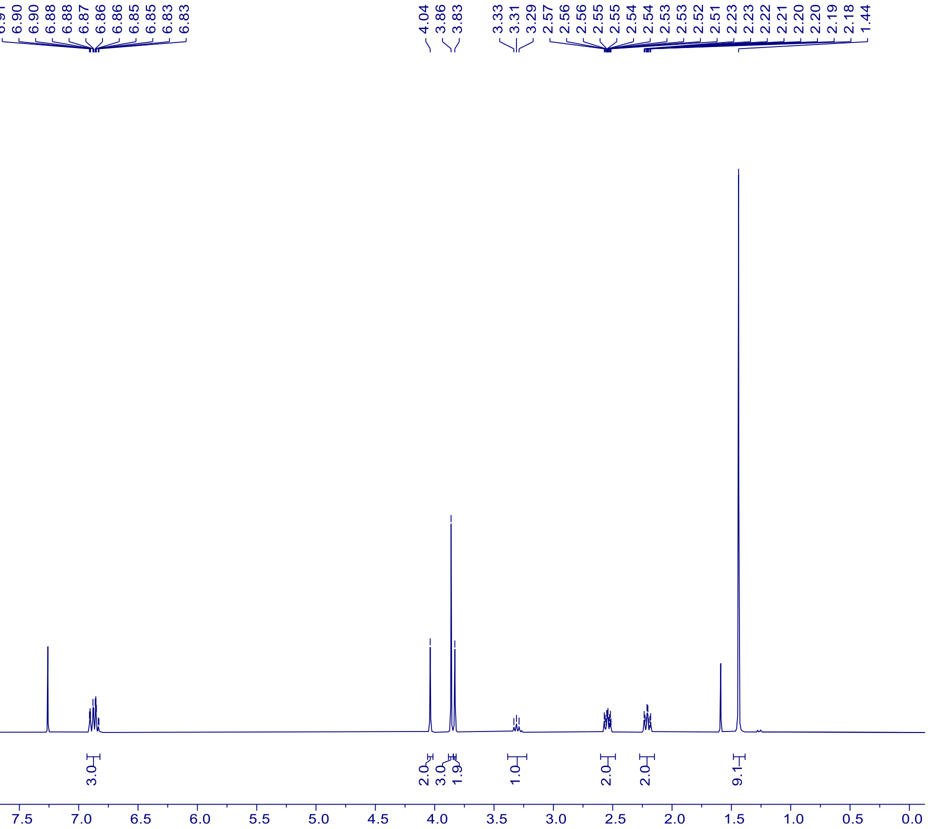

64 - ^{13}C NMR (101 MHz, $CDCl_3$)

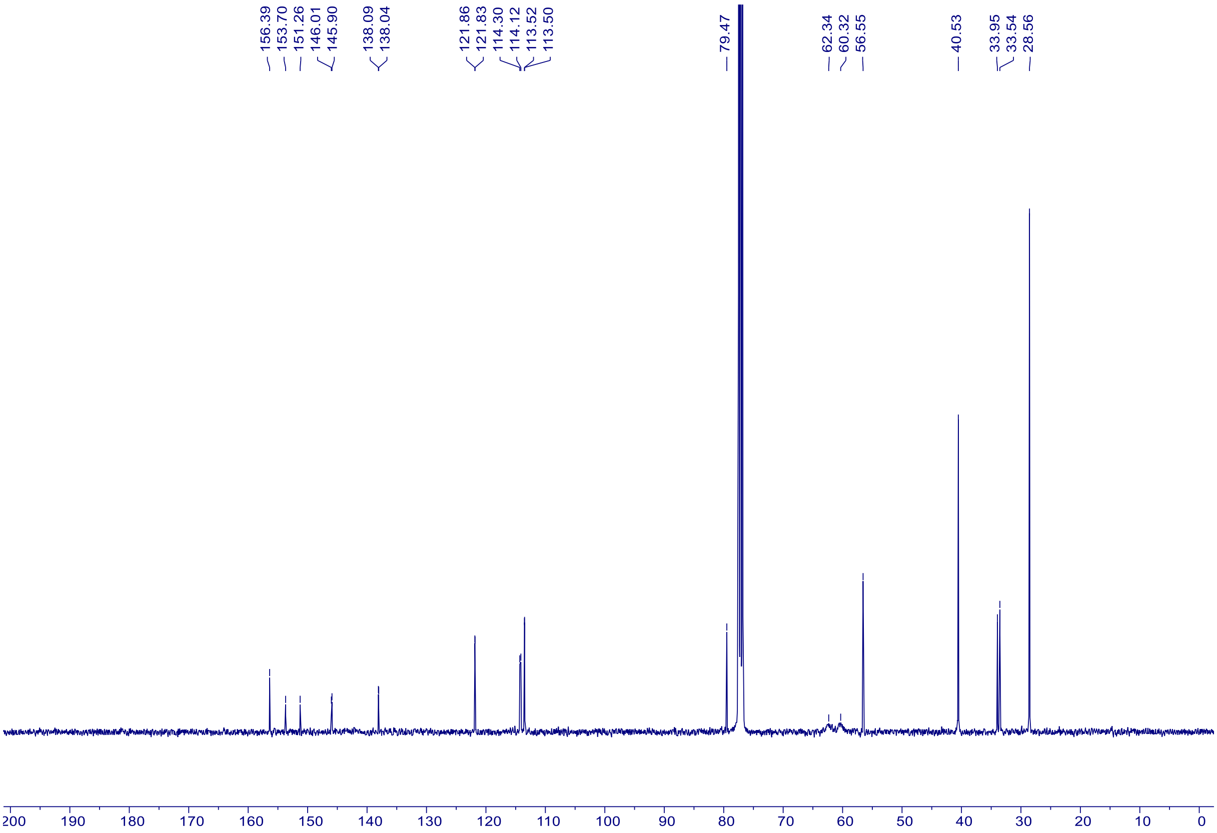

64 - ^{19}F NMR (376 MHz, $CDCl_3$)

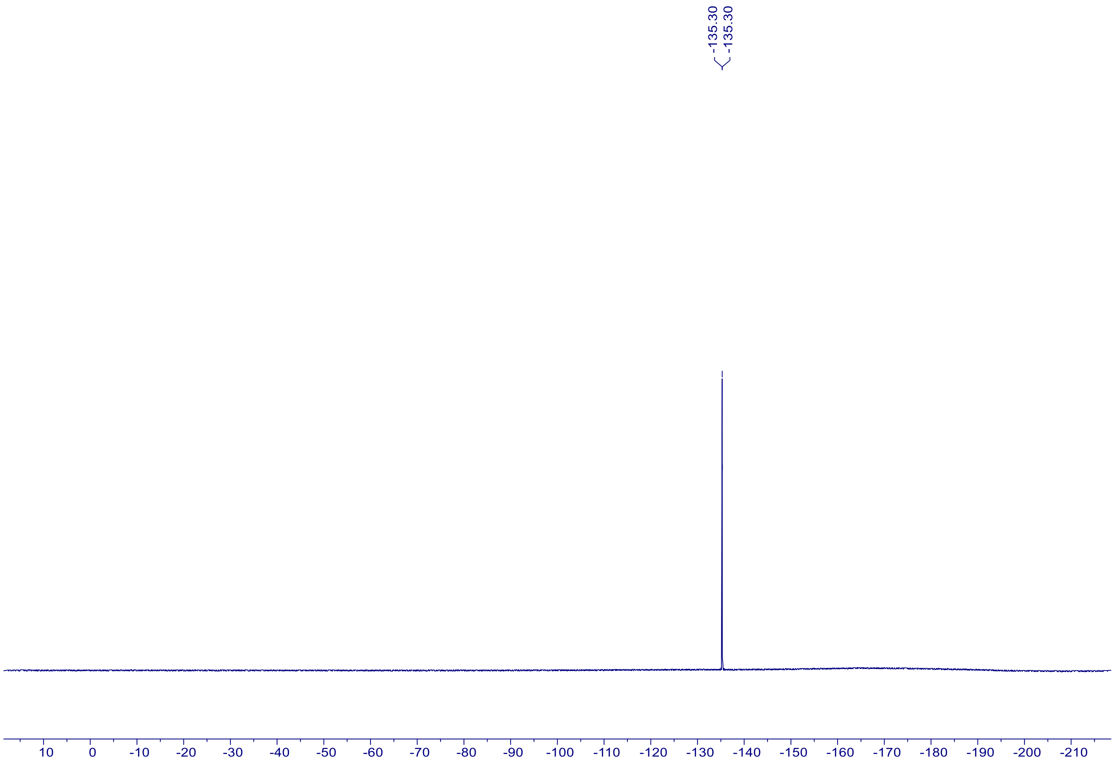

65 - ^{1}H NMR (500 MHz, $CDCl_3$)

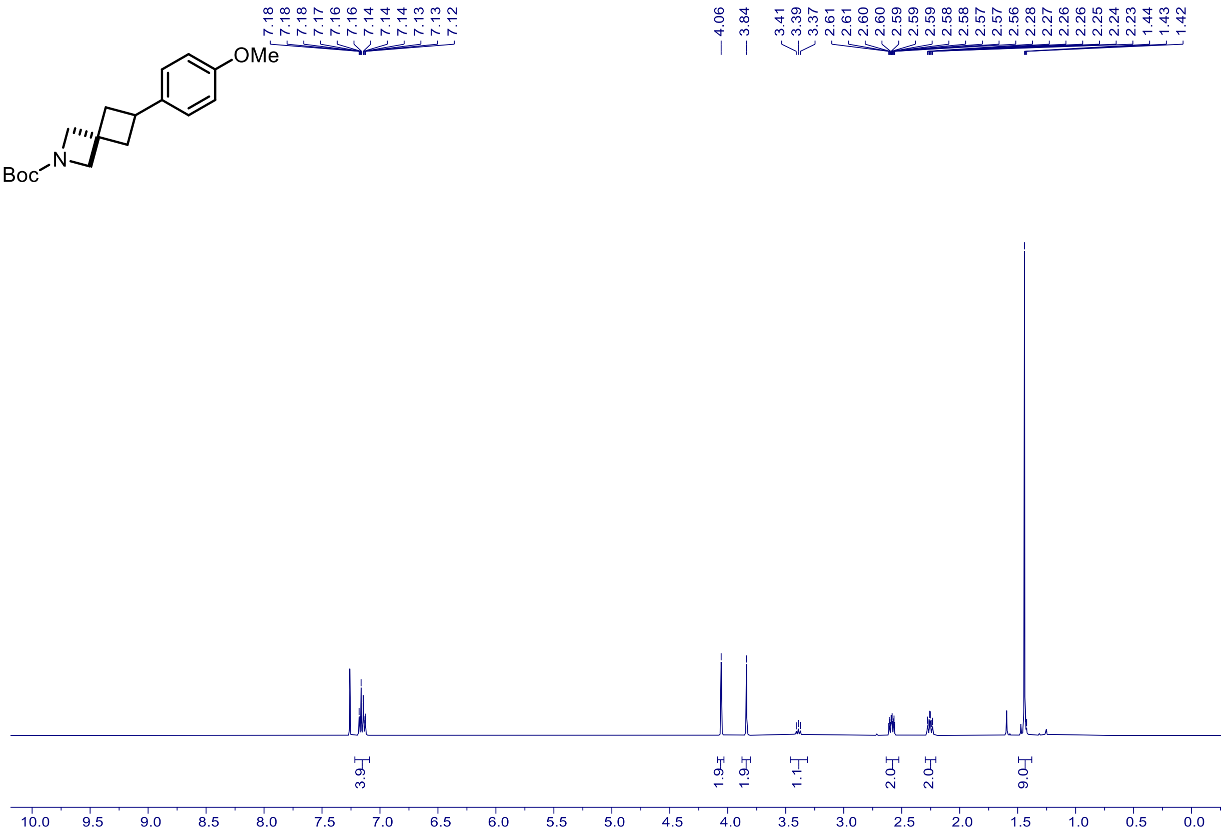

65 - ^{13}C NMR (126 MHz, $CDCl_3$)

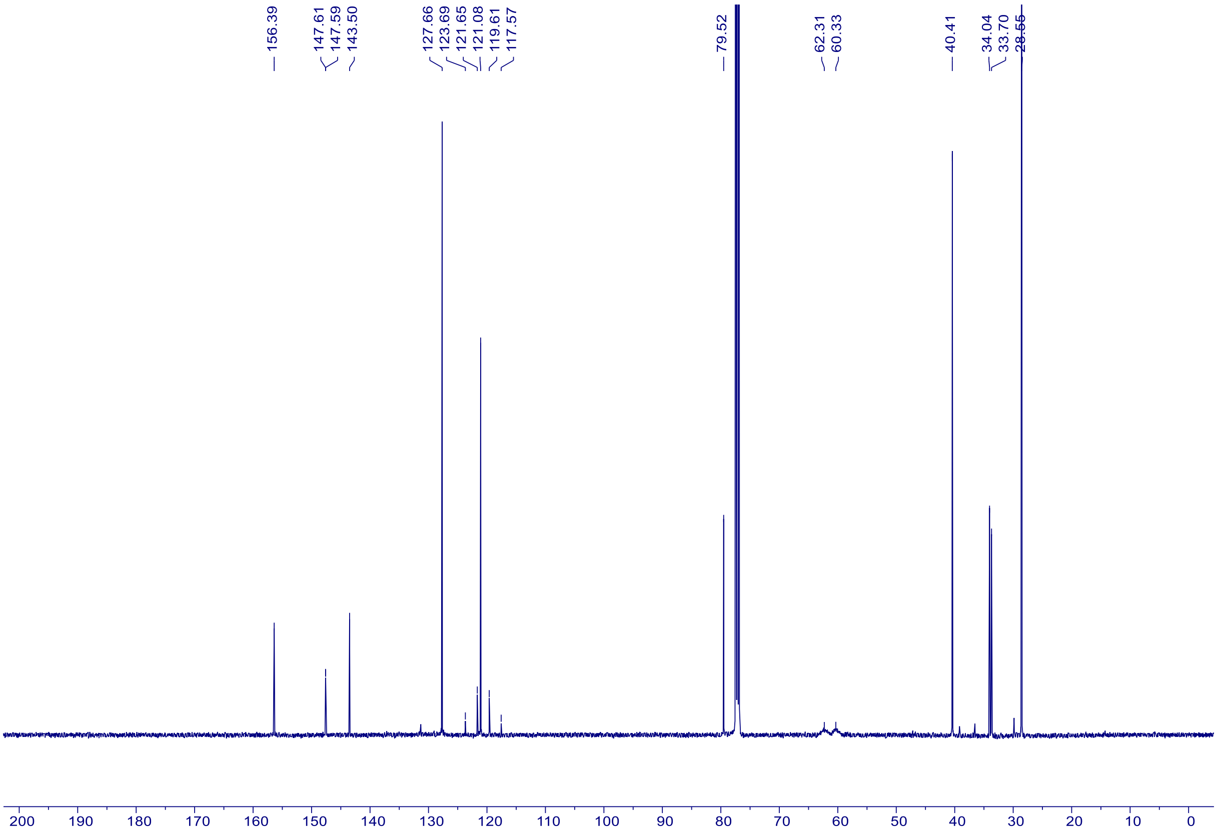

65 - ^{19}F NMR (376 MHz, $CDCl_3$)

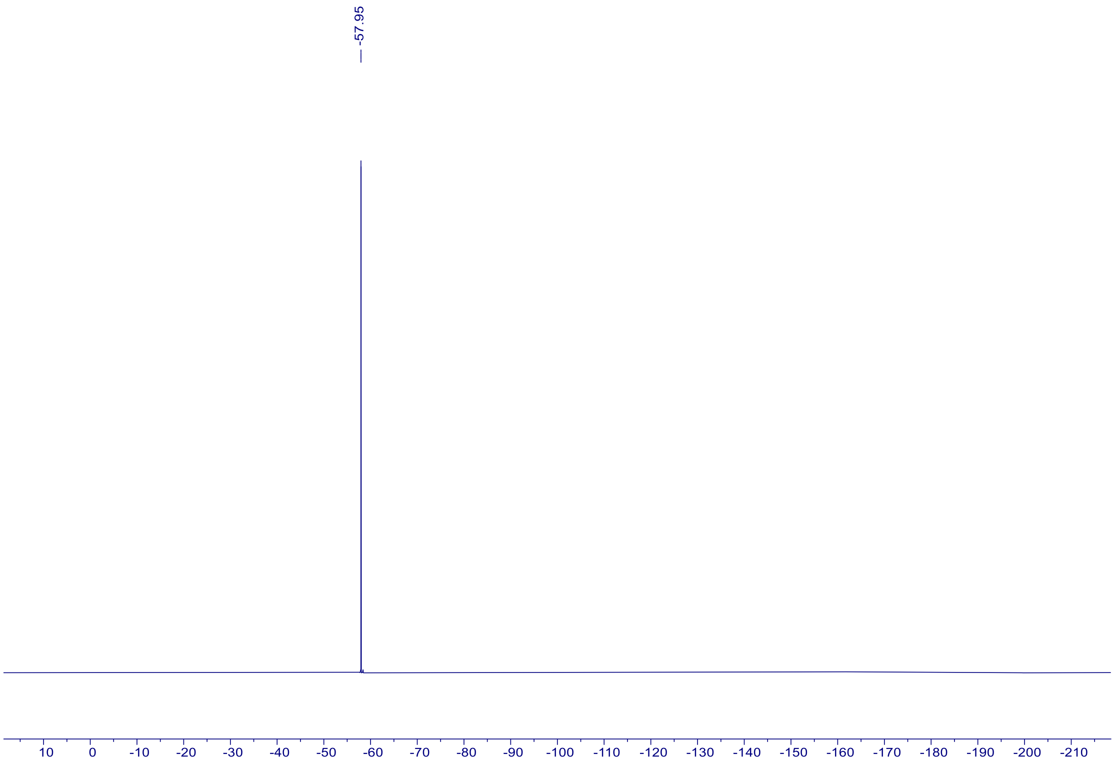

68 - ^{1}H NMR (400 MHz, $CDCl_3$)

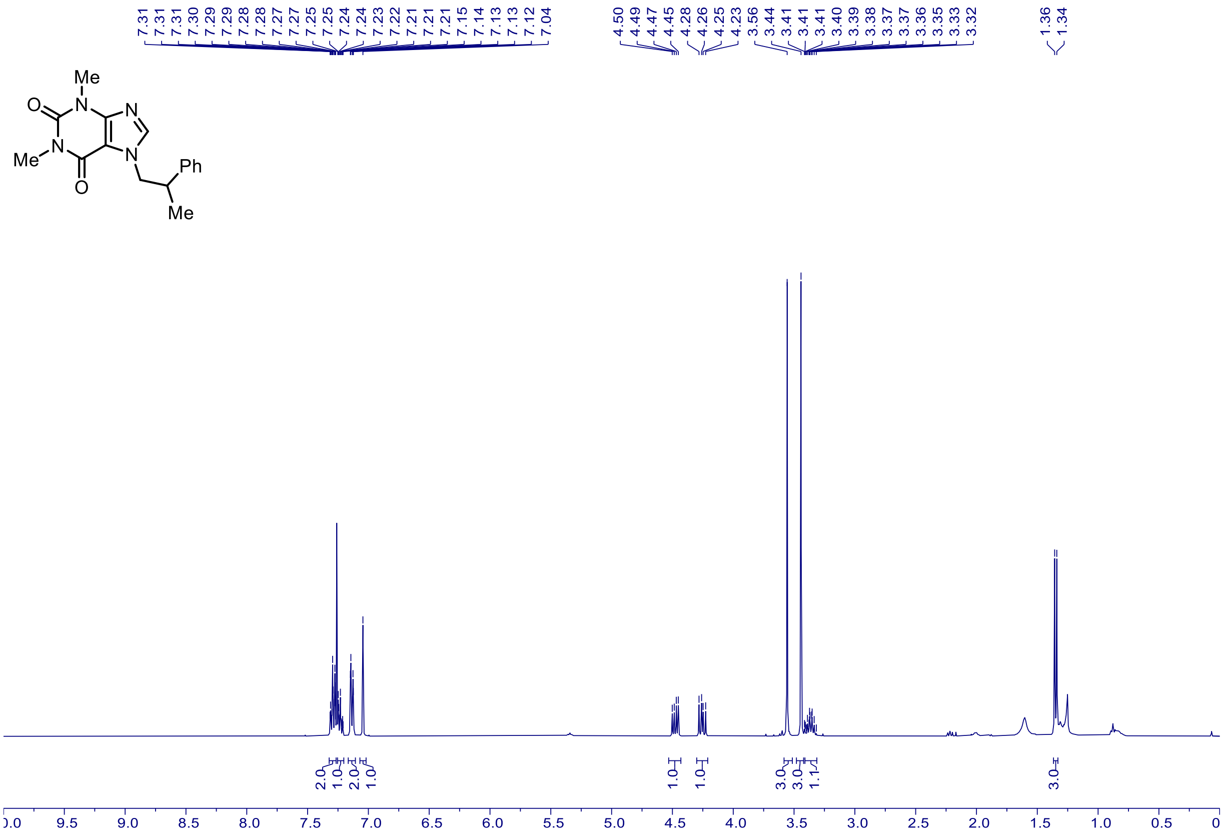

68 - ^{13}C NMR (101 MHz, $CDCl_3$)

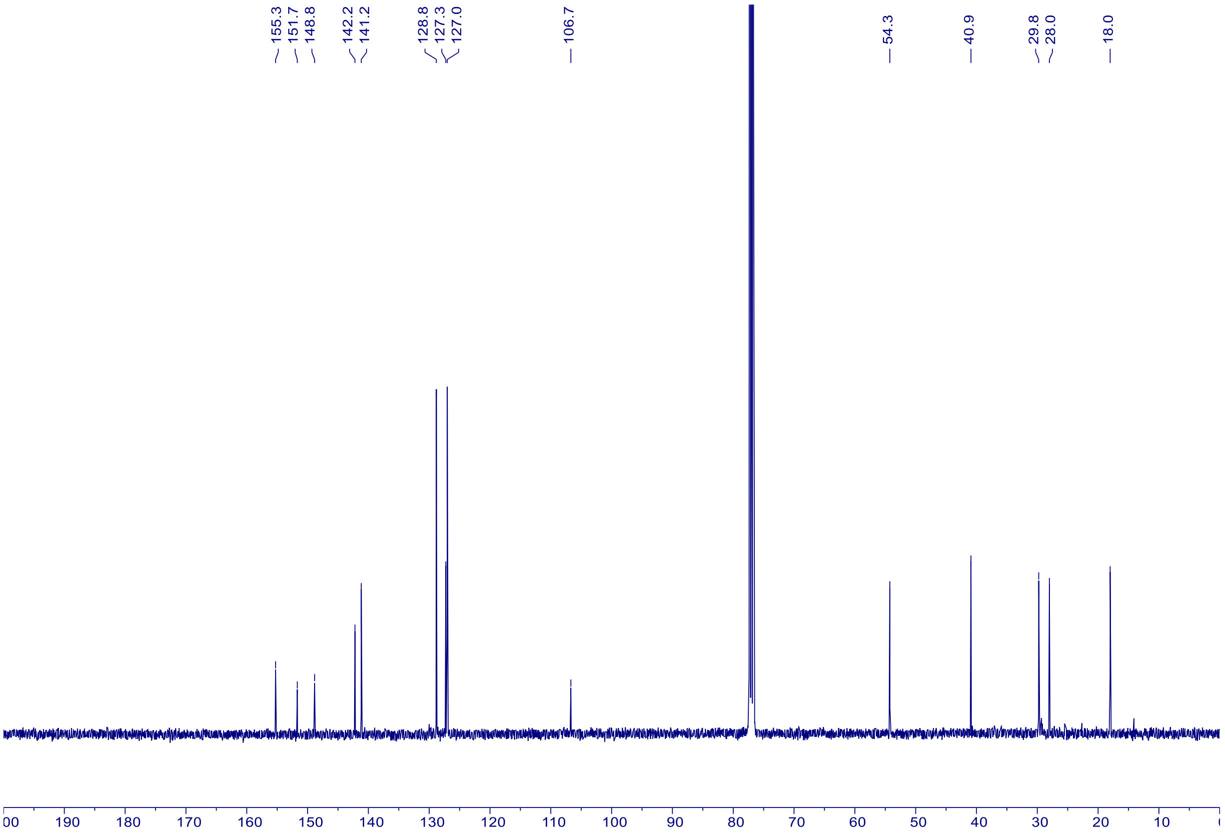

69 - ^{1}H NMR (400 MHz, $CDCl_3$)

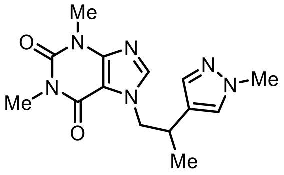

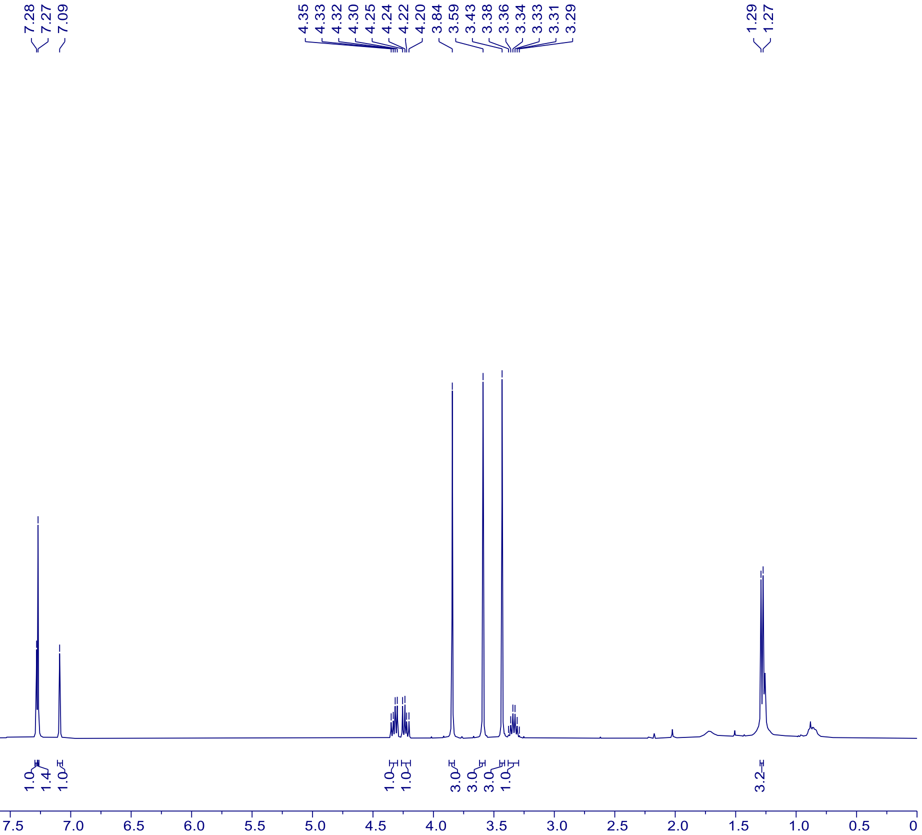

69 - ^{13}C NMR (101 MHz, $CDCl_3$)

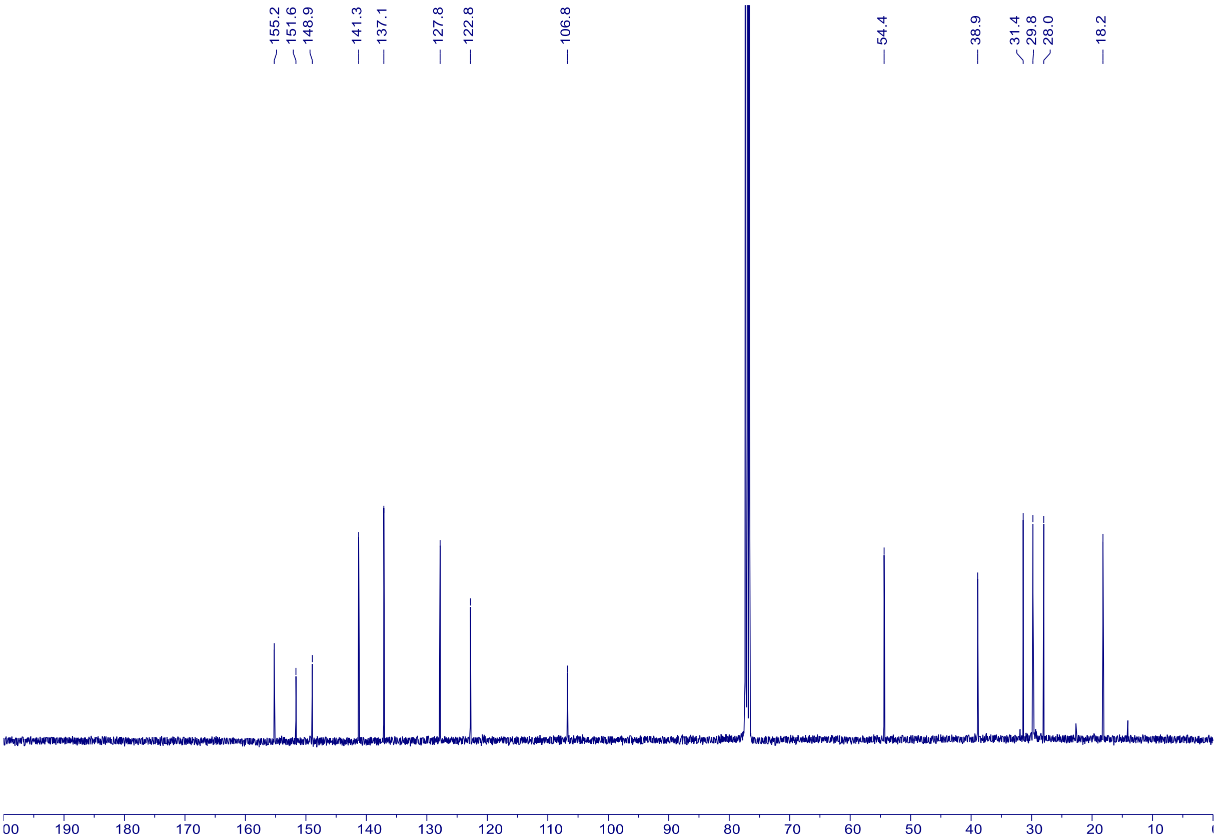

70 - ^{1}H NMR (400 MHz, $CDCl_3$)

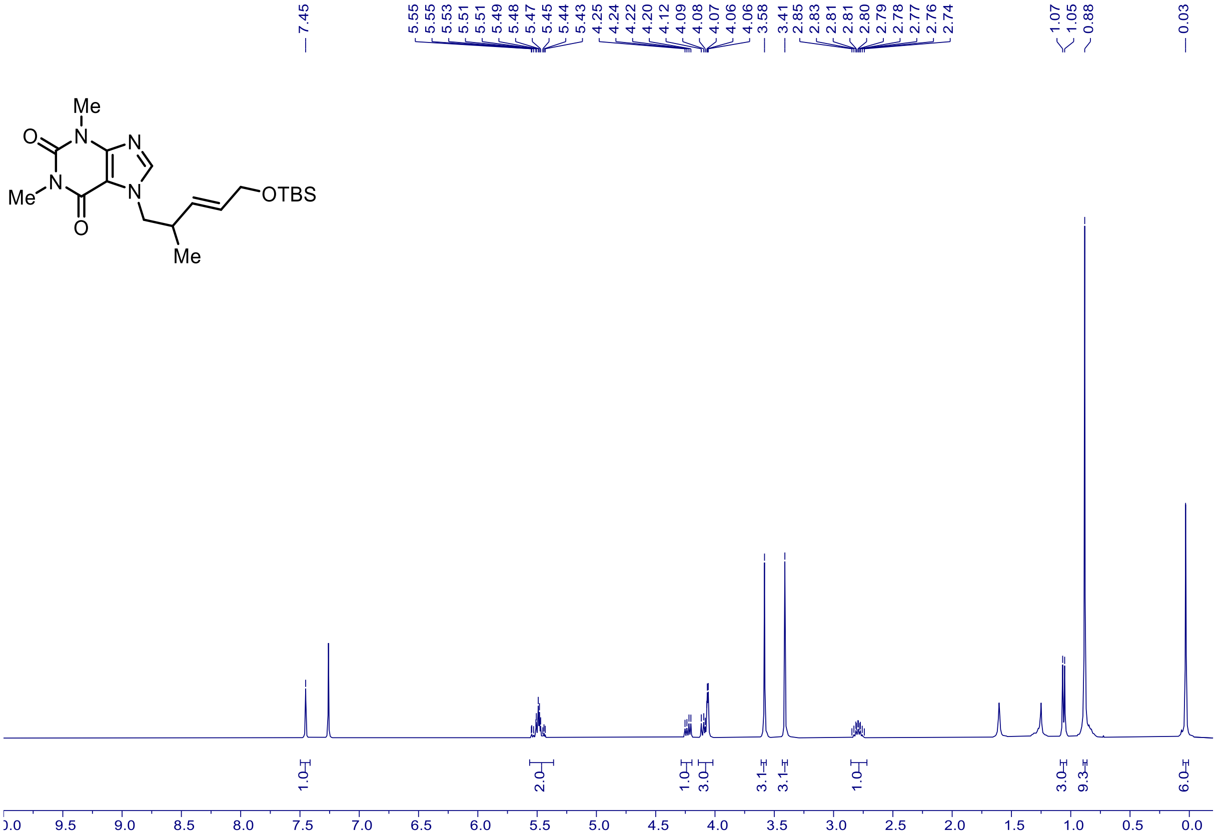

70 - ^{13}C NMR (101 MHz, $CDCl_3$)

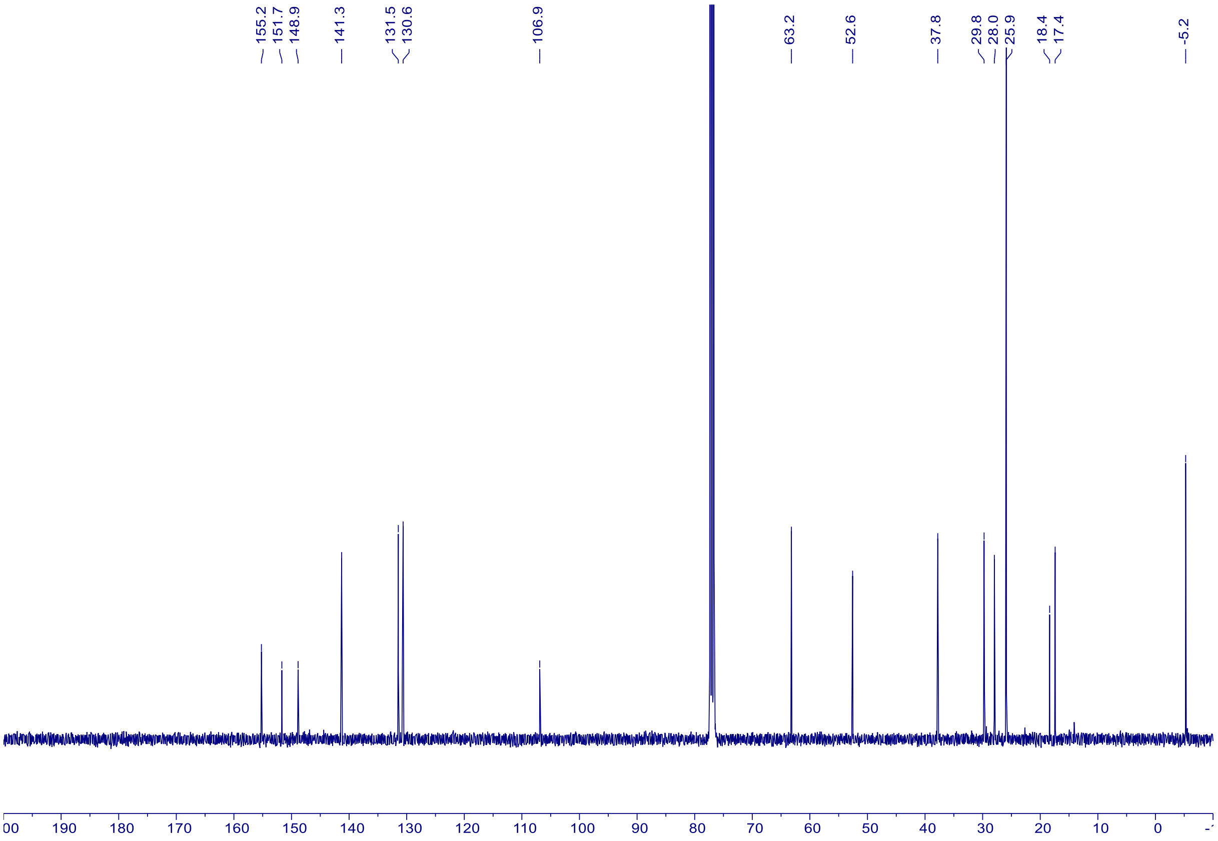

11 Reference

1. Cheung, C. W.; Ren, P.; Hu, X., Mild and Phosphine-Free Iron-Catalyzed Cross-Coupling of Nonactivated Secondary Alkyl Halides with Alkynyl Grignard Reagents. *Org. Lett.* **2014,** *16* (9), 2566-2569.
2. Lutter, F. H.; Grokenberger, L.; Benz, M.; Knochel, P., Cobalt-Catalyzed Csp3–Csp3 Cross-Coupling of Functionalized Alkylzinc Reagents with Alkyl Iodides. *Org. Lett.* **2020,** *22* (8), 3028-3032.
3. Matier, C. D.; Schwaben, J.; Peters, J. C.; Fu, G. C., Copper-Catalyzed Alkylation of Aliphatic Amines Induced by Visible Light. *J. Am. Chem. Soc.* **2017,** *139* (49), 17707-17710.
4. Tissot, M.; Body, N.; Petit, S.; Claessens, J.; Genicot, C.; Pasau, P., Synthesis of Electron-Deficient Heteroaromatic 1,3-Substituted Cyclobutyls via Zinc Insertion/Negishi Coupling Sequence under Batch and Automated Flow Conditions. *Org. Lett.* **2018,** *20* (24), 8022-8025.
5. Górski, B.; Barthelemy, A.-L.; Douglas, J. J.; Juliá, F.; Leonori, D., Copper-catalysed amination of alkyl iodides enabled by halogen-atom transfer. *Nat. Catal.* **2021,** *4* (7), 623-630.
6. Wang, G.-Z.; Jiang, J.; Bu, X.-S.; Dai, J.-J.; Xu, J.; Fu, Y.; Xu, H.-J., Copper-Catalyzed Cross-Coupling Reaction of Allyl Boron Ester with 1°/2°/3°-Halogenated Alkanes. *Org. Lett.* **2015,** *17* (15), 3682-3685.
7. Chen, K.-Q.; Wang, Z.-X.; Chen, X.-Y., Photochemical Decarboxylative C(sp3)–X Coupling Facilitated by Weak Interaction of N-Heterocyclic Carbene. *Org. Lett.* **2020,** *22* (20), 8059-8064.
8. Chen, Y.; Ma, G.; Gong, H., Copper-Catalyzed Reductive Trifluoromethylation of Alkyl Iodides with Togni's Reagent. *Org. Lett.* **2018,** *20* (15), 4677-4680.
9. Caiger, L.; Sinton, C.; Constantin, T.; Douglas, J. J.; Sheikh, N. S.; Juliá, F.; Leonori, D., Radical hydroxymethylation of alkyl iodides using formaldehyde as a C1 synthon. *Chem. Sci.* **2021,** *12* (31), 10448-10454.
10. Rao, D. S.; Reddy, T. R.; Babachary, K.; Kashyap, S., Regioselective vicinal functionalization of unactivated alkenes with sulfonium iodate(i) reagents under metal-free conditions. *Org. Biomol. Chem.* **2016,** *14* (31), 7529-7543.
11. Kyne, S. H.; Clémancey, M.; Blondin, G.; Derat, E.; Fensterbank, L.; Jutand, A.; Lefèvre, G.; Ollivier, C., Elucidating Dramatic Ligand Effects on SET Processes: Iron Hydride versus Iron Borohydride Catalyzed Reductive Radical Cyclization of Unsaturated Organic Halides. *Organometallics* **2018,** *37* (5), 761-771.
12. Yadav, J. S.; Reddy, B. V. S.; Krishna, V. H.; Swamy, T.; Kumar, G. K. N., Iodine-promoted Prins-cyclization of ketones — A facile synthesis of spirocyclic-4-iodo-tetrahydropyrans and 5,6-dihydro-2H-pyrans. *Can. J. Chem.* **2007,** *85* (6), 412-415.
13. K., S. A.; Somasekhar, B.; Kiran, I.; Paramartha, G., Axial-selectivity in Prins Cyclization Reaction: Synthesis of 4-Iodotetrahydropyrans. *Chem. Lett.* **2011,** *40* (10), 1176-1178.
14. Sakamoto, R.; Sakurai, S.; Maruoka, K., Alkylsilyl Peroxides as Alkylating Agents in the Copper-Catalyzed Selective Mono-N-Alkylation of Primary Amides and Arylamines. *Chem. Eur. J.* **2017,** *23* (38), 9030-9033.
15. Parmar, D.; Henkel, L.; Dib, J.; Rueping, M., Iron catalysed cross-couplings of azetidines – application to the formal synthesis of a pharmacologically active molecule. *Chem. Commun.* **2015,** *51* (11), 2111-2113.
16. Roagna, G.; Ascough, D. M. H.; Ibba, F.; Vicini, A. C.; Fontana, A.; Christensen, K. E.; Peschiulli, A.; Oehlrich, D.; Misale, A.; Trabanco, A. A.; Paton, R. S.; Pupo, G.; Gouverneur, V., Hydrogen Bonding Phase-Transfer Catalysis with Ionic Reactants: Enantioselective Synthesis of γ-Fluoroamines. *J. Am. Chem. Soc.* **2020,** *142* (33), 14045-14051.
17. Molander, G. A.; Traister, K. M.; O'Neill, B. T., Reductive Cross-Coupling of

Nonaromatic, Heterocyclic Bromides with Aryl and Heteroaryl Bromides. *J. Org. Chem.* **2014,** *79* (12), 5771-5780.
18. Liu, Z.; Luan, N.; Shen, L.; Li, J.; Zou, D.; Wu, Y.; Wu, Y., Palladium-Catalyzed Hiyama Cross-Couplings of Arylsilanes with 3-Iodoazetidine: Synthesis of 3-Arylazetidines. *J. Org. Chem.* **2019,** *84* (19), 12358-12365.
19. Bhonde, V. R.; O'Neill, B. T.; Buchwald, S. L., An Improved System for the Aqueous Lipshutz–Negishi Cross-Coupling of Alkyl Halides with Aryl Electrophiles. *Angew. Chem., Int. Ed.* **2016,** *55* (5), 1849-1853.
20. Duncton, M. A. J.; Estiarte, M. A.; Tan, D.; Kaub, C.; O'Mahony, D. J. R.; Johnson, R. J.; Cox, M.; Edwards, W. T.; Wan, M.; Kincaid, J.; Kelly, M. G., Preparation of Aryloxetanes and Arylazetidines by Use of an Alkyl−Aryl Suzuki Coupling. *Org. Lett.* **2008,** *10* (15), 3259-3262.
21. Barré, B.; Gonnard, L.; Campagne, R.; Reymond, S.; Marin, J.; Ciapetti, P.; Brellier, M.; Guérinot, A.; Cossy, J., Iron- and Cobalt-Catalyzed Arylation of Azetidines, Pyrrolidines, and Piperidines with Grignard Reagents. *Org. Lett.* **2014,** *16* (23), 6160-6163.
22. Dequirez, G.; Bourotte, M.; Porras de Francisco, E.; Remuiñan Blanco, M. J.; Déprez, B.; Willand, N., Microwave-Assisted Suzuki-Miyaura Cross Coupling using Nickel as Catalyst to Rapidly Access to 3-Arylazetidine. *ChemistrySelect* **2017,** *2* (28), 8841-8846.
23. Yamaguchi, T. I., K.; Hirano, T.; Shimabara, R.; Kawakubo. Y.; Sato, M.; Yamashita, J.; Yazaki, A.; Ueshima, T. Novel Pyridone Carboxylic Acid Derivative or Salt Thereof. U.S. Patent US2020062752. February 27, 2020.
24. Han, S.; Ren, X.; Wu, Q.; Liang, A.; Li, J.; Zou, D.; Wu, Y.; Wu, Y., Palladium-Catalyzed Decarboxylative Cross-Couplings of 1-Boc-3-iodoazetidine: Regioselective Access to 2-Alkynylazetidines, 3-Alkynylazetidines and 3-Vinylazetidines. *Adv. Synth. Catal.* **2018,** *360* (12), 2308-2312.
25. Di Franco, T.; Epenoy, A.; Hu, X., Synthesis of E-Alkyl Alkenes from Terminal Alkynes via Ni-Catalyzed Cross-Coupling of Alkyl Halides with B-Alkenyl-9-borabicyclo[3.3.1]nonanes. *Org. Lett.* **2015,** *17* (19), 4910-4913.
26. De Francisco, E. P. R.-B., M. J.; Bourotte, M.; Deprez, D.; Dequirez, G.; Willand, N. Novel Compounds. US Patent US2020170997. June 4, 2020.
27. Qiu, Z.; Zhu, M.; Zheng, L.; Li, J.; Zou, D.; Wu, Y.; Wu, Y., Regioselective α-benzylation of 3-iodoazetidine via Suzuki cross-coupling. *Tetrahedron Lett.* **2019,** *60* (19), 1321-1324.
28. Campbell, P. S.; Jamieson, C.; Simpson, I.; Watson, A. J. B., Practical synthesis of pharmaceutically relevant molecules enriched in sp3 character. *Chem. Commun.* **2018,** *54* (1), 46-49.
29. Li, Z.; Sun, W.; Wang, X.; Li, L.; Zhang, Y.; Li, C., Electrochemically Enabled, Nickel-Catalyzed Dehydroxylative Cross-Coupling of Alcohols with Aryl Halides. *J. Am. Chem. Soc.* **2021,** *143* (9), 3536-3543.
30. Liu, X.-G.; Zhou, C.-J.; Lin, E.; Han, X.-L.; Zhang, S.-S.; Li, Q.; Wang, H., Decarboxylative Negishi Coupling of Redox-Active Aliphatic Esters by Cobalt Catalysis. *Angew. Chem., Int. Ed.* **2018,** *57* (40), 13096-13100.
31. Guo, W.; Laserna, V.; Rintjema, J.; Kleij, A. W., Catalytic One-Pot Oxetane to Carbamate Conversions: Formal Synthesis of Drug Relevant Molecules. *Adv. Synth. Catal.* **2016,** *358* (10), 1602-1607.
32. Masanobu, K.; Akito, I.; Yasunao, K.; Yuji, W.; Haruhiko, T., Study of Reactive Intermediates in Photoinduced Electron Transfer Anti-Markovnikov Addition of Methanol to 1-Phenylcycloalkenes. *Bull. Chem. Soc. Jpn.* **1999,** *72* (5), 1049-1055.
33. Chatterjee, A.; König, B., Birch-Type Photoreduction of Arenes and Heteroarenes by Sensitized Electron Transfer. *Angew. Chem., Int. Ed.* **2019,** *58* (40), 14289-14294.

34. Carter, T. S.; Guiet, L.; Frank, D. J.; West, J.; Thomas, S. P., Iron-Catalysed Reduction of Olefins using a Borohydride Reagent. *Adv. Synth. Catal.* **2013,** *355* (5), 880-884.
35. Reddy, U. C.; Bondalapati, S.; Saikia, A. K., Stereoselective One-Pot, Three-Component Synthesis of 4-Aryltetrahydropyran via Prins−Friedel−Crafts Reaction. *J. Org. Chem.* **2009,** *74* (6), 2605-2608.
36. Goetzke, F. W.; Hell, A. M. L.; van Dijk, L.; Fletcher, S. P., A catalytic asymmetric cross-coupling approach to the synthesis of cyclobutanes. *Nat. Chem.* **2021,** *13* (9), 880-886.
37. Kuang, Y.; Wang, X.; Anthony, D.; Diao, T., Ni-catalyzed two-component reductive dicarbofunctionalization of alkenes via radical cyclization. *Chem. Commun.* **2018,** *54* (20), 2558-2561.
38. Kim, J. G.; Son, Y. H.; Seo, J. W.; Kang, E. J., Iron-Catalyzed Tandem Cyclization and Cross-Coupling Reactions of Iodoalkanes and Aryl Grignard Reagents. *Eur. J. Org. Chem.* **2015,** *2015* (8), 1781-1789.
39. Aranzaes, J. R.; Daniel, M.-C.; Astruc, D., Metallocenes as references for the determination of redox potentials by cyclic voltammetry — Permethylated iron and cobalt sandwich complexes, inhibition by polyamine dendrimers, and the role of hydroxy-containing ferrocenes. *Can. J. Chem.* **2006,** *84* (2), 288-299.
40. Roth, H. G.; Romero, N. A.; Nicewicz, D. A., Experimental and Calculated Electrochemical Potentials of Common Organic Molecules for Applications to Single-Electron Redox Chemistry. *Synlett* **2016,** *27* (05), 714-723.
